# Supplementary material for: Cholesterol efflux from C1QB-expressing macrophages is associated with resistance to chimeric antigen receptor T cell therapy in primary refractory diffuse large B cell lymphoma
Source: Nat Commun. 2024 Jun 18;15:5183. doi: 10.1038/s41467-024-49495-4 (PMC11189439; doi:10.1038/s41467-024-49495-4)
Supplement: Supplementary file 1 — Supplementary Information [file 41467_2024_49495_MOESM1_ESM.pdf]

Cholesterol efflux from C1QB-expressing macrophages is associated with resistance to chimeric antigen receptor T cell therapy in primary refractory diffuse large B-cell lymphoma

Zi-Xun Yan [1*], Yan Dong [1*], Niu Qiao [1*], Yi-Lun Zhang [1], Wen Wu [1], Yue Zhu [1], Li Wang [1], Shu Cheng [1], Peng-Peng Xu [1], Zi-Song Zhou [2], Ling-Shuang Sheng [1†], Wei-Li Zhao [1,3 †]

[*] Contributed equally

[†] Correspondence to: Wei-Li Zhao, Ling-shuang Sheng

Supplementary Figure 1.

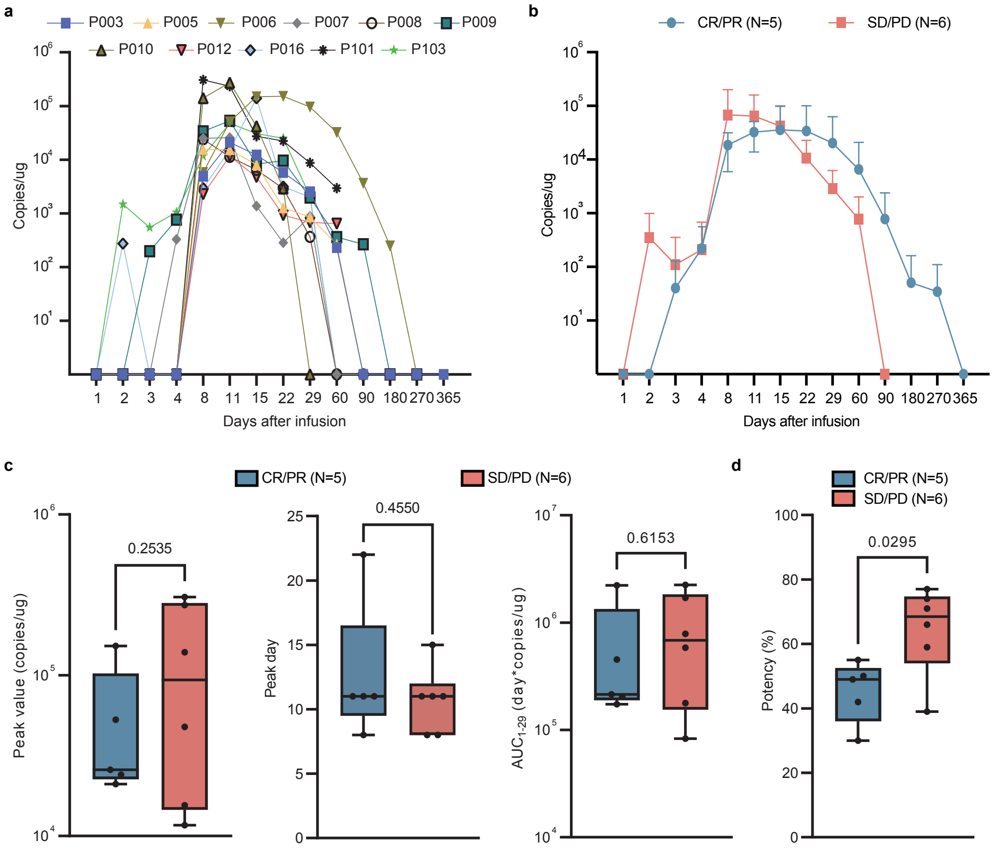

Supplementary Figure 1. Pharmacokinetic assessment.

(a) Expansion curve of CAR-T cells. (b) Concentration of CAR-T cells in CR/PR and SD/PD patients at different time points following infusion. (c) Peak value, peak day, AUC1-29, and (d) potency of cytotoxicity *ex vivo* in CR/PR (n = 5) and SD/PD (n = 6) patients. Data are shown as mean ± s.e.m. Statistical analysis was performed using unpaired t tests. CR, complete response; PR, partial response; SD, stable disease; PD, progressive disease. CR/PR patients (n = 5), SD/PD patients (n = 6).

Supplementary Figure 2.

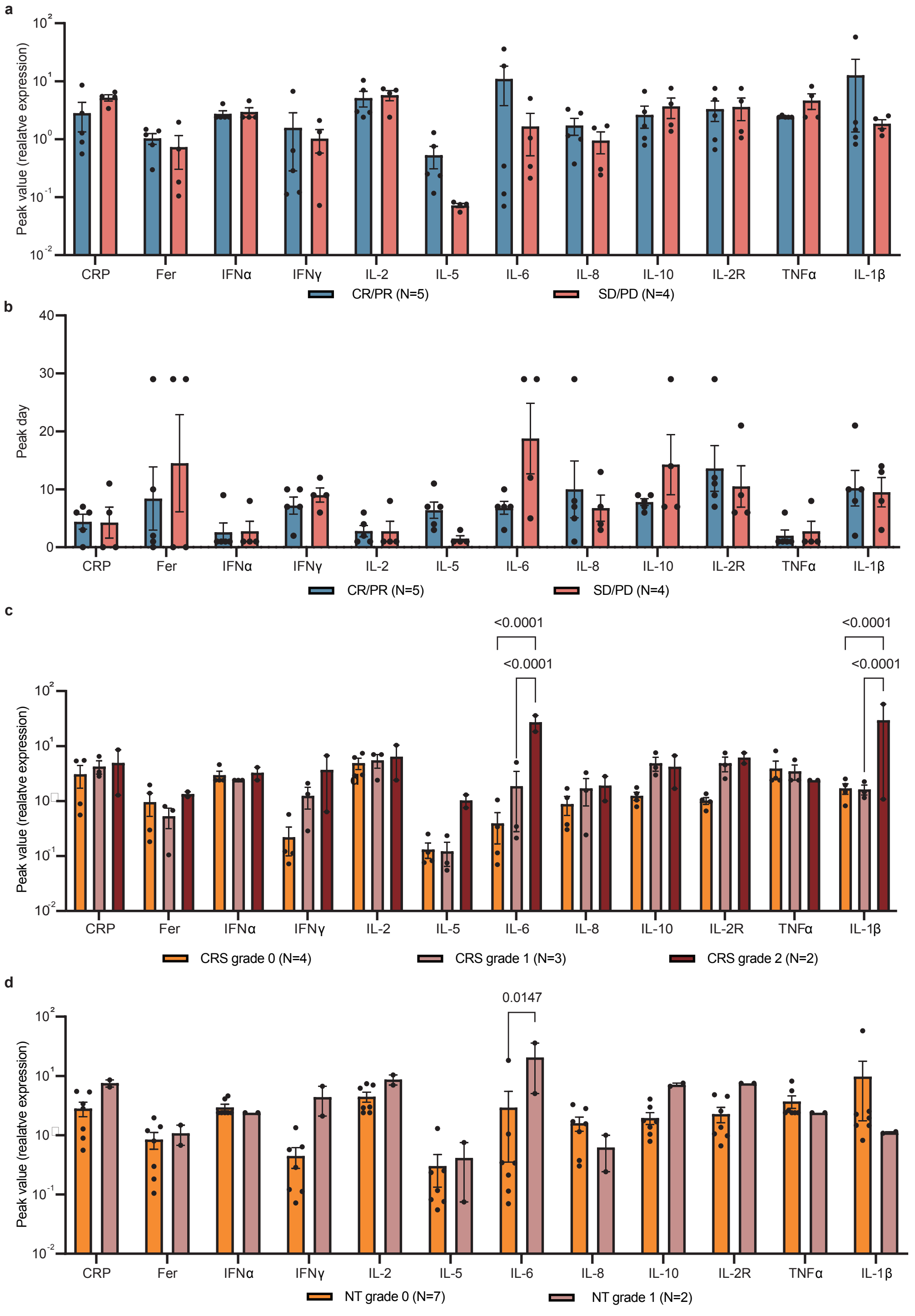

Supplementary Figure 2. Peak values/day of serum biomarkers.

(a) Peak value relative expression of serum biomarkers in CR/PR and SD/PD patients.

(b) Peak days of serum biomarkers in CR/PR and SD/PD patients. (c) Peak value relative expression of serum biomarkers in patients with no CRS, grade 1 CRS, and grade 2 CRS. (d) Peak value relative expression of serum biomarkers in patients with grade 1 NT and without NT. Data are shown as mean ± s.e.m. Statistical analysis was performed using 2-way ANOVA test. CR, complete response; PR, partial response; SD, stable disease; PD, progressive disease; CRS, cytokine release syndrome; NT, neurotoxicity. CR/PR patients (n = 5), SD/PD patients (n = 6).

Supplementary Figure 3.

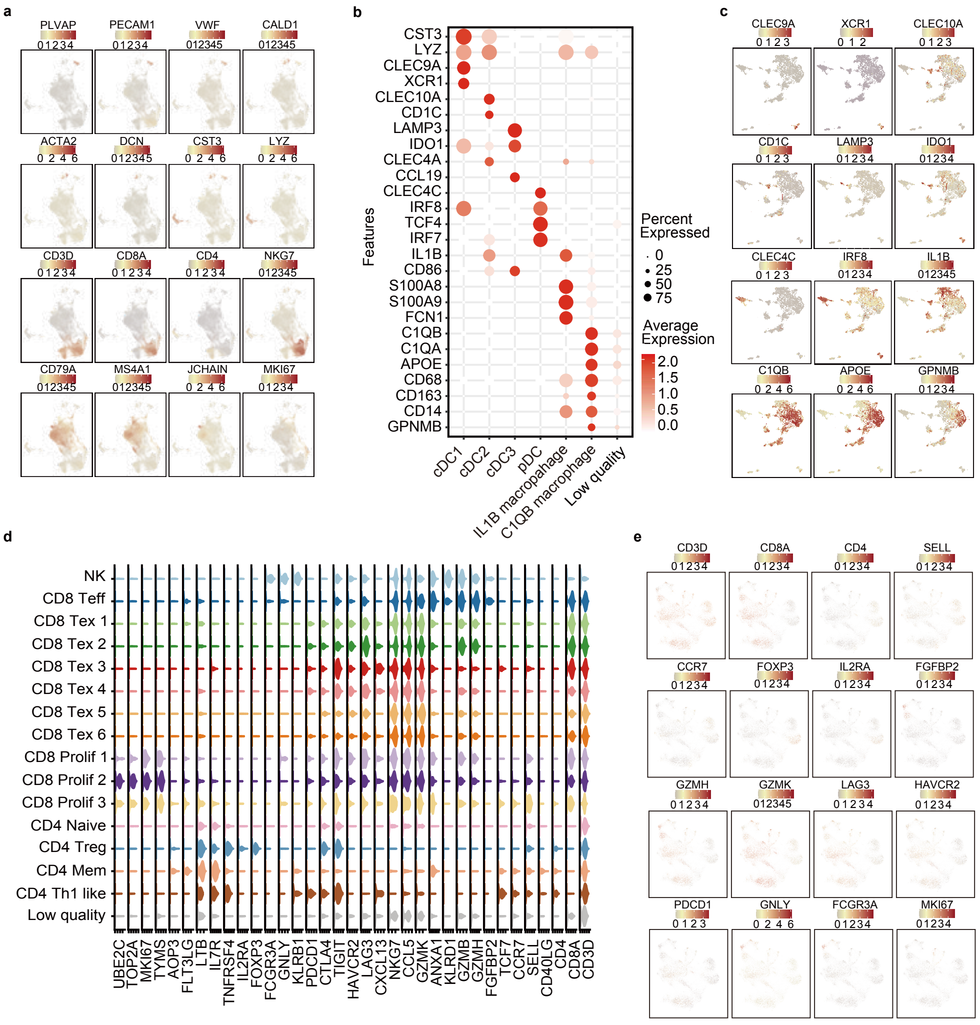

Supplementary Figure 3. Gene expression characteristics of different subclusters.

(a) Gene expression profiles of five clusters. (b) Dot plot showing the gene expression profiles of seven subclusters of myeloid cell population. Color bar indicates expression level, and dot size indicates percentage. (c) UMAP of gene expression profiles of seven subclusters of myeloid cell population. (d) Dot plot showing the gene expression profiles of seven subclusters of myeloid cell population. Color bar indicates expression level, and dot size indicates percentage. (e) UMAP of gene expression profiles of 16 subclusters of the NK/T-cell population.

Supplementary Figure 4.

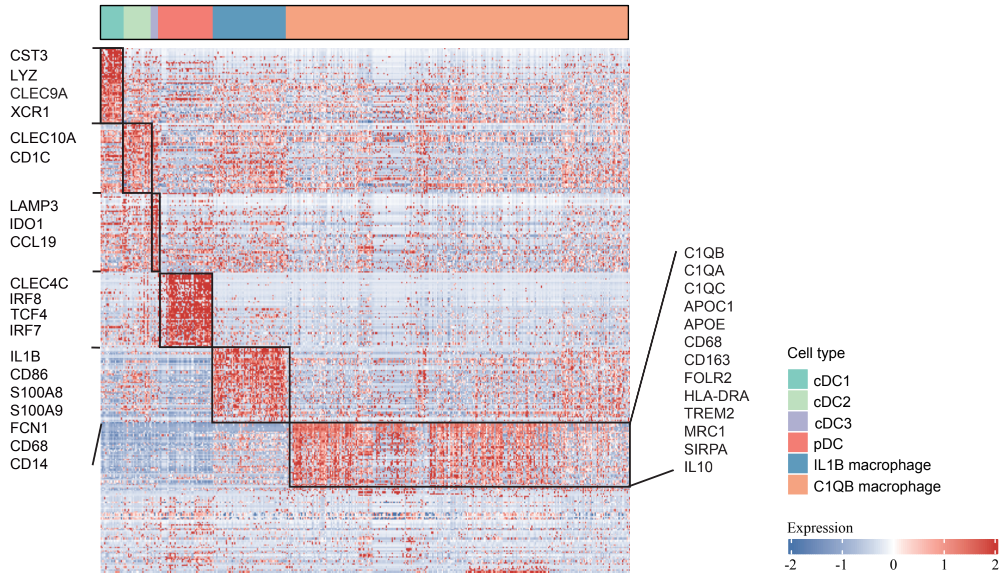

Supplementary Figure 4. Heatmap showing the gene expression profiles of myeloid cell population.

Supplementary Figure 5.

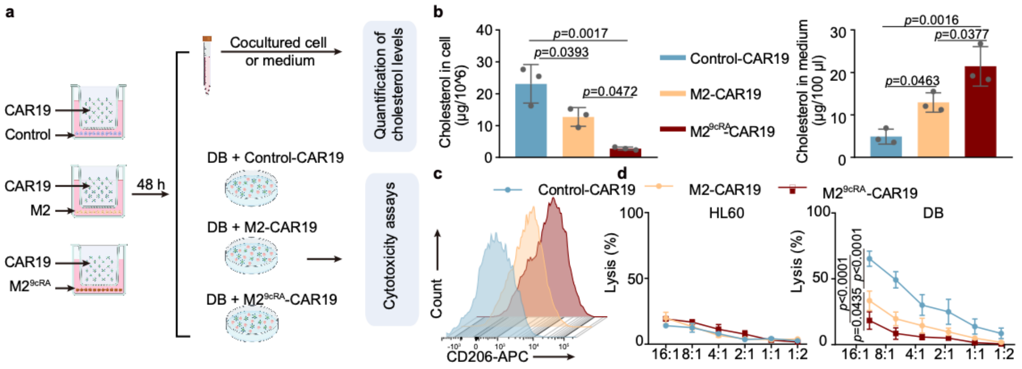

Supplementary Figure 5. Increased cholesterol efflux form M2 macrophages using 9cRA pharmacological approaches enhance the immunosuppression effects.

(a) In vitro, coculture models were established to evaluate the effects of M2 macrophages and M2 macrophages with 9-cis-Retinoic acid (9cRA) on CAR-T cell cytotoxicity against CD19-expressing DLBCL cells (DB cells). (b) The impact of CAR19 cells on the total or secreted cholesterol levels of M2 macrophages was evaluated. (c) The expression of CD206 on macrophages in the indicated groups was evaluated through flow cytometry. (d) The cytotoxic effect of CAR19 cells on CD19-expressing DLBCL cells (DB) and CD19 non-expressing acute promyelocytic leukemia cells (HL60) was assessed using a luciferase-based CTL assay. Data are presented as mean ± s.e.m. Statistical analysis was performed using two-way ANOVA with Tukey's multiple comparison tests. Control-CAR19 vs M2-CAR19 (p < 0.0001), Control-CAR19 vs M2^{9cRA}-CAR19 (p < 0.0001), M2-CAR19 vs M2^{9cRA}-CAR19 (p = 0.0435).

Supplementary Figure 6.

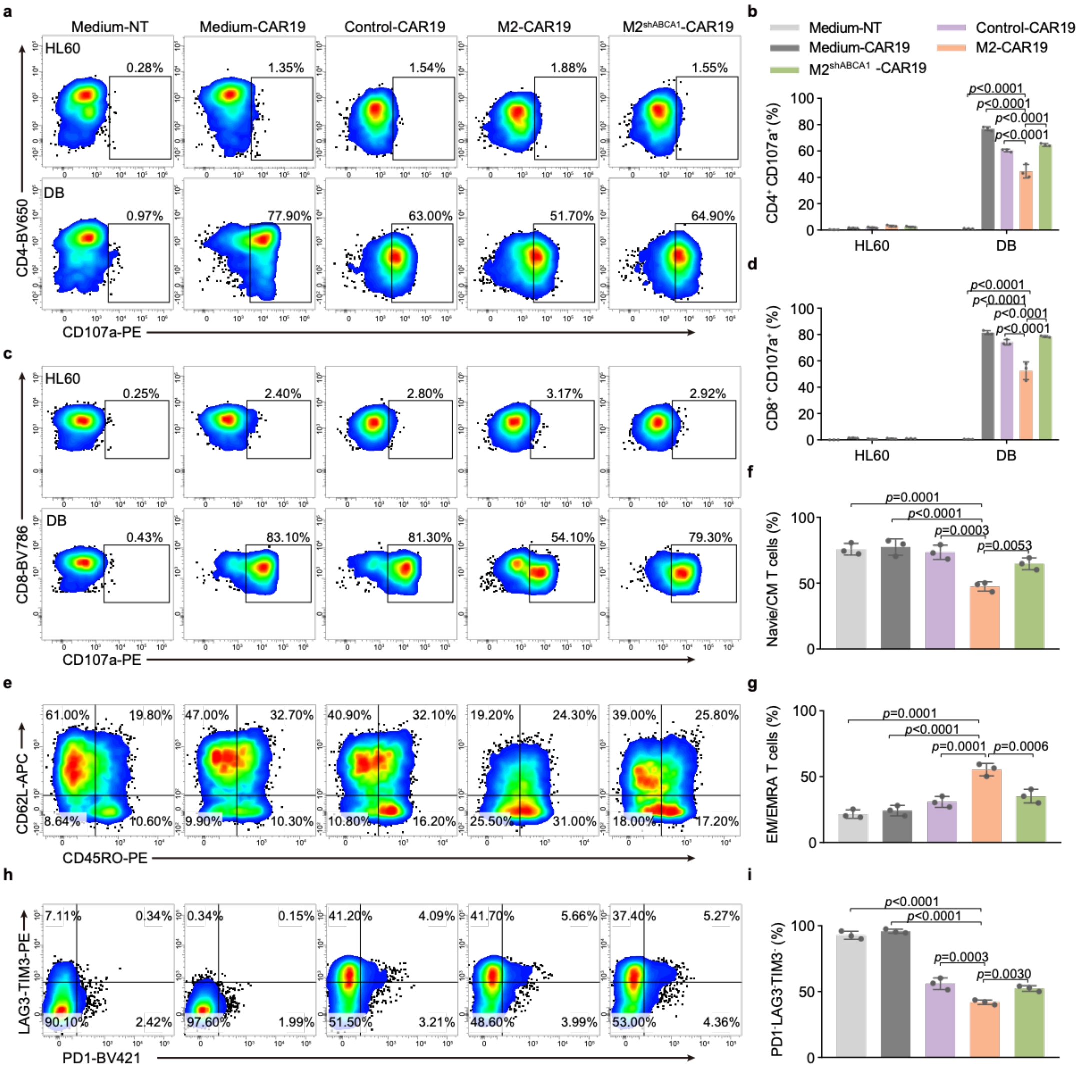

Supplementary Figure 6. Function of CAR-T cells.

(a) Flow cytometry analysis of degranulation of CD4+ CAR-T cells. (b) Statistical analysis of CD4+ CAR-T cell degranulation. (c) Flow cytometry analysis of degranulation of CD8+ CAR-T cells. (d) Statistical analysis of CD8+ CAR-T cell degranulation. (e) Flow cytometry analysis of CAR-T cell subtypes. (f) Statistical analysis of CAR-T cell subtypes indicating reduced percentages of naive, central memory T-cells (Tcm) in the M2-CAR19 group, which were restored following cholesterol efflux inhibition by ABCA1 knockdown. (g) Statistical analysis of CAR-T cell subtypes indicating increased percentages of effector memory T-cells (Tem), and terminal effector T-cells (Temra) in the M2-CAR19 group, which were restored

following cholesterol efflux inhibition by ABCA1 knockdown. (h) Flow cytometry analysis of CAR-T cell subtypes. (i) Statistical analysis of CAR-T cell subtypes indicating a reduced percentage of $PD1^{-}LAG3^{-}TIM3^{-}$ cells in the M2-CAR19 group, which was restored by ABCA1 inhibition.

Supplementary Figure 7.

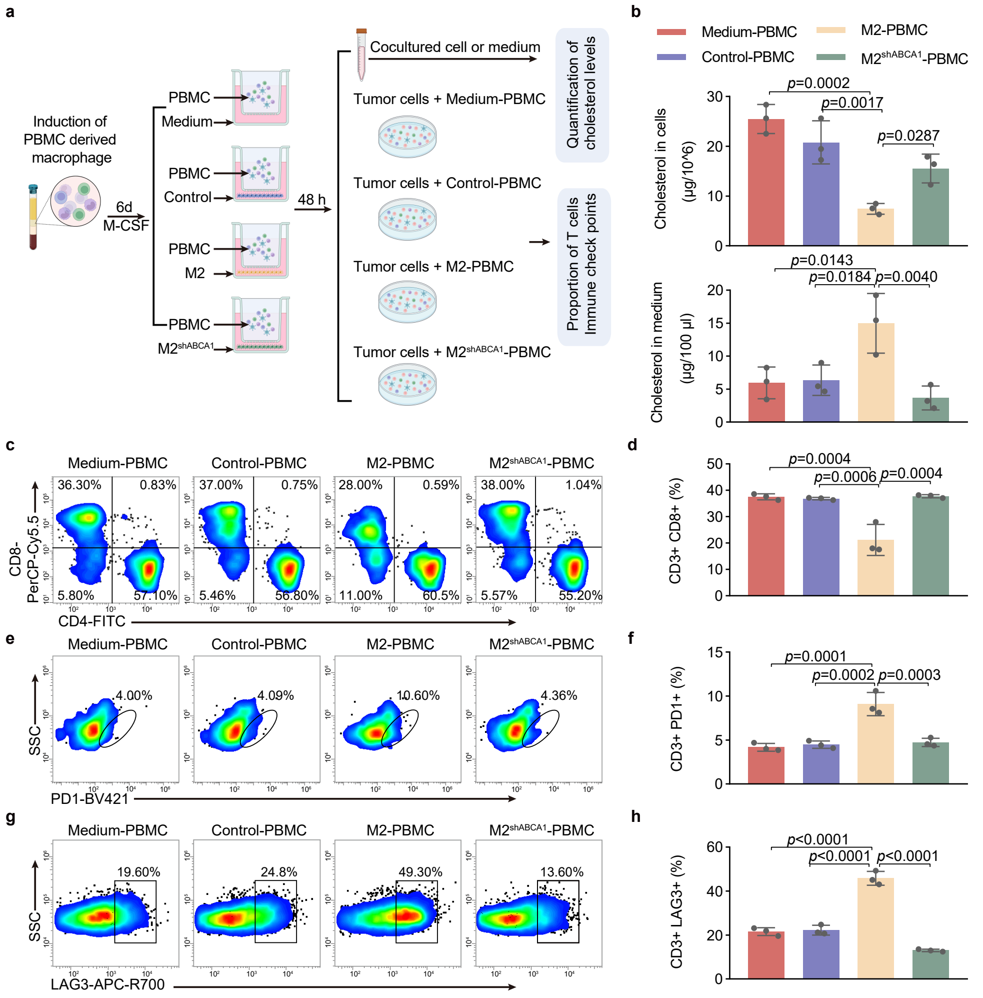

Supplementary Figure 7. T-cell exhaustion induced by cholesterol efflux from PBMC-derived M2 macrophages.

(a) Flowchart of the *in vitro* assay. PBMCs were cocultured with control, M2 macrophages, and ABCA1-knockdown M2 macrophages ($M2^{shABCA1}$) for 48 h, in the presence of DB cells to mimic the TME. Cholesterol levels in cells and culture supernatant, the percentages of T-cells, and immune checkpoint genes were quantified. (b) Quantification of total cholesterol levels in cells and medium. Flow cytometry analysis of the percentages of $CD8^+$ T-cells (c, d), $PD1^+$ cells (e, f), and $LAG3^+$ T-cells (g, h).

Supplementary Figure 8.

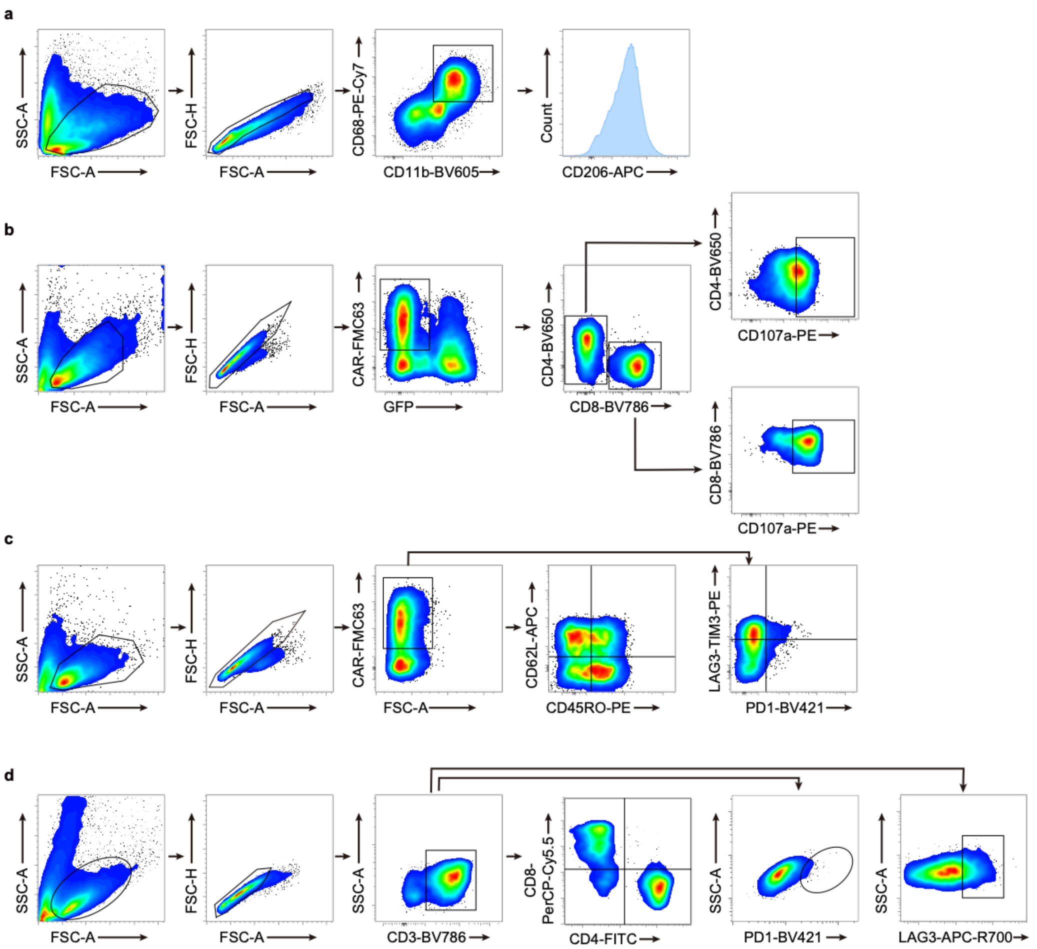

Supplementary Figure 8. Gating strategy to identify each subset.

Gating strategy in representative flow cytometric analysis to identify $PD1^{+}$ and $LAG3^{+}$ T-cells (a), M2 phenotype (b), $CD4^{+}/CD8^{+}$ CAR-T cells (c), $PD1^{-}LAG3^{-}TIM3^{-}$ cells (d).

Graphical Abstract

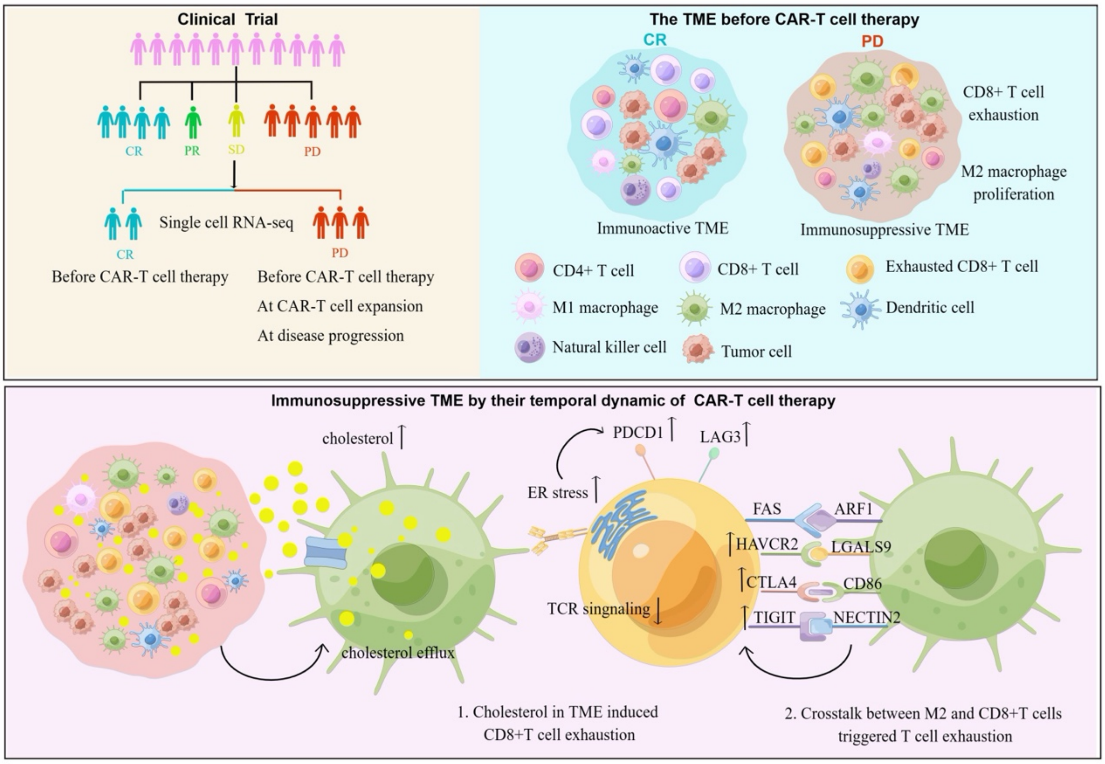

Highlights

1. Increased C1QB macrophage and $CD8^+$ T-cell exhaustion are associated with CAR-T cell therapy response in DLBCL;

2. T cells present an immunosuppressive and exhaustion state at disease progression upon CAR-T cell therapy;

3. Crosstalk between C1QB macrophage and T cells in lipid metabolism and immune checkpoints activation provokes T-cell exhaustion;

4. Cholesterol efflux of C1QB macrophage induces T/CAR-T cell exhaustion mediated by ER stress pathway.

Supplementary Table 1. Baseline characteristics

Characteristics	N=12
**Age (years)**	
Median (range)	60 (45-71)
**Sex**	
Male	8 (66.7%)
Female	4 (33.3%)
**Pathologic diagnosis**	
Diffuse large B-cell lymphoma, not otherwise specified	10 (83.3%)
Epstein-Barr virus positive diffuse large B-cell lymphoma	1 (8.3%)
High grade B-cell lymphoma, double-hit	1 (8.3%)
**Ann Arbor stage**	
I or II	3 (25.0%)
III or IV	9 (75.0%)
**Number of extranodal lesions**	
<2	8 (66.7%)
>=2	4 (33.3%)
**Lactic dehydrogenase**	
Normal	1 (8.3%)
Elevated	11 (91.7%)
**Performance status (ECOG)**	
0-1	10 (83.3%)
2-4	2 (16.7%)
**International prognostic index**	
Low risk (0-2)	3 (25.0%)
High risk (3-5)	9 (75.0%)
**Hans classification**	
Germinal center B-cell	6 (50%)
Non-germinal center B-cell	6 (50%)
**Double expressor lymphoma**	
Yes	8 (66.7%)
No	4 (33.3%)
**Genetic subtype**	
BN2	3/10 (33.3%)
EZB	1/10 (10.0%)
MCD	1/10 (10.0%)
Other	5/10 (50.0%)
**Tumor burden (SPD)**	
<5000mm^2	8 (66.7%)
>=5000mm^2	4 (33.3%)
**Bridging therapy**	
Yes	5 (41.7%)
No	7 (58.3%)
**Response to first-line therapy**	
Complete Response (CR)	0 (0%)
Partial Response (PR)	2 (16.7%)
Stable Disease (SD)	7 (58.3%)
Progressive Disease (PD)	3 (25.0%)

Supplementary Table 2. Treatment emergent adverse events

	Any grade	Grade 1	Grade 2	Grade 3	Grade 4
**Any Study Treatment Related TEAEs**	12 (100.0%)	1 (8.3%)	1 (8.3%)	2 (16.7%)	8 (66.7%)
**Laboratory examination abnormalities**	12 (100.0%)	1 (8.3%)	1 (8.3%)	2 (16.7%)	8 (66.7%)
Hypogammaglobulinaemia	10 (83.3%)	7 (58.3%)	3 (25.0%)	0 (0%)	0 (0%)
Leukopenia	10 (83.3%)	0 (0%)	3 (25.0%)	4 (33.3%)	3 (25.0%)
Lymphopenia	10 (83.3%)	0 (0%)	3 (25.0%)	2 (16.7%)	5 (41.7%)
Neutropenia	10 (83.3%)	1 (8.3%)	0 (0%)	4 (33.3%)	5 (41.7%)
Thrombocytopenia	5 (41.7%)	0 (0%)	2 (16.7%)	1 (8.3%)	2 (16.7%)
Blood fibrinogen decreased	4 (33.3%)	1 (8.3%)	3 (25.0%)	0 (0%)	0 (0%)
Blood lactate dehydrogenase increased	3 (25.0%)	3 (25.0%)	0 (0%)	0 (0%)	0 (0%)
Aspartate aminotransferase increased	2 (16.7%)	2 (16.7%)	0 (0%)	0 (0%)	0 (0%)
Activated partial thromboplastin time prolonged	1 (8.3%)	1 (8.3%)	0 (0%)	0 (0%)	0 (0%)
Alpha hydroxybutyrate dehydrogenase increased	1 (8.3%)	1 (8.3%)	0 (0%)	0 (0%)	0 (0%)
C-reactive protein increased	1 (8.3%)	0 (0%)	1 (8.3%)	0 (0%)	0 (0%)
Immunology test abnormal	1 (8.3%)	0 (0%)	0 (0%)	1 (8.3%)	0 (0%)
**Blood and lymphatic system disorders**	8 (66.7%)	1 (8.3%)	2 (16.7%)	5 (41.7%)	0 (0%)
Anaemia	7 (58.3%)	1 (8.3%)	2 (16.7%)	4 (33.3%)	0 (0%)
Coagulopathy	1 (8.3%)	0 (0%)	1 (8.3%)	0 (0%)	0 (0%)
Febrile neutropenia	1 (8.3%)	0 (0%)	0 (0%)	1 (8.3%)	0 (0%)
**Metabolism and nutrition disorders**	8 (66.7%)	1 (8.3%)	7 (58.3%)	0 (0%)	0 (0%)
Hypoalbuminaemia	5 (41.7%)	1 (8.3%)	4 (33.3%)	0 (0%)	0 (0%)
Hypokalaemia	3 (25.0%)	0 (0%)	3 (25.0%)	0 (0%)	0 (0%)
Hypocalcaemia	1 (8.3%)	1 (8.3%)	0 (0%)	0 (0%)	0 (0%)
Hypochloraemia	1 (8.3%)	1 (8.3%)	0 (0%)	0 (0%)	0 (0%)
Hypomagnesaemia	1 (8.3%)	1 (8.3%)	0 (0%)	0 (0%)	0 (0%)
**Immune system disorders**	6 (50.0%)	4 (33.3%)	2 (16.7%)	0 (0%)	0 (0%)
Cytokine release syndrome	6 (50.0%)	4 (33.3%)	2 (16.7%)	0 (0%)	0 (0%)
**General disorders**	2 (16.7%)	2 (16.7%)	0 (0%)	0 (0%)	0 (0%)
Oedema peripheral	1 (8.3%)	1 (8.3%)	0 (0%)	0 (0%)	0 (0%)
**Nervous system disorders**	2 (16.7%)	2 (16.7%)	0 (0%)	0 (0%)	0 (0%)
Neurotoxicity	2 (16.7%)	2 (16.7%)	0 (0%)	0 (0%)	0 (0%)
**Cardiac disorders**	1 (8.3%)	0 (0%)	1 (8.3%)	0 (0%)	0 (0%)
Sinus tachycardia	1 (8.3%)	0 (0%)	1 (8.3%)	0 (0%)	0 (0%)
**Musculoskeletal and connective tissue disorders**	1 (8.3%)	0 (0%)	1 (8.3%)	0 (0%)	0 (0%)
Back pain	1 (8.3%)	0 (0%)	1 (8.3%)	0 (0%)	0 (0%)
**Renal and urinary disorders**	1 (8.3%)	0 (0%)	1 (8.3%)	0 (0%)	0 (0%)
Obstructive nephropathy	1 (8.3%)	0 (0%)	1 (8.3%)	0 (0%)	0 (0%)

Supplementary Table 3. Results of other secondary endpoints of patients.

Patient ID	BORR	DOR (days)	TTR (days)	TTCR (days)	Tmax (days)	AUC (days*copies/ug DNA)	Cmax (copies/ug DNA)	CD3+/CD4+/CAR+ at Tmax (cells/uL)	CD3+/CD8+/CAR+ at Tmax (cells/uL)	CD8+/CD4+ ratio	CD19
P003	CR	NA	30	543	11	214095.5	21070	49.51	11.78	0.24	positive
P005	PD	NA	NA	NA	8	178298.8	15546	3.22	12.14	3.77	positive
P006	CR	278.00	26	26	22	2224992	152159	219.1	15.92	0.07	positive
P007	CR	NA	27	27	11	204081	25815	7.79	12.76	1.64	positive
P008	CR	NA	28	28	8	173948.3	24052	2.27	3.54	1.56	positive
P009	CR	NA	29	29	11	452823	52928	24.94	36.04	1.45	positive
P010	PD	NA	NA	NA	11	1702459	273189	6.03	39.06	6.48	positive
P012	PD	NA	NA	NA	11	83086.5	11690	3.95	1.65	0.42	positive
P016	PR	67.00	26	NA	15	786093	139059	2.98	4.96	1.66	positive
P101	PR	30.00	29	NA	8	2242533	306683	28.89	630.45	21.82	positive
P103	PR	NA	29	NA	11	584565.5	47710	2.02	6.49	3.21	positive

Abbreviations: BORR, best objective response rate; DOR, duration of response; TTR, time to response; TTCR, time to complete response; Tmax, time to maximum concentration; AUC, area under the plasma concentration-time curve; Cmax, maximum plasma concentration.

Supplementary Table 4. Serum cytokine antibodies

**Antibody**	**Company/Clone**	**Catalog number**	**Dilution /amount (μg/mL)**	**Valiadation**
IL-1β Capture Antibody	Raisecare/1H2	RM001001	1	https://www.qdraisecare.com/chanpin/
IL-1β Detection Antibody	Raisecare/13C5	RM001002	1	https://www.qdraisecare.com/chanpin/
IL-2 Capture Antibody	Raisecare/6F8	RM001003	1	https://www.qdraisecare.com/chanpin/
IL-2 Detection Antibody	Raisecare/19F1	RM001004	1	https://www.qdraisecare.com/chanpin/
IL-5 Capture Antibody	Raisecare/2H2	RM001007	1	https://www.qdraisecare.com/chanpin/
IL-5 Detection Antibody	Raisecare/11C10	RM001008	1	https://www.qdraisecare.com/chanpin/
IL-6 Capture Antibody	Raisecare/6G6	RM001009	1	https://www.qdraisecare.com/chanpin/
IL-6 Detection Antibody	Raisecare/8F5	RM001010	1	https://www.qdraisecare.com/chanpin/
IL-8 Capture Antibody	Raisecare/18C4	RM001011	1	https://www.qdraisecare.com/chanpin/
IL-8 Detection Antibody	Raisecare/12E3	RM001012	1	https://www.qdraisecare.com/chanpin/
IL-10 Capture Antibody	Raisecare/4F7	RM001013	1	https://www.qdraisecare.com/chanpin/
IL-10 Detection Antibody	Raisecare/5C5	RM001014	1	https://www.qdraisecare.com/chanpin/
IFN-γ Capture Antibody	Raisecare/4D6	RM001019	1	https://www.qdraisecare.com/chanpin/
IFN-γ Detection Antibody	Raisecare/9H3	RM001020	1	https://www.qdraisecare.com/chanpin/
TNF-α Capture Antibody	Raisecare/5A3	RM001021	1	https://www.qdraisecare.com/chanpin/
TNF-α Detection Antibody	Raisecare/8F2	RM001022	1	https://www.qdraisecare.com/chanpin/
IFN-α Capture Antibody	Raisecare/2H7	RM001023	1	https://www.qdraisecare.com/chanpin/
IFN-α Detection Antibody	Raisecare/10E7	RM001024	1	https://www.qdraisecare.com/chanpin/

Supplementary Table 5. Flow cytometry antibodies.

Antibody	Company	Catalog number	Dilution /amount	Validation
FITC anti-human CD4	BD Biosiences	566320	1:50	https://www.bdbiosciences.com/zh-cn/products/reagents/flow-cytometry-reagents/research-reagents/single-color-antibodies-ruo/fitc-mouse-anti-human-cd4.566320
BV421 anti-human PD1	BD Biosiences	562516	1:50	https://www.bdbiosciences.com/zh-cn/products/reagents/flow-cytometry-reagents/research-reagents/single-color-antibodies-ruo/bv421-mouse-anti-human-cd279-pd-1.562516
PerCP-Cy5.5 anti-human CD8	BD Biosiences	565310	1:50	https://www.bdbiosciences.com/zh-cn/products/reagents/flow-cytometry-reagents/research-reagents/single-color-antibodies-ruo/percp-cy-5-5-mouse-anti-human-cd8.565310
APC-R700 anti-human LAG3	BD Biosiences	565774	1:50	https://www.bdbiosciences.com/zh-cn/products/reagents/flow-cytometry-reagents/research-reagents/single-color-antibodies-ruo/apc-r700-mouse-anti-human-lag-3-cd223.565774
BV786 anti-human CD3	BD Biosiences	563799	1:50	https://www.bdbiosciences.com/zh-cn/products/reagents/flow-cytometry-reagents/research-reagents/single-color-antibodies-ruo/bv786-mouse-anti-human-cd3.563799
APC anti-human CD206	BD Biosiences	550889	1:20	https://www.bdbiosciences.com/zh-cn/products/reagents/flow-cytometry-reagents/research-reagents/single-color-antibodies-ruo/apc-mouse-anti-human-cd206.550889
PE-Cy7 anti-human CD68	BD Biosiences	565595	1:50	https://www.bdbiosciences.com/zh-cn/search-results?searchKey=PE-Cy7%20anti-human%20CD68
BV605 anti-human CD11b	BD Biosiences	562721	1:50	https://www.bdbiosciences.com/zh-cn/products/reagents/flow-cytometry-reagents/research-reagents/single-color-antibodies-ruo/bv605-mouse-anti-human-cd11b.562721
PE anti-human	BD Biosiences	555493	1:50	https://bdbiosciences.com/zh-cn/products/reagents/flow-cytometry-

CD45RO				reagents/research-reagents/single-color-antibodies-ruo/pe-mouse-anti-human-cd45ro.555493
APC anti-human CD62L	BD Biosiences	566791	1:50	https://bdbiosciences.com/zh-cn/products/reagents/flow-cytometry-reagents/research-reagents/single-color-antibodies-ruo/apc-mouse-anti-human-cd62l.566791
APC anti-FMC63 Antibody	ACROBio systems	AY54A1	1:50	https://www.acrobiosystems.cn/P24276-APC-Labeled_Monoclonal_Anti-FMC63_Antibody_Mouse_IgG1_Y45_Site-specific_conjugation_0.03%25_Proclin.html
FITC anti-FMC63 Antibody	ACROBio systems	FY45P1	1:50	https://www.acrobiosystems.cn/P22936-FITC-Labeled_Monoclonal_Anti-FMC63_Antibody_Mouse_IgG1_Y45_HEK293.html
PE anti-human LAG3	BD Biosiences	565616	1:50	https://www.bdbiosciences.com/zh-cn/products/reagents/flow-cytometry-reagents/research-reagents/single-color-antibodies-ruo/pe-mouse-anti-human-lag-3-cd223.565616
PE anti-human TIM3	BD Biosiences	563422	1:50	https://www.bdbiosciences.com/zh-cn/products/reagents/flow-cytometry-reagents/research-reagents/single-color-antibodies-ruo/pe-mouse-anti-human-tim-3-cd366.563422
PE anti-human CD107a	BD Biosiences	555801	1:50	https://www.bdbiosciences.com/zh-cn/products/reagents/flow-cytometry-reagents/research-reagents/single-color-antibodies-ruo/pe-mouse-anti-human-cd107a.555801
BV650 anti-human CD4	BD Biosiences	563737	1:50	https://www.bdbiosciences.com/zh-cn/products/reagents/flow-cytometry-reagents/research-reagents/single-color-antibodies-ruo/bv650-mouse-anti-human-cd4.563737
BV786 anti-human CD8	BD Biosiences	563823	1:50	https://www.bdbiosciences.com/zh-cn/products/reagents/flow-cytometry-reagents/research-reagents/single-color-antibodies-ruo/bv786-mouse-anti-human-cd8.563823

A Phase 1 Open-label, Single-arm, Multi-center Study of JWCAR029 (CD19-Targeted Chimeric Antigen Receptor T Cells) in Patients with Primary Refractory Diffuse Large B-cell Lymphoma

Clinical Trial Protocol

**Investigational Product:**	JWCAR029
**Name of Investigational Product:**	CD19-targeted Chimeric Antigen Receptor (CAR) T Cells for Injection
**Study Protocol ID:**	JWCAR029-003
**Version No.:**	Version 4.0
**Sponsor:**	Shanghai Mingju Biotechnology Co., Ltd.

The information provided in this document is confidential and belongs to the proprietary property of Shanghai Mingju Biotechnology Co., Ltd. Without prior written permission of Mingju, this document may not be reproduced, or disclosed to others, or used for any other unauthorized purposes (unless otherwise required by relevant laws) by any individuals accessing or reviewing it. If a breach of the above provision, either actual or suspected, Shanghai Mingju Biotechnology Co., Ltd must be notified promptly.

Sponsor Signature Page

A Phase 1 Open-label, Single-arm, Multi-center Study of JWCAR029 (CD19-Targeted Chimeric Antigen Receptor T Cells) in Patients with Primary Refractory Diffuse Large B-cell Lymphoma

We, the undersigned, have reviewed this protocol and agree that this protocol contains all information necessary for the conduct of this clinical trial.

Sponsor's Signature:	Date:
__________________________	__________________________
__________________________	__________________________
__________________________	__________________________

COORDINATING PRINCIPAL INVESTIGATOR SIGNATURE PAGE

A Phase 1 Open-label, Single-arm, Multi-center Study of JWCAR029 (CD19-Targeted Chimeric Antigen Receptor T Cells) in Patients with Primary Refractory Diffuse Large B-cell Lymphoma

I, the undersigned, have read this study protocol and agree to perform the duties of principal investigator according to the principles laid down in "Good Clinical Practice" (GCP) and "Guideline for Good Clinical Practice of the International Conference on Harmonisation", and conduct this clinical trial in compliance with the study protocol to ensure successful implementation of the clinical study.

____

Coordinating Principal Investigator's Name (Print)

________________________________ ____________________________

Coordinating Principal investigator's Signature Date

PRINCIPAL INVESTIGATOR SIGNATURE PAGE

A Phase 1 Open-label, Single-arm, Multi-center Study of JWCAR029 (CD19-Targeted Chimeric Antigen Receptor T Cells) in Patients with Primary Refractory Diffuse Large B-cell Lymphoma

I, the undersigned, have read this study protocol and agree to perform the duties of principal investigator according to the principles laid down in "Good Clinical Practice" (GCP) and "Guideline for Good Clinical Practice of the International Conference on Harmonisation", and conduct this clinical trial in compliance with the study protocol to ensure successful implementation of the clinical study.

Site No.: ____________________

____

Principal Investigator's Name (Print)

________________________________ ____________________________

Principal Investigator's Signature Date

TABLE OF CONTENTS

Coordinating Principal Investigator Signature Page 3

Principal Investigator Signature Page 4

Table of contents 5

Listing of Tables and Figures 9

Protocol Synopsis 10

List of Abbreviations 17

1. Introduction 21

1.1 B-cell Non-Hodgkin Lymphoma 21
1.2 CD19 as a Therapeutic Target 21
1.3 CD19-targeted Chimeric Antigen Receptors 21
1.4 Investigational Product JWCAR029 22
1.5 Clinical Experience with JWCAR029 and Relevant CAR+ T Cell Products 22

2. Justification of the Study 24

2.1 Justification of JWCAR029 Dose 24
2.2 Justification of Lympho-depleting Chemotherapy Regimen 25
2.3 Justification of Study Design 26

3. Study Objectives and Endpoints 27

4. Study Design and Plan 28

4.1 Overview of Study Design 28
4.2 Study Procedures 28
4.3 Duration of Study and Estimated Duration of Participation 29
4.4 Study Oversight 29
4.4.1 Safety Review Committee *29*
4.5 Protocol Product Deviation Plan 30
4.6 Additional Treatment Cycles and Re-treatment 30

5. Study Population 30

5.1 Inclusion Criteria 30
5.2 Exclusion Criteria 31
5.3 Childbearing Potential and Contraception Requirements 33
5.4 Removal of Subjects from Treatment or Study 34
5.4.1 Screen Failure *34*
5.4.2 Treatment Discontinuation by Subjects before Receiving the Study Treatment *34*
5.4.3 Discontinuation of Further Study Treatment by Subject *35*
5.4.4 Withdrawal of Subjects from Study *35*
5.4.5 Replacement of Subjects *35*

6 Study Treatment 35

6.1 Leukapheresis 35
6.2 Anticancer Therapy During the Period of Leukapheresis and Lympho-depleting Chemotherapy 36
6.3 Lympho-depleting Chemotherapy 36
6.4 Study Treatment: JWCAR029 36

6.4.1 Dose and Dosing Regimen ... 37
6.4.2 Other Treatments Prior to Administration of JWCAR029 ... 37
6.4.3 JWCAR029 Preparation and Cell Recovery ... 37
6.4.4 Administration of JWCAR029 ... 37
6.4.5 Use of Non-standard Products ... 38
6.4.6 Concomitant Medications ... 38
6.5 Prohibited Medications ... 39
6.6 Recommendations on Care Support, Other Therapies and Monitoring ... 40

7 Management of Potential Risks and Treatment Toxicities ... 40

7.1 Cytokine Release Syndrome ... 40
7.2 Fever ... 44
7.3 Neurotoxicity ... 44
7.4 Macrophage Activation Syndrome ... 47
7.5 Infusion-related Reactions ... 47
7.6 Tumor Lysis Syndrome (TLS) ... 47
7.7 B-cell Dysplasia ... 47
7.8 Graft-Versus-Host Disease (GVHD) ... 48
7.9 Uncontrolled T-cell Hyperplasia ... 48
7.10 Replication-competent Lentivirus, Clonality and Insertional Mutagenesis ... 48
7.11 Risks Associated with Lympho-depleting Chemotherapy ... 48

8 Study Assessments and Procedures ... 48

8.1 Study Procedures ... 49
8.2 Screening ... 49
8.3 Leukapheresis ... 50
8.4 Study Visits ... 50
8.4.1 Bridging Anticancer Therapy between Leukapheresis and JWCAR029 Infusion 50
8.4.2 Pre-treatment Assessment (after Enrollment and before Lympho-depleting Chemotherapy) ... 51
8.4.3 Lympho-depleting Chemotherapy ... 53
8.4.3.1 Treatment Criteria ... 53
8.4.3.2 Lympho-depleting chemotherapy (must be completed within 2 days to 7 days prior to administration of JWCAR029) ... 53
8.4.4 Criteria for JWCAR029 Treatment ... 54
8.4.5 JWCAR029 Treatment ... 55
8.4.6 Post-treatment Follow-up Visits ... 57
8.5 Unscheduled Visits ... 59
8.6 Assessments after Disease Progression/Relapse ... 59
8.7 Early Withdrawal ... 60
8.8 Long-term Follow-up ... 60
8.9 Study Assessments ... 60
8.9.1 Safety Evaluation ... 60
8.9.2 Physical Examination ... 60
8.9.3 MMSE ... 60
8.9.4 Vital Signs ... 60
8.9.5 Neurological Assessment and Examination ... 61
8.9.6 Response Assessment ... 61
8.9.7 Clinical Laboratory Tests ... 61
8.9.8 ECOG Performance Status ... 62

8.9.9 MUGA/Echocardiogram ... *62*
8.9.10 ECG ... *62*
8.9.11 Study Samples ... *62*

9 Safety Oversight and Reporting ... 63

9.1 Definitions ... 63
9.1.1 Adverse Event ... *63*
9.1.2 Serious Adverse Event ... *63*
9.2 Clinical Laboratory Test Abnormalities and Other Assessment Abnormalities ... 65
9.3 Evaluation of AEs and SAEs ... 65
9.3.1 Grading and Severity of AEs ... *66*
9.3.2 Relationship to Investigational Product ... *66*
9.4 Collection and Reporting of AEs ... 66
9.4.1 Documentation of Diagnosis and Signs & Symptoms ... *68*
9.4.2 Clinical Laboratory Abnormalities and Other Abnormal Assessment Findings ... *68*
9.4.3 Documentation of Serious Adverse Events ... *69*
9.4.4 Reporting of Deaths ... *69*
9.4.5 Pregnancy ... *69*
9.5 Reporting and Follow-up of AEs and SAEs ... 70

10 Statistical Methods ... 70

10.1 Comprehensive Consideration ... 70
10.2 Data Analysis Sets ... 70
10.2.1 Safety Set ... *70*
10.2.2 Modified Intent-to-Treat (mITT) Set ... *70*
10.2.3 Other Analysis Sets ... *71*
10.3 Data Handling Conventions ... 71
10.4 Planned Analyses ... 71
10.4.1 Subject Disposition and Baseline Characteristics ... *71*
10.4.2 Safety Analysis ... *71*
10.4.2.1 Adverse events ... *71*
10.4.2.2 Laboratory data ... *72*
10.4.2.3 Immunogenicity data ... *73*
10.4.2.4 Other safety data ... *73*
10.4.2.5 Concomitant medications ... *73*
10.4.3 Efficacy Analysis of Study Treatment ... *73*
10.4.3.1 Efficacy analysis ... *73*
10.4.4 Pharmacokinetic Analysis ... *74*
10.4.5 Quality Attributes and Process Performance Attributes of JWCAR029 Product *74*
10.5 Sample Size Considerations ... 75
10.6 Timing of Analysis ... 75

11 Data Management ... 75

11.1 Data Collection System ... 75
11.2 Data Quality ... 75

12 Study Administration ... 75

12.1 Regulatory and Ethical Considerations ... 75
12.1.1 Regulatory Audit ... *75*
12.1.2 Approval of Institutional Review Board (IRB)/ Ethics Committee (EC) ... *76*

12.1.3 Subject Informed Consent 76
12.2 Obligations of the Investigator 76
12.2.1 Responsibilities of the Investigator 76
12.2.2 Reporting Responsibilities of the Investigator 77
12.3 Access to Information Monitoring 77
12.4 On-site Audits and Regulatory Inspections of Study Site 77
12.5 Protocol Deviations 77
12.6 Quality Assurance and Quality Control 77
12.7 Publication on Study Progress 78
12.8 Termination of Study 78
12.9 Termination of Study Site 78
12.10 Document Retention 78
12.11 Confidential Information 79
12.12 Publication Plan 79

13 Contact Information 80

13.1 Emergency Medical Contact 80
13.2 Pharmacovigilance 80

14 References 81

Appendix A .... Schedule of Activities (SoA) 87

Appendix B .... Response Criteria 92

Appendix C ...COCKCROFT-GAULT Formula for Calculation of Estimated Creatinine Clearance 93

Appendix D .... Mini-Mental State Examination 94

Appendix E .... IPI (International Prognostic Index) Score 97

Appendix F .... Formula for Calculation of Body Surface Area (BSA) 98

LISTING OF TABLES AND FIGURES

Table 1: Objectives and Endpoints of the Study 27
Table 2: Study Summary 28
Table 3: Reporting Periods for Concomitant Medications in Subjects Treated with JWCAR029 38
Table 4: CRS Grading Criteria (Lee 2014) 42
Table 5: Washout Period Prior to lympho-depleting Chemotherapy 53
Table 6: Analytes for Clinical Laboratory Assessments 62
Table 7: ECOG Performance Status 62
Table 8: Timeline for Reporting of AEs 67

Figure 1: Schematic Diagram of Clinical Trial Design 28
Figure 2: Approaches to CRS Management in NHL (Adapted/Modified from Lee 2014; Neelapu SS 2017) 43
Figure 3: Management Principles for Neurotoxicity (Neelapu SS 2017) 47

PROTOCOL SYNOPSIS

**Protocol ID:** JWCAR029-003	**Investigational Product:** JWCAR029
**Title of Study Protocol:** A Phase 1 Open-label, Single-arm, Multi-center Study of JWCAR029 (CD19-Targeted Chimeric Antigen Receptor T Cells) in Patients with Primary Refractory Diffuse Large B-cell Lymphoma	
**Sponsor:** Shanghai Mingju Biotechnology Co., Ltd	**Study Phase:** Phase 1
**Study Population**	The target study population are subjects with primary refractory diffuse large B-cell lymphoma (DLBCL).
**Rationale of Study**	Current therapies for primary refractory DLBCL do not produce satisfactory results. Although rituximab-based regimens have improved the outcomes of these patients, there still exists an urgent need for the development of novel therapeutic approaches for primary refractory DLBCL.<br><br>CD19 is a 95-kDa glycoprotein present on pre-B lymphocytes until the plasma cell stage of differentiation. It is a member of the immunoglobulin family and also a component of the signaling complex on the B cell surface. Expressed in most B-cell malignancies including B-cell non-Hodgkin lymphomas (B-NHL), CD19 has become a therapeutic target of great interest. Importantly, CD19 is not expressed by hematopoietic stem cells or other normal tissues, except for the B-cell lineages.<br><br>CD19-specific chimeric antigen receptors (CARs) are a single-chain variable fragment (scFv), a fusion protein consisting of a binding domain and a signal transduction domain of the 4-1BB and CD 3 ζ chain. Expression of the CD19-targeted CAR in autologous T cells has been achieved by means of in vitro transduction using recombinant retroviral or lentiviral vectors. Upon expression on the membrane surface of T cells, the CAR recognizes CD19-expressing lymphoma cells, thereby mediating CD19-specific tumor cell lysis, cytokine secretion, and T cell proliferation. In clinical studies, anti-CD19 CARs have demonstrated excellent efficacy in adult and pediatric subjects with refractory B-cell acute lymphoblastic leukemia (ALL) and B-cell NHL.
**Study Objectives**	Primary:<br>• To evaluate the safety of JWCAR029 in subjects with primary R/R DLBCL<br>Secondary:<br>• To evaluate the anti-tumor activity of JWCAR029<br>• To evaluate the durability of anti-tumor activity of JWCAR029<br>• To describe the pharmacokinetic (PK) and pharmacodynamic (PD) characteristics of JWCAR029<br>• To evaluate the immunogenicity of JWCAR029<br>• To evaluate the impact of the properties of JWCAR029 on safety, PK, and anti-tumor activity
**Study Design**	This is a Phase 1 open-label, single-arm multi-center study in China to investigate the safety of JWCAR029 in subjects with R/R DLBCL, in which approximately 12 subjects will receive JWCAR029 infusion at a dose of 100×10^6 CAR+ T cells. After JWCAR029 infusion, safety and other follow-up data will be collected continuously, followed by two years of follow-up after the end of treatment. During the study, the Safety Review Committee (SRC) will observe and evaluate the safety

	of the subjects continuously and make a decision on how the study should be continued. The dose of 100 × 10^6 CAR+ T cells selected for primary R/R DLBCL subjects is based on the results of a previous Phase 1/2 study in subjects with refractory DLBCL. In study JWCAR029-001, dose escalation was completed at four pre-determined dose levels and two final recommended doses were determined. In study JWCAR029-002, the efficacy and safety of JWCAR029 at the two dose levels in R/R DLBCL were established, with superior safety and efficacy in the 100 × 10^6 dose group versus the 150 × 10^6 dose group as judged from differences in the results of descriptive analysis. As the volume of CAR-T cell infusion needs to be calculated based on the concentration and dose of CAR-T cells in the solution for infusion, the fixed dose of 100 × 10^6 is recommended as the clinical dose considering the feasibility and convenience of clinical operations.<br><br>**<u>Study Procedures</u>**<br><br>All subjects will undergo baseline disease assessment, including physical examination of the neck, chest, abdomen and pelvis, complete blood count (CBC) with contrast-enhanced computed tomography (CT), PET-CT, bone marrow aspiration (BMA) and bone marrow biopsy (BMB) (only for subjects with palpable masses and if deemed feasible by the investigator), etc. At enrollment, all subjects will undergo leukapheresis to generate the JWCAR029 product. If the first leukapheresis does not generate JWCAR029 for a subject successfully, additional leukapheresis may be performed after discussion with the Sponsor. During the period when a subject is waiting for JWCAR029 generation, the necessity of bridging anti-cancer therapy for the subject will be determined based on the investigator's assessment. Provided bridging therapy is administered to a subject, baseline assessment must be performed for the subject again to ensure that the subject meets all inclusion/exclusion criteria and can be included in the study.<br><br>The subjects will receive lympho-depleting chemotherapy prior to infusion of JWCAR029. After infusion, AEs and laboratory abnormalities will be collected continuously for safety evaluation. Then, the follow-up results will be collected over a follow-up period of 2 years to evaluate the safety, efficacy, disease status, other anti-cancer therapies and survival for the subjects. Follow-up of long-term safety and overall survival will also continue for the subjects even after disease progression or start of any other anti-cancer therapy.<br><br>During the treatment and follow-up periods, all adverse events (AEs) and serious adverse events (SAEs) will be recorded. Toxicities will be graded according to the National Cancer Institute (NCI) Common Terminology Criteria for Adverse Events (CTCAE) version 5.0, except for cytokine release syndrome (CRS), which is graded based on the modified criteria system (Davila 2014, Lee 2014, Neelapu 2017).<br><br>**<u>Clinical Trial Committee</u>**<br><br>**The Safety Review Committee (SRC)** is formed by the principal investigator and the medical monitor, statisticians and the study physicians of Shanghai Mingju Biotechnology Co., Ltd. The SRC will observe and evaluate the safety of the subjects continuously during the study and make a decision as to whether the study should be continued (including continuing with the dose, reducing the dose, or discontinuing the study, etc.).
**Dose Groups and Subject Enrollment**	This trial is a Phase 1 single-arm study, in which the subjects will receive an infusion of JWCAR029 at the dose of 100 × 10^6 CAR-T cells.
**Study Population**	The target study population consists of subjects with primary R/R DLBCL, who should meet the following inclusion and exclusion criteria at screening and will be assessed against these criteria again before lympho-depleting chemotherapy.

	**Inclusion Criteria**<br>The subjects must meet **all** of the following criteria to be included in this study:<br>1. Aged ≥ 18 years<br>2. Having signed the Informed Consent Form (ICF)<br>3. Patients with primary refractory DLBCL who failed to achieve complete response (CR) after first-line treatment using anthracyclines and rituximab other CD20-targeted drugs<br>a. Stable disease (SD) after minimum 3 cycles of first-line treatment, or<br>b. Biopsy-proven residual disease despite partial response (PR) after 6 cycles of first-line treatment, or<br>c. Progressive disease (PD) on any course of first-line treatment<br>4. PET-positive lesions established according to the Lugano criteria<br>5. An Eastern Cooperative Oncology Group (ECOG) performance status (PS) score of 0 or 1<br>6. Adequate organ function:<br>a. Adequate bone marrow function as assessed by the investigator to receive lympho-depleting chemotherapy [absolute neutrophil count (ANC) ≥ 1000/μL, platelet count ≥ 75,000/μL, and absolute lymphocyte count ≥ 100/μL]<br>b. Serum creatinine ≤ 1.5 × upper limit of normal (ULN) or creatinine clearance (Cockcroft and Gault) > 50 mL/min (excluding serum creatinine clearance decreased due to compression from lymphoma mass)<br>c. Alanine aminotransferase (ALT) ≤ 5 × ULN and total bilirubin < 2.0 mg/dL (or < 3.0 mg/dL for subjects with Gilbert's syndrome or lymphoma involving the liver)<br>d. Pulmonary function: dyspnea ≤ CTCAE grade 1 and SpO_2 ≥ 92% in room air<br>e. Cardiac function: left ventricular ejection fraction (LVEF) ≥ 50% shown by echocardiogram (ECHO) or multi-gated radionuclide angiography (MUGA) within 1 month after enrollment<br>7. Vascular access adequate for leukapheresis<br>8. For subjects previously treated with CD19-targeted therapy, biopsy-proven CD19 expression in the lymphoma lesions<br>9. Women of childbearing potential (all women who can get pregnant physiologically) must agree to use highly effective methods of birth control for one year after JWCAR029 infusion<br>10. Male subjects with partners of childbearing potential must agree to use effective barrier methods of birth control for one year after JWCAR029 infusion<br><br>**Exclusion Criteria**<br>Subjects meeting **any** of the following criteria will be excluded from this study:<br>1. DLBCL patients who were treated by second-line and late therapies<br>2. Patients with lymphoma who are known to be negative for CD19 expression<br>3. Primary central nervous system lymphoma (subjects with secondary CNS lymphoma are allowed to be enrolled)<br>4. History of other malignancies that have not achieved complete response for at least 2 years (this 2-year limitation may be exempted for the following conditions: non-melanoma skin cancer, completely resected stage I tumors with a low probability of recurrence, limited-stage prostate cancer post-treatment, and biopsy-confirmed cervical carcinoma in situ, or squamous intraepithelial

lesions evidenced by PAP smears)

5. Presence of the following at screening:
   a. Positivity for hepatitis B surface antigene (HBsAg) (regardless of whether there is an increase in the copies of hepatitis B virus DNA);
   b. Positivity for hepatitis B core antibody (HBcAb) with an increase in the copies of hepatitis B virus DNA
   c. Infection with hepatitis C virus, human immunodeficiency virus (HIV) or syphilis
6. Deep vein thrombosis (DVT) (cancer embolism or thrombus) or pulmonary embolism (PE) within 3 months before the signing of the Informed Consent Form
7. Ongoing anticoagulant therapy for deep vein thrombosis (DVT) or pulmonary embolism (PE) within 3 months before the signing of the Informed Consent Form
8. Uncontrolled systemic fungal, bacterial, viral or other infections
9. Acute or chronic graft-versus-host disease (GVHD)
10. History of any cardiovascular disease within the past 6 months: New York Heart Association (NYHA) Class III/IV heart failure, cardiac angioplasty or stenting , myocardial infarction, unstable angina, or other clinically significant heart disease
11. History or current evidence of clinically significant CNS diseases at screening, such as epilepsy, epileptic seizures, paralysis, aphasia, strokes, severe brain injury, dementia, Parkinson's disease, cerebellar disease, organic brain syndrome (OBS) or mental disorders
12. Pregnant or lactating women. Women of childbearing potential must have a negative result of serum pregnancy test within 48 hours before start of lympho-depleting chemotherapy
13. Use of any of the following medications or therapies within the specified time before leukapheresis:
    a. Alemtuzumab within 6 months before leukapheresis
    b. Cladribine within 3 months before leukapheresis
    c. Fludarabine within 3 months before leukapheresis
    d. Anti-CD20 monoclonal antibodies within 7 days before leukapheresis
    e. Venetoclax within 4 days before leukapheresis
    f. Idelalisib within 2 days before leukapheresis
    g. Lenalidomide within 1 day before leukapheresis
    h. Corticosteroids at therapeutic doses (defined as prednisone or equivalent > 20 mg/day) within 7 days before leukapheresis or within 72 hours before administration of JWCAR029. However, physiological replacement and topical/inhaled steroids are allowed
    i. Low-dose chemotherapy (such as vincristine, rituximab, cyclophosphamide ≤ 300mg/m^2), if required to control the patient's condition after leukapheresis, must have stopped for ≥ 7 days before the lympho-depleting chemotherapy
    j. Non-lymphocytotoxic chemotherapeutic agents (e.g., doxorubicin, vincristine, gemcitabine, oxaliplatin, carboplatin, etoposide) within 1 week before leukapheresis. Use of oral chemotherapeutics (including lenalidomide and ibrutinib) before leukapheresis does not preclude enrollment if it has been > 3 half-lives
    k. Use of lymphocytotoxic chemotherapeutics (such as cyclophosphamide, ifosfamide, bendamustine, chlorambucil, melphalan) within 2 weeks before leukapheresis
    l. Use of an investigational product within 4 weeks before leukapheresis.

	However, this criterion does not preclude enrollment if the subject did not respond to or progressed on the investigational treatment and it has been > 3 half-lives before leukapheresis<br>m. Therapies for the treatment of GVHD within 4 weeks before leukapheresis and JWCAR029 infusion, such as calcineurin inhibitor, methotrexate or other chemotherapeutics, mycophenolate mofetil, rapamycin, thalidomide, and immunosuppressants like anti-TNF, anti-IL-6 or anti-IL-6R antibodies)<br>n. Donor lymphocyte infusion (DLI) within 6 weeks before administration of JWCAR029<br>o. Chemotherapy within 6 weeks before leukapheresis, involving large storage areas for bone marrow, such as the sternum or pelvis. In this case, the subject is eligible for enrollment if PD occurred at the site of radiotherapy or presence of PET-positive lesions at other unirradiated sites. Radiotherapy for a single lesion is allowed within 2 weeks before leukapheresis if PET-positive lesions are present at other unirradiated sites<br>p. Allogeneic hematopoietic stem cell transplantation (allo-HSCT) within 90 days before leukapheresis<br>14. Presence of any factors that would affect the subject's compliance with the protocol in the investigator's judgment, including uncontrollable medical, psychological, family, sociological or geographical factors; or unwilling or inability to follow the procedures required in the study protocol<br>15. Previous treatment with CAR+T cells or other genetically modified T cells
**Number of Subjects and Duration of Study**	This study is planned to enroll approximately 12 subjects. Considering the possibility that the subjects may receive non-standard therapies and be replaced during the course of the study, the number of subjects actually enrolled may exceed 12.<br>Enrollment is expected to continue for 9 to 12 months and follow-up, starting after JWCAR029 infusion, is expected to continue for 24 months for each subject. Therefore, the total duration of the study is estimated to be about 33 to 36 months.
**Study Sites**	Ruijin Hospital, Shanghai Jiao Tong University School of Medicine and other 1 to 2 centers
**Investigational Product, Dose, and Mode of Administration**	The investigational product JWCAR029, which is preserved in dimethyl sulfoxide (DMSO)-containing culture medium as frozen cell suspension consisting of autologous T lymphocytes expressing CD19-specific chimeric antigen receptors (CARs), is supplied to the subjects for intravenous infusion directly (on Days 2 to 7 after completion of lympho-depleting chemotherapy).<br>The subjects will receive the JWCAR029 product as an IV infusion at the dose of 100×10^6 CAR-T cells. If the JWCAR029 product was not manufactured at the dose specified for this study, the dose may be reduced to the interest of the subject's safety after discussion by the investigator and the Sponsor. Data analysis will be performed according to the dosage schedule actually implemented for the subjects.
**Safety Assessment**	Adverse events (AEs), serious adverse events (SAEs), and laboratory abnormalities (type, frequency, and severity) will be collected. Adverse events of special interest (AESIs) will also be collected, including Grade 2 and above cytokine release syndrome (CRS), neurotoxicity, etc.
**Efficacy Evaluation**	Efficacy evaluation will be conducted through PET-CT and/or enhanced CT scans at Day 29 (PET-CT and/or enhanced CT scans may be performed at Days 22 to 29), as well as at 3, 6, 9, 12, 18 and 24 months (±14 days) after JWCAR029 infusion, or until disease progression, withdrawal from the study, or start of replacement therapy.<br>The efficacy outcome can be assessed as complete response (CR), partial response (PR), stable disease (PD) or progressive disease (PD) as per the Lugano criteria (Cheson 2014), and the duration of response will be determined according to the

	procedures specified in the Schedule of Activities (SoA) for the clinical trial.
**Other Assessments**	The following assessments will be performed at specific time points:<br>**PK/PD Assessment:**<br>PK assessment of JWCAR029 is performed through quantitative PCR (qPCR) to detect the transgene copy number of JWCAR029 in peripheral blood and through flow cytometry to determine the number and immunophenotype of JWCAR029 CAT+ T cells in peripheral blood. In vivo PD assessment is performed by means of flow cytometry to determine the number of CD19+ B cells in the peripheral blood. The PK/PD assessment of JWCAR029, starting after cell infusion following lympho-depleting chemotherapy, will continue until the end of study (EOS).<br>**Immunogenicity Assessment:**<br>This assessment will be performed in subjects showing immune response to JWCAR029, including detection of anti-drug antibodies (ADA).<br>**Cytokine Assessment:**<br>Cytokine assessment includes serum cytokines associated with CRS treated by JWCAR029.
**Statistical Methods**	**Justification of Sample Size**<br>This is a Phase 1 safety study, which is planned to enroll 12 subjects. Considering the possibility that the subjects may receive non-standard therapies and be replaced during the course of the study, the number of subjects actually enrolled may exceed 12.<br><br>**Statistical Analysis Sets:**<br>Safety Set (SS) is defined as all subjects who received any dose of JWCAR029 treatment.<br>Modified intent-to-treat (mITT) set will be used for the analysis of efficacy endpoints. This set is defined the same as SS but excludes those subjects who received non-standard investigational product.<br>Additional data sets will be defined to include subjects with the necessary baseline and study measurements. These data sets will provide results about specific parameters of interest.<br><br>**Methods of Statistical Analysis:**<br>Various AEs and SAEs occurring during the treatment period will be analyzed using descriptive statistics. AEs will be coded by System Organ Class (SOC) and Preferred Term (PT) using MedDRA (Version 21.0 or a new version subsequently released), and graded as per NCI CTCAE (Version 5.0). Descriptive statistics will include the number of subjects with AE, corresponding incidence rate, etc.<br>For the results of laboratory tests and ECG and for safety variables like vital signs, ECOG performance status, nervous system examination and MMSE test, their changes and abnormalities during the study period will be summarized using descriptive statistics.<br>Efficacy analysis will be performed on the mITT set, and the Clopper-Pearson method will be employed for estimating the two-sided 95% intervals of complete response rate (CRR) and overall response rate (ORR). Efficacy endpoints for survival data (e.g., duration of response [DOR], progression-free survival [PFS], and overall

	survival [OS]) will be assessed in the mITT set using the Kaplan-Meier method.<br><br>Other analyses include assessments of PK/PD, immunogenicity and cytokines. The details are provided in the Statistical Analysis Plan (SAP).<br><br>**Timing of Analysis:**<br><br>The primary analysis is planned to take place after all subjects have been followed up for at least 1 month after JWCAR029 infusion, or until death, disease progression or withdrawal from study. The final analysis will be performed after all subjects have completed the study or discontinued the study for any reason.

LIST OF ABBREVIATIONS

AE	Adverse event
AESI	Adverse event of special interest
ALC	Absolute lymphocyte count
ALL	Acute lymphoblastic leukemia
Allo-HSCT	Allogeneic hematopoietic stem cell transplant
ALT	Alanine aminotransferase
aPTT	Activated partial thromboplastin time
AST	Aspartate aminotransferase
ADA	Anti-drug antibody
AUC	Area under the curve
Auto-HSCT	Autologous hematopoietic stem cell transplant
BMA	Bone marrow aspirate
BMB	Bone marrow biopsy
BUN	Blood urea nitrogen
CAR	Chimeric antigen receptor
CBC	Complete blood count
NMPA	China Food and Drug Administration
CFR	Code of Federal Regulations
CLL	Chronic lymphocytic leukemia
Cmax	Maximum concentration
CNS	Central nervous system
CR	Complete response
CRA	Clinical research associate
CRF	Case report form
CRP	C-reactive protein
CRS	Cytokine release syndrome
CSF	Cerebrospinal fluid
CSR	Clinical study report
CT	Computed tomography
CTCAE	Common Terminology Criteria for Adverse Events
DC	Dose Confirmation
DE	Dose Expansion or Dose Escalation
DF	Dose Finding
DLBCL	Diffuse large B cell lymphoma
DLI	Donor lymphocyte infusions
DLT	Dose-limiting toxicity

DMSO	Dimethyl sulfoxide
DOR	Duration of response
DSMB	Data Safety Monitoring Board
ECG	Electrocardiogram
ECHO	Echocardiogram
ECOG	Eastern Cooperative Oncology Group
EDC	Electronic data capture
EEG	Electroencephalogram
EGF Rt	Truncated human epidermal growth factor receptor
EOS	End-of-Study
FDA	Food and Drug Administration
FDAMA	Food and Drug Modernization Act
FISH	Fluorescence in situ hybridization
flu/cy	Fludarabine and cyclophosphamide
GCP	Good Clinical Practice
GFR	Glomerular filtration rate
GM-CSF	Granulocyte macrophage colony-stimulating factor
GVHD	Graft versus host disease
HGRAC	Human Genetic Resource Administration of China
HIV	Human immunodeficiency virus
HLA	Human leukocyte antigen
HRQoL	Health-related quality of life
HSCT	Hematopoietic stem cell transplant
IB	Investigator’s brochure
IBC	Institutional Biosafety Committee
ICF	Informed consent form
ICH	International Conference on Harmonisation
ICMJE	International Committee of Medical Journal Editors
ICU	Intensive care unit
IEC	Independent Ethics Committee
IFN-γ	Interferon gamma
IgA	Immunoglobulin A
IgG	Immunoglobulin G
IgM	Immunoglobulin M
IL-5	Interleukin-5
IL-6	Interleukin-6
IL-10	Interleukin-10

IRB	Institutional Review Board
IRC	Independent Review Committee
IV	Intravenous
KM	Kaplan-Meier
LDH	Lactate dehydrogenase
LTFU	Long-term follow-up
LVEF	Left ventricular ejection fraction
mAb	Monoclonal antibody
MAS	Macrophage activation syndrome
MCL	Mantle cell lymphoma
mTPI-2	Modified Toxicity Probability Interval 2
MedDRA	Medical Dictionary for Regulatory Activities
MMSE	Mini Mental State Examination
MRD	Minimal residual disease
MRI	Magnetic resonance imaging
MTD	Maximum tolerated dose
MUGA	Multiple uptake gated acquisition
NHL	Non-Hodgkin lymphoma
NMPA	National Medical Product Administration
NIH	National Institutes of Health
NOS	Not otherwise specified
NYHA	New York Heart Association
OR	Objective response
ORR	Objective response rate
OS	Overall survival
PAS	Primary Analysis Set
PBMC	Peripheral blood mononuclear cell
PCP	Pneumocystis pneumonia
PCR	Polymerase chain reaction
P[CR]	Probability of complete response
PD	Progressive disease
P[DLT]	Probability of dose-limiting toxicity
PET	Positron emission tomography
PFS	Progression-free survival
PK	Pharmacokinetic(s)
PMBCL	Primary mediastinal B-cell lymphoma
PO	Per os (orally)
PT	Prothrombin time

qPCR	Quantitative polymerase chain reaction
RCL	Replication-competent lentivirus
RD	Recommended dose
R/R	Relapsed or refractory
SAE	Serious adverse event
SAP	Statistical Analysis Plan
SpO_2	Saturated oxygen
scFv	Single chain variable fragment
SCID	Severe combined immunodeficiency
sCRS	Severe cytokine release syndrome
SD	Stable disease
SIN	Self-inactivating
SNP	Single nucleotide polymorphism
SOC	System organ class
SRC	Safety Review Committee
tDLBCL	Transformed DLBCL from indolent histology
TEAE	Treatment-emergent adverse event
TLS	Tumor lysis syndrome
ULN	Upper limit of normal

1. INTRODUCTION

1.1 B-cell Non-Hodgkin Lymphoma

It was estimated that there were approximately 72,000 patients newly diagnosed with non-Hodgkin lymphoma (NHL) in the United States (U.S) in 2015, with about 20,000 deaths from this disease (Siegel 2015). In China, the incidence of NHL, although significantly lower than that in the U.S (Li 2012; Sun 2012; Wang 2012; Ferlay 2015), has increased over the past 20 years. According to the data published on the World Lymphoma Awareness Day Shanghai 2012, the incidence of NHL in China was 0.02‰, with 25,000 newly diagnosed cases and more than 20,000 deaths from this disease each year (http://baike.baidu.com/view/2102969.html). NHL has ranked from previously 10th as the 9th leading malignancy with respect to incidence in China. The incidence of NHL in Beijing has doubled between 1998 and 2010, increasing from 2.89 to 5.93 per 100,000 people (Yuan 2014).

In the U.S., approximately 85% of NHL cases were pathologically classified as B-cell non-Hodgkin lymphoma (B-NHL) and the other 15% of NHL cases as T/NK -cell lymphoma (NCCN 2014). The most common sub-types of aggressive B-NHL are diffuse large B-cell lymphoma (DLBCL) and mantle cell lymphoma (MCL). Research data in China showed that the sub-type DLBCL also accounted for an important proportion in Chinese patients with lymphoma, in addition to T/NL-cell lymphoma with a high proportion (24%) (Sun 2012; Li 2012). Drug resistance developed in approximately 20% of the DLBCL patients after first-line treatment with R-CHOP regimen (Coiffier 2016), and these patients had rapider disease progression and worse prognosis, and could hardly benefit from conventional therapies. Research showed that patients with primary R/R DLBCL poorly responded to salvage chemotherapy or stem cell transplantation, with a response rate of 20%, a complete response rate of only 3% and a median survival of only about 7.1 months (Telio 2012; Hitz 2015; Crump 2017). Therefore, there is an urgent need to explore promising therapeutic approaches to primary R/R DLBCL.

1.2 CD19 as a Therapeutic Target

CD19 is a 95-kDa glycoprotein present on pre-B lymphocytes until the plasma cell stage of differentiation (Stamenkovic 1988). It is a member of the immunoglobulin family and also a component of the signaling complex on the B cell surface, positively modulating the signal transduction through B cell receptors (Stamenkovic 1988, Brentjens 2011).

Expressed in most B-cell malignancies including B-NHL, CD19 has become a therapeutic target of great interest. (Li 1993, Li 1996, Davila 2012). Importantly, CD19 is not expressed by hematopoietic stem cells or other normal tissues, except for the B-cell lineages.

1.3 CD19-targeted Chimeric Antigen Receptors

CD19-specific chimeric antigen receptors (CARs) are a single-chain variable fragment (scFv), a fusion protein consisting of a binding domain and a signal transduction domain of the 4-1BB

and CD3ζ chain. Expression of the CD19-targeted CAR in autologous T cells has been achieved by means of in vitro transduction using recombinant retroviral or lentiviral vectors. Upon expression on the membrane surface of T cells, the CAR recognizes CD19-expressing lymphoma cells, thereby mediating CD19-specific tumor cell lysis, cytokine secretion, and T cell proliferation (Sadelain 2013). In clinical studies, anti-CD19 CARs have demonstrated excellent efficacy in adult and pediatric subjects with R/R B-cell acute lymphoblastic leukemia (ALL) and B-cell NHL. (Porter 2011, Davila 2014, Maude 2014, Kochenderfer 2015, Lee 2015).

1.4 Investigational Product JWCAR029

The investigational product JWCAR029, which is preserved in dimethyl sulfoxide (DMSO)-containing culture medium as frozen cell suspension consisting of autologous CD3+ T lymphocytes expressing CD19-specific chimeric antigen receptors (CARs), is supplied for intravenous infusion directly.

A CD19-specific CAR and a truncated human epidermal growth factor receptor (EGFRt) are introduced into autologous CD3+ T cells in vitro using a self-inactivating lentiviral vector. The CD19-specific CAR is a single-chain variable fragment (scFv) derived from a mouse CD19-specific monoclonal antibody (mAb; FMC63), consisting of a binding domain and a signal transduction domain of the 4-1BB and CD 3 ζ chain. EGFRt is a membrane protein expressed independently on the cell membrane surface and is used to track CAR+ T cells.

1.5 Clinical Experience with JWCAR029 and Relevant CAR+ T Cell Products

As similar with JCAR017 and JCAR014, JWCAR029 is a CAR+ T cell product specific for the CD19 antigen. However, they are similar products produced at different manufacturing sites using different manufacturing processes.

As of 25 April 2019, JWCAR029 has been used to treat 32 adult subjects with refractory non-Hodgkin lymphoma in a Phase 1 clinical trial. Of these subjects, 17 experienced CRS (including 12 subjects with Grade 1 CRS, 4 subjects with Grade 2 CRS, and 1 subject with Grade 3 CRS) and 5 experienced neurotoxicities (all Grade 1 to 2). These subjects recovered from the toxicities quickly after appropriate treatment. Laboratory abnormalities of hematology were the most common among the AEs reported in the subjects, and relevant variables all returned to normal after symptomatic treatments. Totally 29 of the 32 treated subjects were included in the efficacy analysis set (3 subjects were excluded from the efficacy analysis set due to infusion of non-standard product). In these subjects, the ORR and the CRR were 86.2% and 65.5% respectively at one month, and 69.00% and 62.121% respectively at three months.

As of December 2018, a total of 147 (Palomba 2018) and 288 (Alexandre 2018, Hay 2018, Gauthier 2018) subjects have been treated with JCAR017 and JCAR014, respectively, for indications including NHL, CLL, as well as adult and pediatric ALL. Complete response (CR) and partial response (PR) were observed in each group of patients treated with the conditioning regimen. Major safety concerns included dose-related cytokine release syndrome (CRS) and

severe neurotoxicity (Grade 3). CRS is characterized by high fever, fatigue, nausea, headache, dyspnea, tachycardia, rigors, hypotension, hypoxia, myalgia/arthralgia, and anorexia.

JCAR014 was used for treating adults with R/R CD19+ NHL in a Phase 1/2 clinical trial conducted at the Fred-Hutchinson Cancer Research Center. The doses used were 2×10^5, 2×10^6, and 2×10^7 CAR+ T cells/kg. The data showed (NCT01865617, Turtle 2018a) that objective response was noticed in 32 (57%) of the 57 evaluable NHL subjects who received flu/cy lympho-depleting chemotherapy, including complete response in 27 (48%) of the subjects. In addition, objective response was observed in 24 (51%) of the subjects with aggressive NHL, including complete response in 19 (40%) of the subjects.

Results from a clinical trial of JCAR014 in combination with ibrutinib in adults with R/R CD19+ CLL conducted at the Fred-Hutchinson Cancer Research Center (Gauthier 2018) showed that objective response was observed in 15 (62.5%) of the 24 subjects who did not receive ibrutinib and in 15 (83%) of the 18 subjects who also received ibrutinib. With regard to adverse reactions, among the subjects who received ibrutinib and those who did not receive ibrutinib, CRS of any grade was reported in 14 subjects (74%) and 22 subjects (92%), respectively, and CRS > Grade 3 was reported in 0 subjects (0%) and 6 subjects (25%), respectively. With regard to neurotoxicity, neurotoxicity of any grade was reported in 6 (32%) subjects and 10 subjects (42%) respectively, and neurotoxicity > Grade 3 was reported in 5 subjects (26%) and 7 subjects (29%) respectively.

In another Phase 1 clinical trial (PLAT-02 study; Gardner 2017) conducted at Seattle Children's Hospital, JCAR017 was used to treat relapsed CD19+ B-cell ALL in children and adolescents. The data showed that complete response (CR) without minimal residual disease (MRD) was achieved in 40 (93%) of the 43 subjects and CR without MRD in all dose groups. Of these subjects, 27 (63%) received lympho-depleting chemotherapy with cyclophosphamide alone, and 14 (33%) received lympho-depleting chemotherapy with cyclophosphamide + fludarabine. Safety data were collected from a total of 45 subjects. Of these 45 subjects, 10 (23%) experienced study drug-related serious CRS more frequently and 9 (21%) experienced Grade 3 and above neurotoxicities. The maximum tolerated dose (MTD) exceeded 1×10^7 CAR+ T cells/kg for subjects who did not receive lympho-depleting chemotherapy with fludarabine + cyclophosphamide (flu/cy) before infusion, and was 5×10^6 CAR+ T cells/kg for those subjects who received lympho-depleting chemotherapy.

The ongoing multi-center, pivotal Phase 1/2 clinical trial (TRANSCEND study; NCT02631044) investigated JCAR017 in adult relapsed/refractory large B-cell lymphoma. The data recently presented at ASH (Palomba 2018) showed that objective response and complete response were achieved in 77 (75%) and 56 (55%) of the 102 subjects, respectively, with an objective response rate of 51% and a complete response rate of 38% at three months. Separate analysis of the subjects in the core cohort (unspecified diffuse large B-cell lymphoma, transformed follicular lymphoma, advanced B-cell lymphoma, ECOG score of 0 to 1) showed an objective response rate of 59% at three months and a complete response rate of 45% at three months. Safety data

were collected from a total of 102 subjects, 38 (37%) of whom experienced CRS reactions, including 1 subject (1%) with Grade 3 or 4 CRS. Twenty-three (23%) of the subjects experienced neurotoxicities, including 13 subjects (13%) with Grade 3 or above neurotoxicities.

For more details, please refer to the Investigator's Brochure (IB) for JWCAR029.

2. JUSTIFICATION OF THE STUDY

2.1 Justification of JWCAR029 Dose

The safety of the defined doses of JWCAR029 was investigated in a Phase 1 study, in which the dose of JWCAR029 was escalated to 150 × 10^6 CAR+ T cells successfully using the modified toxicity probability interval 2 (mTPI-2) design. In addition, JCAR014 is currently being studied in adult R/R CD19+ ALL, CLL and NHL in a Phase 1/2 clinical trial conducted at the Fred Hutchinson Cancer Research Institute (NCT01865617). The doses used were 0.2 × 10^6, 2 × 10^6, and 20 × 10^6 CAR+ T cells/kg. In the ZUMA-1 clinical trial conducted by the Kite Company where the dose of anti-CD19 CAR+ T cells used for the treatment of diffuse large B-cell lymphoma (DLBCL) was 2 × 10^6 CAR+ T cells/kg, the infusion doses received by the subjects varied according to the body weight, but the therapeutic doses were within a range. Therefore, in the Phase 1-based expansion study (Phase 2 study), the subjects were randomized in a 1:1 ratio to receive infusion treatment at the recommended doses of 100 × 10^6 CAR+ T cells (dose group C) and 150 × 10^6 CAR+ T cells (dose group D).

The results of the Phase 2 study available as of 18 March 2020 showed an ORR of 66.67% (18/27) and a CRR of 59.3% (16/27) in the 100 × 10^6 dose group versus an ORR of 51.61% (16/31) and a CRR of 32.3% (10/31) in the 150 × 10^6 dose group at three months. Describing the safety in the two dose groups by the 150 × 10^6 dose group (n=32) versus the 100 × 10^6 dose group (n=27), there were 5 and 2 deaths, as well as 6 and 5 subjects with SAEs respectively after JWCAR029 infusion. Specifically, there were 3 and 0 deaths as well as 5 and 2 subjects experiencing SAEs within 30 days after infusion, respectively; there were no deaths in either group and 1 and 2 subjects with SAEs within 31 to 90 days after infusion, respectively; there were 2 deaths in each group as well as 0 and 1 subject with SAEs beyond 91 days after infusion, respectively. As regard to AE of special interest (AESI), there were 15 and 13 subjects experiencing CRS in the two groups, respectively, including 3 subjects experiencing ≥ Grade 3 CRS (2 subjects with Grade 3 CRS and 1 subject with Grade 4 CRS) in the 150 × 10^6 dose group; there were 9 and 3 subjects experiencing neurotoxicity (NT) in the two groups, respectively, including 2 subjects with Grade 3 NT in the 150 × 10^6 dose group, and 0 subjects with ≥ Grade 4 NT. Based on the above, the 100 × 10^6 dose group demonstrated better efficacy and safety data than the 150 × 10^6 dose group in the JWCAR029-002 study. Descriptive analyses of baseline status before JWCAR029 infusion revealed differences in some parameters of demographics, baseline characteristics, medical history and co-morbidities, as well as safety during lympho-depletion between the two dose groups of JWCAR029-002, with the subjects in the 150 × 10^6 dose group having poorer results. Better efficacy and safety were

observed for the 100 $\times 10^6$ dose group versus the 150 $\times 10^6$ dose group in the clinical study, which was probably related to these differences in the baseline status. Based on the above study results of efficacy and safety and meanwhile, as the volume of CAR-T cell infusion needs to be calculated based on the concentration and dose of CAR-T cells in the solution for infusion, the fixed dose of 100 $\times$ 10^6 is recommended as the clinical dose considering the feasibility and convenience of clinical operations.

2.2 Justification of Lympho-depleting Chemotherapy Regimen

Although clinical studies have reported that approximately 40 NHL subjects were treated with 3 different CD19-targeted CAR+ T cell products, no optimal lympho-depleting chemotherapy regimen before CAR+ T cell treatment was formed in any of these clinical studies (Gardner 2016; Turtle 2016a).

When the lympho-depleting chemotherapy with fludarabine + cyclophosphamide (flu/cy) was compared with that using cyclophosphamide alone, the combination chemotherapy significantly prolonged the proliferation and persistence of CAR+ T cells of defined CD4+ and CD8+ composition, probably through the mechanism that the combination chemotherapy depleted the lymphocytes more thoroughly to improve the micro-environment, increase the levels of cytokines promoting the proliferation and survival of CAR+ T cells, and delay or prevent the occurrence of CAR immunoreaction. Further, the combination chemotherapy also resulted in better CRR and progression-free survival (PFS) in the subjects than the lympho-depleting chemotherapy with cyclophosphamide alone (Turtle 2016).

The trial data presented at the American Society of Hematology (ASH) Annual Meeting 2014 (Turtle 2014) indicate that the in vivo expansion, persistence and anti-tumor activity of CAR+ T cells were less desirable and JCAR014 failed to exert significant therapeutic effects in subjects who previously received non-flu/cy lympho-depleting chemotherapy. The in vivo expansion and anti-tumor activity of JCAR014 improved after 3 to 5 days of lympho-depleting chemotherapy with high doses of cyclophosphamide (60 mg/kg) and fludarabine (25 mg/m^2/day). However, high-dose (2 $\times$ 10^7 CAR+ T cells/kg; please see the IB of JWCAR029 for more details) cell therapy will lead to cardiac toxicities, neurotoxicities and death. Another clinical study showed significant anti-tumor activity of CAR+ T cells in 11 treated NHL subjects after lympho-depleting chemotherapy with high doses of cyclophosphamide (60-120 mg/kg) and fludarabine (25 mg/m^2/day $\times$ 5 days) (Kochenderfer 2015). However, significant cardiac toxicities, neurotoxicities and even deaths were observed in the subjects when CAR+ T cells were infused at the highest dose level (5 $\times$ 10^6 CAR+ T cells/kg). When low-dose CAR+ T cells (1 $\times$ 10^6 CAR+ T cells/kg) were infused following lympho-depleting chemotherapy with low doses of cyclophosphamide (300 mg/m^2/day $\times$ 3 days) and fludarabine (30 mg/m^2/day $\times$ 3 days), significant anti-tumor activity was observed without serious cardiac toxicity or neurotoxicity, although significant transient neurotoxitities (aphasia and ataxia) were still noticed (Kochenderfer 2014a). These results suggest that compared with the high-dose lympho-depleting chemotherapy regimen, the combination regimen of low-dose

cyclophosphamide and fludarabine still preserved anti-tumor activity with relatively less toxicities.

Therefore, a lympho-depleting chemotherapy regimen of low-dose cyclophosphamide (250 mg/m^2/day × 3 days) and fludarabine (25 mg/m^2/day × 3 days) will be used in this study.

2.3 Justification of Study Design

The doses of 100 × 10^6 CAR+ T cells and 150 × 10^6 CAR+ T cells were finally selected as the recommended phase 2 doses from a Phase 1 dose-finding study in subjects with R/R DLBCL, and the two dose levels were used for the Phase 1-based expansion study (Phase 2 efficacy study) in R/R DLBCL patients. Based on the results of the Phase 2 efficacy study, the dose of 100 × 10^6 CAR+ T cells will be used to treat subjects with primary R/R DLBCL in this study to evaluate the safety of the dose. As it is not feasible to use the method of blinding for JWCAR029 cell therapy, this study will be open-label.

Summary of Risk/Benefit Assessment:

As described in Section 1.1, the population to be enrolled in this study have an overall poor prognosis. Based on the data presented in Section 1.5, the results were favorable when other CD19-targeted CAR+ T cell therapies were used for treating advanced NHL. As noted in Section 1.5, the risks associated with treatment by other products similar to JWCAR029 included cytokine release syndrome (CRS), which was attributed to high doses of CAR+T cells in some studies and characterized by high fever, fatigue, nausea, headache, dyspnea, tachycardia, rigors, hypotension, hypoxia, myalgia/arthralgia and anorexia, severe (≥ Grade 3) neurotoxicities, and hematology abnormalities, including neutropenia, thrombocytopenia and anemia. The potential risks are described in detail in Section 7 and the IB. As of date, the above toxicities identified in the clinical studies of other CAR+ T cell products have been effectively managed. Therefore, lymphodepletion plus subsequent JWCAR029 therapy may be an important treatment option for subjects with R/R B-NHL.

3. STUDY OBJECTIVES AND ENDPOINTS

The objectives and endpoints of this study are provided in Table 1.

Table 1: Objectives and Endpoints of the Study

**Study Objectives**	Primary:<br>• To evaluate the safety of JWCAR029 in subjects with primary R/R DLBCL<br>Secondary:<br>• To evaluate the anti-tumor activity of JWCAR029<br>• To evaluate the durability of anti-tumor activity of JWCAR029<br>• To describe the pharmacokinetic (PK) and pharmacodynamic (PD) characteristics of JWCAR029<br>• To evaluate the immunogenicity of JWCAR029<br>• To evaluate the impact of the properties of JWCAR029 on safety, PK, and anti-tumor activity
**Study Endpoints**	Primary:<br>• The type, frequency and severity of adverse events (AEs) and laboratory abnormalities<br>Secondary:<br>• One-month complete response rate (CRR) and overall response rate (ORR) as assessed by the investigator<br>• Best objective response rate (BORR): defined as the best response achieved during the period from start of treatment to disease progression/relapse or to the start of another anticancer therapy<br>• 3-month complete response rate (CRR) as assessed by the investigator<br>• 3-month objective response rate (ORR=CR+PR) as assessed by the investigator<br>• Duration of response (DOR), which is defined as the length of time from the first documented response (CR or PR) to the earliest PD or death<br>• Duration of complete response (DoCR), which is defined as the length of time from the first documented CR to the earliest PD or death<br>• Time to response (TTR), which is defined as the length of time from JWCAR029 infusion to the first documented CR or PR<br>• Time to complete response (TTCR), which is defined as the length of time from JWCAR029 infusion to the first documented CR<br>• Maximum plasma concentration (C_{max}), time to maximum concentration (T_{max}), area under the plasma concentration-time curve (AUC) and other relevant PK parameters of JWCAR029 in blood<br>• Progression-free survival (PFS)<br>• Overall survival (OS)<br>• Changes in the levels of inflammatory markers (C-reactive protein, serum ferritin) associated with CRS<br>• Detection of anti-drug antibodies (ADA) to JWCAR029<br>• The number and subsets of T cells and the changes in the levels of cytokines in the serum<br>• Characterization of JWCAR029 (T-cell subsets and immunophenotype before and after treatment)<br>CD19 expression as detected by tumor biopsy

4. STUDY DESIGN AND PLAN

4.1 Overview of Study Design

This is a Phase 1 open-label, single-arm, multi-center study to investigate the safety of the JWCAR029 product containing 100 × 10^6 CAR+ T cells in adult subjects with primary R/R DLBCL. A summary of this study is provided in Table 2. In this study, the safety, efficacy and PK/PD characteristics of JWCAR029 will be assessed in all subjects.

Table 2: Study Summary

Population	Design and Sample Size	Treatment	Outcome
Subjects with primary R/R DLBCL who failed to achieve complete response (CR) after first-line treatment using anthracyclines and rituximab other CD20-targeted drugs	A Phase 1, open-label, single-arm, multi-center study, designed to investigate the safety of JWCAR029 in subjects with primary R/R DLBCL, in which the subjects will receive JWCAR029 infusion at a dose of 100 × 10^6 CAR+ T cells. After infusion, safety and other follow-up data will be collected continuously, followed by two years of follow-up after the end of treatment.<br>It is planned to enroll 12 subjects. Considering the possibility that the subjects may receive non-standard therapies and be replaced during the course of the study, the number of subjects actually enrolled may exceed 12.	JWCAR029 is infused at a dose of 100 × 10^6 CAR+ T cells.	Assess the safety

4.2 Study Procedures

Provided in the Figure below are the study procedures for the subjects

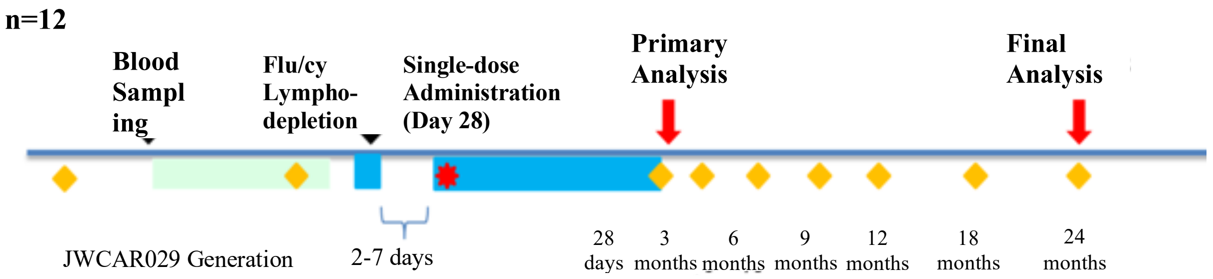

Primary study endpoints: The type, frequency and severity of adverse events (AEs) and laboratory abnormalities

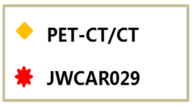

Secondary study endpoints: CR, ORR and other efficacy endpoints; PK/PD, etc.

Figure 1: Schematic Diagram of Clinical Trial Design

The pre-treatment period includes screening, leukapheresis and pre-treatment assessment. The pre-treatment period begins with assessment as to whether the subject meets the inclusion and exclusion, and leukapheresis and pre-treatment assessment will be performed immediately after enrollment of eligible subjects. The purpose of leukapheresis is to obtain a sufficient quantity of peripheral blood mononuclear cells (PBMCs) for the generation of the JWCAR029 product.

Pre-treatment assessment includes bone marrow aspiration/biopsy (BMA/BMB) and/or tumor biopsy (for subjects with detectable disease and as deemed feasible by the investigator) prior to treatment (i.e., lympho-depleting chemotherapy and JWCAR029 therapy).

There exists a possibility that a subject may receive bridging anticancer therapy while waiting for JWCAR029 generation. Provided bridging anticancer therapy is administered a subject, baseline assessment must be performed for the subject again to ensure that the subject meets all inclusion/exclusion criteria and can be included in the study. If the first leukapheresis does not generate JWCAR029 for a subject successfully, additional leukapheresis may be performed after discussion with the Sponsor. After JWCAR029 is generated successfully, the subject will enter the treatment period and receive JWCAR029 treatment.

The treatment period consists of a lympho-depleting chemotherapy period and a JWCAR029 infusion period. Lympho-depleting chemotherapy will be administered as a combination chemotherapy (flu/cy) with fludarabine (25 mg/m^2/day × 3 days) and cyclophosphamid (250 mg/m^2/day × 3 days), and a single intravenous infusion of JWCAR029 will be given 2 to 7 days after lympho-depleting chemotherapy.

After JWCAR029 infusion, safety assessment will be performed, including assessment of adverse events and laboratory abnormalities. All adverse events (AEs) and serious adverse events (SAEs) occurring during the treatment period will be recorded. Toxicities will be graded according to the National Cancer Institute (NCI) Common Terminology Criteria for Adverse Events (CTCAE) version 5.0, except for cytokine release syndrome (CRS), which is graded based on the modified criteria system (Davila 2014, Lee 2014, Neelapu 2017).

After JWCAR029 treatment, the subjects will be followed up for two years to collect information about safety, efficacy, PK/PD, disease status, additional anticancer therapy, survival, etc. Follow-up of safety and survival will also continue for the subjects even after disease progression or start of any additional anti-cancer therapy.

4.3 Duration of Study and Estimated Duration of Participation

Enrollment of 12 subjects is expected to continue for 9 to 12 months and follow-up, starting after JWCAR029 infusion, is expected to continue for 24 months for each subject. Therefore, the duration of the study is estimated to be 33 to 36 months.

4.4 Study Oversight

4.4.1 Safety Review Committee

The Safety Review Committee (SRC), formed by the principal investigator(s) of the clinical trial and the Biomedical and Pharmacovigilance (PV) staff of Shanghai Mingju Biotechnology Co., Ltd, is responsible for reviewing the oversight, safety assessment and protocol implementation of the clinical trial. The SRC will observe and evaluate the safety of the subjects continuously during the study and make a decision as to whether the study should be continued (including continuing with the dose, reducing the dose, or discontinuing the study,

etc.).

4.5 Protocol Product Deviation Plan

The Protocol Product Deviation Plan (PPDP) of Shanghai Mingju Biotechnology Co., Ltd defines the evaluation and decision-making procedures for subjects receiving non-standardized investigational product (non-standard JWCAR029), which mainly refers to products with all the safety parameters (including sterility, endotoxin, mycoplasma, vector copy number and replication-competent lentivirus) meeting the release specification and only one quality attribute indicator failing to meet the release specification. In this management process, a consensus must be reached between the principal investigator and the medical monitor of Shanghai Mingju Biotechnology Co., Ltd that the well-being of the subjects and the risks/benefits of the investigational product are acceptable. However, the Quality Control (QC) Manager will conduct an assessment of this consensus and finally decides on how to dispose of the batches of the investigational product.

4.6 Additional Treatment Cycles and Re-treatment

An additional treatment cycle or re-treatment will not be considered for lymphoma subjects responding to JWCAR029 who fail to achieve complete response (CR) after completion of one treatment cycle.

5. STUDY POPULATION

The target study population consists of subjects with primary R/R DLBCL, who should meet the following inclusion and exclusion criteria at screening and if appropriate, will be assessed against these criteria again before lympho-depleting chemotherapy.

5.1 Inclusion Criteria

The subjects must meet all of the following criteria to be included in this study:

1. Aged ≥ 18 years
2. Having signed the Informed Consent Form (ICF)
3. Patients with primary refractory DLBCL who failed to achieve complete response (CR) after first-line treatment using anthracyclines and rituximab other CD20-targeted drugs
   a. Stable disease (SD) after minimum 3 cycles of first-line treatment, or
   b. Biopsy-proven residual disease despite partial response (PR) after 6 cycles of first-line treatment, or
   c. Progressive disease (PD) on any course of first-line treatment
4. PET-positive lesions established according to the Lugano criteria
5. An Eastern Cooperative Oncology Group (ECOG) performance status (PS) score of 0 or 1
6. Adequate organ function:
   a. Adequate bone marrow function as assessed by the investigator to receive lympho-depleting chemotherapy [absolute neutrophil count (ANC) ≥ 1000/μL, platelet count ≥

75,000/μL, and absolute lymphocyte count ≥ 100/μL]

b. Serum creatinine ≤ 1.5 × upper limit of normal (ULN) or creatinine clearance (Cockcroft and Gault) > 50 mL/min (excluding serum creatinine clearance decreased due to compression from lymphoma mass)
c. Alanine aminotransferase (ALT) ≤ 5 × ULN and total bilirubin < 2.0 mg/dL (or < 3.0 mg/dL for subjects with Gilbert's syndrome or lymphoma involving the liver)
d. Pulmonary function: dyspnea ≤ CTCAE grade 1 and SpO_2 ≥ 92% in room air
e. Cardiac function: left ventricular ejection fraction (LVEF) ≥ 50% shown by echocardiogram (ECHO) or multi-gated radionuclide angiography (MUGA) within 1 month after enrollment

7. Vascular access adequate for leukapheresis
8. For subjects previously treated with CD19-targeted therapy, biopsy-proven CD19 expression in the lymphoma lesions
9. Women of childbearing potential (all women who can get pregnant physiologically) must agree to use highly effective methods of birth control for one year after JWCAR029 infusion
10. Male subjects with partners of childbearing potential must agree to use effective barrier methods of birth control for one year after the last JWCAR029 infusion

5.2 Exclusion Criteria

Subjects meeting any of the following criteria will be excluded from this study:

1. DLBCL patients who were treated by second-line and late therapies
2. Patients with lymphoma who are known to be negative for CD19 expression
3. Primary central nervous system lymphoma (subjects with secondary CNS lymphoma are allowed to be enrolled)
4. History of other malignancies that have not achieved complete response for at least 2 years (this 2-year limitation may be exempted for the following conditions: non-melanoma skin cancer, completely resected stage I tumors with a low probability of recurrence, limited-stage prostate cancer post-treatment, and biopsy-confirmed cervical carcinoma in situ, or squamous intraepithelial lesions evidenced by PAP smears)
5. Presence of the following at screening:
   a. Positivity for hepatitis B surface antigene (HBsAg) (regardless of whether there is an increase in the copies of hepatitis B virus DNA)
   b. Positivity for hepatitis B core antibody (HBcAb) with an increase in the copies of hepatitis B virus DNA
   c. Infection with hepatitis C virus, human immunodeficiency virus (HIV) or syphilis
6. Deep vein thrombosis (DVT) (cancer embolism or thrombus) or pulmonary embolism (PE)

within 3 months before the signing of the Informed Consent Form

7. Ongoing anticoagulant therapy for deep vein thrombosis (DVT) or pulmonary embolism (PE) within 3 months before the signing of the Informed Consent Form
8. Uncontrolled systemic fungal, bacterial, viral or other infections
9. Acute or chronic graft-versus-host disease (GVHD)
10. History of any cardiovascular disease within the past 6 months: New York Heart Association (NYHA) Class III/IV heart failure, cardiac angioplasty or stenting , myocardial infarction, unstable angina, or other clinically significant heart disease
11. History or current evidence of clinically significant CNS diseases at screening, such as epilepsy, epileptic seizures, paralysis, aphasia, strokes, severe brain injury, dementia, Parkinson's disease, cerebellar disease, organic brain syndrome (OBS) or mental disorders
12. Pregnant or lactating women. Women of childbearing potential must have a negative result of serum pregnancy test within 48 hours before start of lympho-depleting chemotherapy
13. Use of any of the following medications or therapies within the specified time before leukapheresis:
    a. Alemtuzumab within 6 months before leukapheresis
    b. Cladribine within 3 months before leukapheresis
    c. Fludarabine within 3 months before leukapheresis
    d. Anti-CD20 monoclonal antibodies within 7 days before leukapheresis
    e. Venetoclax within 4 days before leukapheresis
    f. Idelalisib within 2 days before leukapheresis
    g. Lenalidomide within 1 day before leukapheresis
    h. Corticosteroids at therapeutic doses (defined as prednisone or equivalent > 20 mg/day) within 7 days before leukapheresis or within 72 hours before administration of JWCAR029. However, physiological replacement and topical/inhaled steroids are allowed
    i. Low-dose chemotherapy (such as vincristine, rituximab, cyclophosphamide ≤ 300mg/m^2), if required to control the patient's condition after leukapheresis,must have stopped for ≥ 7 days before the lympho-depleting chemotherapy
    j. Non-lymphocytotoxic chemotherapeutic agents (e.g., doxorubicin, vincristine, gemcitabine, oxaliplatin, carboplatin, etoposide) within 1 week before leukapheresis. Use of oral chemotherapeutics (including lenalidomide and ibrutinib) before leukapheresis does not preclude enrollment if it has been > 3 half-lives
    k. Use of lymphocytotoxic chemotherapeutics (such as cyclophosphamide, ifosfamide,

bendamustine, chlorambucil, melphalan) within 2 weeks before leukapheresis

l. Use of an investigational product within 4 weeks before leukapheresis. However, this criterion does not preclude enrollment if the subject did not respond to or progressed on the investigational treatment and it has been > 3 half-lives before leukapheresis

m. Therapies for the treatment of GVHD within 4 weeks before leukapheresis and JWCAR029 infusion, such as calcineurin inhibitor, methotrexate or other chemotherapeutics, mycophenolate mofetil, rapamycin, thalidomide, and immunosuppressants like anti-TNF, anti-IL-6 or anti-IL-6R antibodies)

n. Donor lymphocyte infusion (DLI) within 6 weeks before administration of JWCAR029

o. Chemotherapy within 6 weeks before leukapheresis, involving multiple lesions or large storage areas for bone marrow, such as the sternum or pelvis. In this case, the subject is eligible for enrollment if PD occurred at the site of radiotherapy or presence of PET-positive lesions at other unirradiated sites. Radiotherapy for a single lesion is allowed within 2 weeks before leukapheresis if PET-positive lesions are present at other unirradiated sites

p. Allogeneic hematopoietic stem cell transplantation (allo-HSCT) within 90 days before leukapheresis

14. Presence of any factors that would affect the subject's compliance with the protocol in the investigator's judgment, including uncontrollable medical, psychological, family, sociological or geographical factors; or unwilling or inability to follow the procedures required in the study protocol

15. Previous treatment with CAR+T cells or other genetically modified T cells

5.3 Childbearing Potential and Contraception Requirements

A woman will be considered to be of childbearing potential if any of the following criteria is not met:

- Postmenopausal for at least 12 consecutive months (i.e., no menstrual periods), or
- Surgical sterilized (including hysterectomy, salpingectomy, or bilateral oophorectomy; tubal ligation is not considered surgical sterilization)

Female subjects of childbearing potential must have a negative serum pregnancy test at screening and within 48 hours before the start of the study treatment.

Female subjects of childbearing potential who are sexually active must agree to use a highly effective method of birth control plus an adjunctive method of birth control through at least 28 days before start of the study treatment to one year after completion of the study treatment.

Male subjects with partners of childbearing potential must agree to use effective barrier method of birth control for 1 year after completion of the study treatment and should not donate semen or sperms throughout participation in the study.

Highly effective methods of birth control are defined as those that result in a low failure rate (i.e., less than 1% per year) when used consistently and correctly. Listed below are highly effective methods of birth control:

- Intrauterine device (IUD)
- Hormones (contraceptives, injectables, implants, patches)
- Tubal ligation
- Vasectomy of partner

The adjunctive methods of birth control (barrier) are listed as follows:

- Male condoms with spermicide
- Diaphragms or cervical caps with spermicide

5.4 Removal of Subjects from Treatment or Study

At the time of signing the Informed Consent Document (ICD), the subjects will be instructed that they are free to withdraw their informed consent to the study at any time for any reason. However, all subjects receiving JWCAR029 treatment are encouraged to continue to complete all study assessment until the end of study (EOS). If a subject has withdrawn the consent from the study or requested to stop the study treatment, Shanghai Mingju Biotechnology Co., Ltd must be notified and the reason(s) must be recorded.

5.4.1 Screen Failure

Screen failures are defined as subjects who signed the Informed Consent Document (ICD) to participate in the clinical study but were not subsequently included in the study. (See Section 8.2 for Screening). Minimum screen failure information is required to ensure transparent reporting of screen failure subjects in compliance with the requirements of Consolidated Standards of Reporting Trials (CONSORT) and to respond to queries from the regulatory authorities. Such information includes demographics, inclusion and exclusion criteria, and any serious adverse events (SAEs).

Individuals who do not meet the criteria for participation in this study (screen failure) may be re-screened.

5.4.2 Treatment Discontinuation by Subjects before Receiving the Study Treatment

For subjects who underwent apheresis but did not receive the study treatment, the EOS visit and survival follow-up should be completed.

A subject's study treatment may not occur for any of the following reasons:

- The subject did not receive the study treatment due to disease-related complications
- The subject did not receive the study treatment due to interim treatment-related toxicities (i.e., due to bridging anticancer therapy)
- The subject did not receive the study treatment because the subject no longer meets the inclusion criteria for other reasons (not related to disease or interim treatment)
- JWCAR029 could not be generated
- Other

5.4.3 Discontinuation of Further Study Treatment by Subject

In rare events, subjects should be reported as treatment discontinuation. Reasons for treatment discontinuation will include the following:

- AE
- Investigator's discretion
- Subject's decision
- Other

5.4.4 Withdrawal of Subjects from Study

Subjects may be withdrawn from the study for any of the following reasons:

- Withdrawal of informed consent by the subject
- Termination of the study by the Sponsor
- Lost to follow-up
- Death
- Other

5.4.5 Replacement of Subjects

Subjects enrolled in this study who did not receive JWCAR029 treatment or received a non-standard product will be replaced to ensure that a minimum of 12 subjects will receive standard JWCAR029. In this case, the subject number will not be re-used. Subjects receiving a non-standard product will be followed up as per the protocol.

6 STUDY TREATMENT

6.1 Leukapheresis

Leukapheresis will be performed for each subject upon enrollment into the study to obtain a sufficient quantity of peripheral blood mononuclear cells (PBMCs) for the generation of the investigational product JWCAR029. If the generated JWCAR029 product cannot be used for infusion due to technical problems arising during the procedure or during product processing,

a second leukapheresis procedure may be performed for the subject without needing to re-sign the ICD.

6.2 Anticancer Therapy During the Period of Leukapheresis and Lympho-depleting Chemotherapy

During the period of JWCAR029 preparation (i.e., after leukapheresis but before lympho-depleting chemotherapy), anticancer therapy, if required, is allowed to control the disease. Low-dose chemotherapy (including but not limited to vincristine, rituximab, cyclophosphamide ≤ 300 mg/m^2) must be completed 7 days before lympho-depleting chemotherapy. If PET-positive lymphoma lesions unirradiated are present, local irradiation therapy for the treatment of single lesions or sub-lesions is allowed. If anticancer therapy is required during this period, PET-CT scans and other pre-treatment assessments must be performed after completion of anticancer therapy.

6.3 Lympho-depleting Chemotherapy

The subjects will undergo three days of lympho-depleting chemotherapy with fludarabine (25 mg/m^2/day) and cyclophosphamide (250 mg/m2/day) before JWCAR029 treatment. For detailed dosing information about these drugs, please refer to the latest package inserts for further details on the management of the drugs, including recommendations on dose modifications possibly required for organ dysfunction. Please discuss the dose reduction program with the Sponsor's medical monitor.

Lymphodepleting chemotherapy must be completed within 2 to 7 days prior to administration of JWCAR029.

Serum creatinine test should be performed within 48 hours prior to lympho-depleting chemotherapy. If serum creatinine is > 1.5 × upper limit of normal (ULN), or creatinine clearance (calculated by Cockcroft-Gault formula; see Appendix C) is ≤ 50 mL/min, the necessity of postponing the lympho-depleting chemotherapy should be determined after communication with the Sponsor. If a delay of more than 14 days in lympho-depleting chemotherapy, it should also be discussed with the medical monitor.

As a routine practice, antiemetic therapy may be administered prior to lympho-depleting chemotherapy. Mesna (Sodium 2-mercaptoethane sulfonate) may be used for subjects with a history of hemorrhagic cystitis.

6.4 Study Treatment: JWCAR029

The investigational product JWCAR029, which is preserved in dimethyl sulfoxide (DMSO)-containing culture medium as frozen cell suspension consisting of autologous T lymphocytes expressing CD19-specific chimeric antigen receptors (CARs), is supplied for intravenous infusion directly.

A CD19-specific CAR and a truncated human epidermal growth factor receptor (EGFRt) are introduced into autologous T cells in vitro using a self-inactivating lentiviral vector. The CD19-

specific CAR is a single-chain variable fragment (scFv) derived from a mouse CD19-specific monoclonal antibody (mAb; FMC63), consisting of a binding domain and a signal transduction domain of the 4-1BB and CD 3 ζ chain. EGFRt is a membrane protein expressed independently on the cell membrane surface and is used to track CAR+ T cells.

For more details about packaging and labeling, product requirements and shipment, product preparation and administration, as well as product disposal and destruction, please refer to JWCAR029 Product Manual.

6.4.1 Dose and Dosing Regimen

In this clinical trial of JWCAR029, The dose of 100×10^6 CAR+ T cells is used to investigate its safety in subjects with refractory diffuse large B-cell lymphoma (DLBCL). This dose is equivalent to the dose of 1.0×10^6 CAR+ T cells/kg for adults weighing 100 kg or the dose of 2×10^6 CAR+ T cells/kg for adults weighing 50 kg. Dose escalation will not be considered in this study. During the course of the study, the SRC will perform continuous monitoring and evaluation of safety in the subjects and decides as to how the study will be continued depending on the safety results.

6.4.2 Other Treatments Prior to Administration of JWCAR029

It is recommended that the subjects be given acetaminophen 450-650 mg orally and diphenhydramine hydrochloride 20-50 mg (PO or IV) 30 to 60 minutes prior to administration of JWCAR029 These drugs may be administered prophylactically or replaced with other drugs of the same class before infusion of the investigational product at the investigator's discretion on a case-by-case basis. Based on the investigator's assessment of the symptoms, these drugs may be administered repeatedly as needed. Pre-intervention with steroids should be avoided.

6.4.3 JWCAR029 Preparation and Cell Recovery

Details are provided in the JWCAR029 Product Manual. JWCAR029 must be thawed before use and the labeled volume should be administered to the subject in no later than 2 hours after removal of the product from the shipping container. If JWCAR029 is kept outside the shipping container for more than 2 hours, the product should be detained and the study team of Shanghai Mingju Biotechnology Co., Ltd should be notified immediately.

6.4.4 Administration of JWCAR029

All eligible subjects should be admitted to the hospital to receive the investigational product.

The subjects must be placed under continuous monitoring while receiving intravenous (IV) infusion of JWCAR029. Vital signs (temperature, respiratory rate, heart rate, blood pressure, and SpO_2 by fingertip pulse oximeter) should be monitored before (within 15 minutes), during and after (within 15 minutes) each IV infusion. The monitoring is performed every 15 minutes within the hour after infusion and then every one hour within the two hours after infusion. If a subject is found of unstable vital signs 4 hours after IV infusion, the vital signs should be monitored as clinically indicated until they become stable.

CAR+ T cells must be administered intravenously to the subjects within the specified timeline after thawing upon receipt of JWCAR029. If JWCAR029 is shipped beyond the specified timeline, the product should be discontinued from use and Shanghai Mingju Biotechnology Co., Ltd should be notified immediately.

For complete information, please refer to JWCAR029 Product Manual.

6.4.5 Use of Non-standard Products

The generated product, if failure to meet the non-safety attribute specification in the release specification, may also be considered for infusion, but it must be based on a comprehensive evaluation of the subject's well-being and the risks/benefits to the subject by the principal investigator of the clinical study site and the medical monitor of Shanghai Mingju Biotechnology Co., Ltd.

Any subject receiving a non-standard product will be replaced but will also be followed up as per the protocol.

6.4.6 Concomitant Medications

During this clinical trial, all subjects must not use illegal drugs, Chinese herbal medicines, prescription/OTC drugs not approved by the investigator, or excessive alcohol.

Table 3 summarizes the reporting periods for concomitant medications in subjects treated with JWCAR029.

Table 3: Reporting Periods for Concomitant Medications in Subjects Treated with JWCAR029

Reporting periods	What to Be Recorded/Reported
From preliminary informed consent to 90 days after administration of JWCAR029 or to the EOS visit, whichever is earlier	All medications used must be recorded/reported
From 91 days after administration of JWCAR029 to the EOS visit	The following medications must be recorded/reported:<br>• Corticosteroids<br>• Drugs used to treat GVHD<br>• Anticancer therapy<br>• Drugs used to treat AE/SAE related to JWCAR029

Given that a large amount of data will be generated during the inpatient stay period, a simplified method will be used in the Case Report Form (CRF) to collect concomitant medications. Therefore, during the inpatient and ICU stay periods, the following medications should not be entered into the CRF "Concomitant Medications" Page:

- Intravenous fluids (except for intravenous fluids used to treat CRS-related hypotension, in which case they should be recorded)
- Heparin flushes

- Stool softeners
- Vitamins, minerals, health supplements
- Saline flushes
- Lotions

During the inpatient and ICU stay periods, the following therapies used should be reported:

- Vasopressors
- Oxygen inhalation

6.5 Prohibited Medications

The following medications are prohibited during the clinical trial, unless no response to the study treatment or they are used for subsequent treatment of lymphoma or one year after treatment with JWCAR029:

- Steroids: Corticosteroids (> 20 mg/day prednisone or equivalent) should not be used, unless for the purpose of treating severe CRS (sCRS). Therapeutic doses of steroids can be used only when the subject is critically ill or in other conditions necessitating their use, or JWCAR029 cells are no longer detectable. Where steroids must be used in other conditions that necessitate their use, discussion must be held with Shanghai Mingju Biotechnology Co., Ltd before use (e.g., intravenous immunoglobulin). Concomitant steroids are not allowed prior to JWCAR029 infusion. Steroids for physiological replacement are allowed (hydrocortisone ≤ 12 mg/m^2/day or equivalent [prednisone ≤ 3 mg/m^2/day or dexamethasone ≤ 0.45 mg/m^2/day]). For prevention of CNS recurrence, topical, inhaled, and intrathecal steroids are allowed.

The following drugs are prohibited during the treatment and follow-up periods, unless they are used as anti-neoplastic agents after failure to respond to JWCAR029 or progression of lymphoma:

- DLI
- Therapies for treatment of GVHD (e.g., calcineurin inhibitors, methotrexate or other chemotherapeutics, mycophenolate mofetil, rapamycin, thalidomide, immunosuppressants like anti-TNF, anti-IL-6, or anti-IL-6R antibodies)
- Anti-neoplastic agents, excluding those required for lympho-depleting chemotherapy and in the event of JWCAR029 failure or sCRS
- Cetuximab or other anti-EGFR therapies, except in the event of JWCAR029 failure or sCRS
- Investigational products

- Radiotherapy, except for locally controlled single lesions and PET-positive lesions at other sites

In addition, given the potential effects of colony-stimulating factors (CSFs) on immune cell subsets and their functions and on CAR+ T cells, as well as their potential promoting effect on cytokine release, use of granulocyte colony-stimulating factor (G-CSFs) for enrolled subjects before leukapheresis and before or after JWCAR029 infusion should be weighed carefully against the risks and benefits by the investigator based on the clinical conditions of individual patients. In particular, use of granulocyte macrophage colony-stimulating factor (GM-CSF) is not recommended.

If immunotherapy with anti-PD-1/PD-L1 antibodies is used before JWCAR029 infusion, there must be an interval of 4 to 6 weeks from the last dose of immunotherapy before JWCAR029 infusion.

6.6 Recommendations on Care Support, Other Therapies and Monitoring

It is strongly recommended that prophylactic treatments be administered to subjects at a risk of tumor lysis syndrome (TLS) according to the routine practice or clinical standards. Care support for CRS is detailed in Section 7.1.

Red blood cell and platelet transfusions are permitted according to routine practice or clinical standards. For granulocyte colony-stimulating factor (G-CSF), as described in the previous paragraph, their use should be handled with caution based on a risk/benefit assessment and the clinical conditions of the individual patients.

According to the criteria, prophylactic or empiric anti-infectives (e.g., trimethoprim/sulf amethoxazole for prophylaxis of pneumocystis pneumonia [PCP], broad-spectrum antibi otics, antifungals, or antivirals for treatment of febrile neutropenia) are allowed.

7 MANAGEMENT OF POTENTIAL RISKS AND TREATMENT TOXICITIES

Management of potential risks and treatment toxicities is summarized below. For a complete discussion of potential risks associated with JWCAR029 treatment, please refer to the Investigator's Brochure (IB).

7.1 Cytokine Release Syndrome

Intravenous infusion of the CAR+ T-cell product JWCAR029 can cause CRS. CRS is characterized by high fever, fatigue, nausea, headache, dyspnea, tachycardia, rigors, hypotension, hypoxia, myalgia/arthralgia, and anorexia. Typically, severe and life-threatening symptoms of CRS occur within days or weeks following infusion (Lee 2014). Severe CRS requires ICU care, including treatment with anti-IL-6-receptor antibody tocilizumab, steroids, antipyretics, and/or mechanical ventilation. Low-dose steroids and/or tocilizumab was reported to alleviate the symptoms of sCRS without reducing the concentration of CAR+ T cells in the blood (Davila 2014). For factors predicting a high risk of sCRS in the subjects, please refer to the IB.

It was found that elevated levels of C-reactive protein (CRP) and ferritin could serve as reliable markers of sCRS (Davila 2014, Grupp 2014, Lee 2014). Therefore, it is strongly recommended that close monitoring of CRP and ferritin levels be performed for the subjects and early prophylactic treatment be administered upon early onset of CRS and/or an elevation of CRP and/or ferritin level.

In addition, the revised CRS grading system in CTCAE better reflects CRS associated with CAR+ T cells, as detailed in Table 4.

Table 4: CRS Grading Criteria (Lee 2014)

Grade	*Symptom Description*
1: Mild	Symptoms are not life-threatening and require symptomatic treatment with antipyretics and antiemetics only (e.g., fever, nausea, fatigue, headache, myalgias, malaise)
2: Moderate	Symptoms require and respond to moderate intervention:<br>• Oxygen requirement < 40%, or<br>• Hypotension responsive to fluids or low dose of one vasopressor, or<br>• Grade 2 organ toxicity (CTCAE v5.0)
3: Severe	Symptoms require and respond to aggressive intervention:<br>• Oxygen requirement ≥ 40%, or<br>• Hypotension requiring high dose of a vasopressor (e.g., norepinephrine ≥ 20 µg/min, dopamine ≥ 10 µg/kg/min, phenylephrine ≥ 200 µg/min, or epinephrine ≥ 10 µg/min), or<br>• Hypotension requiring multiple vasopressors (e.g., vasopressin plus one of the above agents, or equivalent of norepinephrine combination vasopressor ≥ 20 µg/min), or<br>• Grade 3 organ toxicity or Grade 4 transaminitis (CTCAE v5.0)
4: Life-threatening	Life-threatening symptoms:<br>• Requirement for ventilator support, or<br>• Grade 4 organ toxicity (excluding transaminitis)
5: Fatal	Death

Detailed CRS management procedures and guidelines for the use of steroids and/or tocilizumab are presented in Figure 2. The guidelines are intended to ensure expansion of JWCAR029 cells in the body while avoiding life-threatening toxicities. These guidelines also emphasize early invention for Grade 2 CRS, or in the setting of a rapid onset or rapid progression of CRS to prevent the development of severe CRS (Grade 3 and above) and toxicities. The guidelines in Figure 2 are for reference only, and the treatment should be individualized for each subject as clinically indicated.

Grade 1 CRS

↓

- Assess for fever, perform blood culture and empiric treatment with antibiotics
- Monitor for organ dysfunction, including regular neuropsychiatric examinations
- Daily monitoring of CRP, ferritin and bleeding/clotting time
- Supportive care (e.g., antipyretics)
- If fever (> 38.6°C) > 48 hours after JWCAR029 infusion or rapid progression, or rapid elevation of CRP within 96 hours, tocilizumab 8 mg/kg IV Q24 hours × 1-2 doses and dexamethasone 10 mg BID are recommended for minimum 3 days or until CRS and the neurotoxicity are brought under control, followed by dose reduction of dexamethasone; prophylactic antiepileptic drugs are recommended
- If fever > 96 hours after infusion of CAR-T cells, particularly in the setting of rapid CRP and ferritin elevations, consider tocilizumab 8 mg/kg IV Q24 hours × 1-2 doses +/- and dexamethasone 10 mg BID for minimum 3 days or until CRS and the neurotoxicity are brought under control, followed by dose reduction of dexamethasone

↓

Grade 2 CRS with Mild Hypotension, Neurological Symptoms or Elevated CRP (≥ 10-15 mg/dL)

↓

- Hospital admission for monitoring until alleviation of fever and symptoms
- Symptomatic, respiratory and hemodynamic support
- Administer anti-epileptic drugs (e.g., levetiracetam), consider EEG monitoring
- Tocilizumab 8 mg/kg IV is recommended and can be repeated Q24h as needed
- If persistent fever and hypotension for > 96 hours and rapid progression (progressive hypotension, neurological symptoms and/or rapidly elevated CRP within 96 hours) after JWCAR029 infusion, or no improvement of symptoms (refractory) after the first dose of tocilizumab, consider dexamethasone 20 mg IV Q12h or 10 mg IV Q6h + tocilizumab 8 mg/kg IV Q24h × 1-2

If Grade 2 CRS with fever within 96 hours after JWCAR029 infusion, consider dexamethasone 20 mg IV Q12h or 10 mg IV Q6h

↓

Grade 3 CRS (sCRS) with Hemodynamic Instability, Respiratory Distress, Worsening of Neurotoxicity, or CRP ≥ 20 mg/dL

↓

- For hypotension, intravenous fluids can be administered as per management of Grade 2 CRS; if necessary, tocilizumab or siltuximab the same as for management of Grade 2 CRS; pressor if necessarily required; ICU care, echocardiography and hemodynamic monitoring; dexamethasone 10 mg IV Q6h and if refractory, increase to 20 mg IV Q6h; symptomatic treatment for fever and other symptoms
- For hypoxia, supplemental oxygen can be provided, including high-flow oxygen and non-invasive positive pressure ventilation; if necessary, tocilizumab or siltuximab plus corticosteroids and supportive care
- For symptomatic treatment of organ toxicity, administer anti-epileptic drugs (e.g., levetiracetam), and consider EEG monitoring; if necessary, tocilizumab or siltuximab plus corticosteroids and supportive care

↓

Grade 4 CRS (sCRS) with Life-Threatening Complications

↓

- Monitoring in ICU until stable disease
- Symptomatic, respiratory and hemodynamic support
- Administer anti-epileptic drugs (e.g., levetiracetam), consider EEG monitoring
- Tocilizumab 8 mg/kg IV and can be repeated Q24h as needed
- Dexamethasone 10-20 mg (or equivalent) IV Q6-12h
- If Grade 2 CRS does not recover or life-threatening neurotoxicity is observed within 48 hours after CAR-T cell infusion, high-dose corticosteroids are recommended (e.g., methylprednisolone 2 mg/kg loading dose + 2 mg/kg/day administered in four divided doses, or plus another IL-6 antagonist)
- Consider cyclophosphamide for CAR-T cell depletion

Figure 2: Approaches to CRS Management in NHL (Adapted/Modified from Lee 2014; Neelapu SS 2017)

7.2 Fever

According to routine or standard clinical practice, subjects presenting with fever (body temperature ≥ 38.5°C) should be examined for infection and given antibiotics, fluids and other supportive therapies. Neutropenic fever should be promptly investigated (e.g., blood culture, imaging for any infectious lesions) and treated according to routine or clinical standard practice.

If fever following administration of JWCAR029, possible CRS should be considered. Subjects should be closely observed for hemodynamic stability and changes in neuropsychiatric status.

7.3 Neurotoxicity

CAR+ T cell therapy is associated with neurotoxicity. Neurological symptoms may include changes in mental status, aphasia, varying degrees of loss of consciousness and epilepsy or epileptic seizures, as well as concurrent or isolated CRS (other CRS symptoms before or after). Neurological symptoms may occur 2 to 14 days after CAR+ T cell infusion and may require ICU monitoring, ventilator support, or endotracheal intubation to maintain tracheal patency in severe cases.

Prophylactic use of antiepileptic drugs (AEDs) such as levetiracetam (Keppra) should be considered first when the patient first presents with mild neurological symptoms such as somnolence, aphasia, or trance. In addition, prophylactic administration of levetiracetam prior to JWCAR029 infusion should also be considered for subjects with a history of epilepsy or other CNS disorders, or a history of CNS radiotherapy or other CNS therapy.

For subjects with persistent or worsening psychoneurologic changes or neurologic changes with CRS, additional glucocorticoid therapy is recommended, e.g., dexamethasone 10-20 mg IV every 12 to 24 hours, or at a higher dose or frequency if clinically indicated. If severe neurological changes occur, consider high-dose glucocorticoids (methylprednisolone 2 mg/kg loading dose followed by 2 mg/kg/day 4 times daily).

If a subject experiences CRS with concurrent neurotoxicity, the CRS should be managed according to the guidelines in Section 7.1.

For subjects with epilepsy or epileptic seizures, antiepileptic drugs are recommended. Continuous EEG monitoring can be performed as appropriate based on the patient's risk of toxicity and clinical condition. For multiple or recurrent seizures, multiple anti-epileptic drugs are required. ICU monitoring and tracheal protection may also be necessary.

Optimal treatments for cytotoxicities of CAR+ T cell are still under exploration. Treatment should be determined at the discretion of the investigator or treating physician and/or neurology consultant based on clinical routine or clinical practice. Predisposing factors for neurological dysfunction, such as infection or bleeding, should be ruled out.

There was also a hypothesis that tocilizumab for treatment of simple neurotoxicity did not promote recovery of the patients. Tocilizumab is an interleukin-6 receptor antagonist that can aggravate neurotoxicity over a short time due to transient increase in serum interleukin-6 level.

Although it was just a hypothesis that tocilizumab could worsen the risk of neurotoxicity, lack of efficacy of tocilizumab in the central nervous system (CNS) may be possibly because it cannot penetrate the blood-brain barrier. In the setting of severe neurotoxicity and CRS, other interleukin-6 blockers should be considered.

The management principles for neurotoxicity are provided in Figure 3 below.

Grade 1	Grade 2
• Close supportive care, inhalation precautions, fluids.	• Supportive care, as well as neurological examination as per Grade 1.
• Interruption of oral medications, food and water. Assess for swallowing function.	• If neurotoxicity is complicated by cytokine release syndrome (CRS), consider IV tocilizumab (tocilizumab*, 8 mg/kg) or siltuximab 11 mg/kg.
• If swallowing impairment, consider IV medications and/or nutritional supplements.	• If neurotoxicity is not complicated by CRS or in patients who did not respond to IL-6 antagonists, consider IV dexamethasone 10 mg Q6h or methylprednisolone 1 mg/kg Q12h.
• Avoid use of drugs that depress the central nervous system.	• If neurotoxicity is complicated by ≥ Grade 2 CRS, consider transfer to the Intensive Care Unit (ICU).
• For anxious patients, low-dose lorazepam (0.25-0.5 mg IV drip, Q8h) or haloperidol (0.5 mg IV drip, Q6h) can be used. And monitor the patient closely.	
• Neurology consultation.	
• funduscopy for papilledema.	
• Head magnetic resonance imaging (MRI); lumbar puncture for measurement of cerebrospinal fluid pressure; for patients with focal cranial neurological disorders, perform spinal MRI; if head MRI is impossible, perform CT scans.	
• EEG monitoring for 30 minutes daily, until resolution of the toxicity symptoms. If epilepsy is not detected by EEG, continue levetiracetam 750 mg every 12 hours.	
• If non-convulsive status epilepticus as shown by EEG, follow the management approach for status epilepticus in the CAR-T cell therapy.	
• If neurotoxicity accompanied by CRS, consider intravenous tocilizumab (tocilizumab, 8 mg/kg) or siltuximab 11 mg/kg.	All the doses are for adult patients<br>* The maximum dose of tocilizumab per injection is 800 mg

Grade 3	Grade 4
• Supportive care, as well as neurological examination as per Grade 1.	• Supportive care, as well as neurological examination as per Grade 1.
• Transfer to the ICU is recommended.	• ICU monitoring. Consider mechanical ventilation to keep airways clear.
• If neurotoxicity is accompanied by CRS and IL-6 receptor antagonists were not used in previous therapy, IL-6 receptor antagonist therapy can be used as per Grade 2 neurotoxicity.	• Administer treatment with IL-6 receptor antagonist and repeat the neuroimaging as described for Grade 3 neurotoxicity.

• If neurotoxicity without CRS, or non-responsive to IL-6 receptor antagonist therapy, use corticosteroids as per Grade 2 neurotoxicity, continued until the symptom has resolved to Grade 1, followed by tapering.	• Continue high-dose corticosteroids until symptoms resolve to Grade 1, followed by tapering. For example: methylprednisolone 1 g/day as an IV infusion for 3 days, followed by a rapid dose reduction of 250 mg every 12 hours over 2 days, 125 mg every 12 hours over 2 days, and 60 mg every 12 hours over the next 2 days.
• If persistent ≥ Grade 3 neurotoxicity, consider a repeat neuroimaging (CT or MRI) every 2 to 3 days	• If convulsive status epilepticus as shown by ECG, follow the management approach for status epilepticus in the CAR-T cell therapy.
• If Grade 1 or 2 papilledema and CSF pressure < 20 mmHg, administer acetazolamide 1000 mg as an IV infusion first, followed by an IV infusion of acetazolamide 250 - 1000 mg every 12 hours (the dose may be adjusted base on the patient's renal function and acid-base balance that are examined one to two times daily).<br><br>All the doses are for adult patients<br>* The maximum dose of tocilizumab per injection is 800 mg	• If ≥ Grade 3 papilledema and CSF pressure ≥ 20 mmHg, or brain edema, use the following treatment algorithm:<br>- High doses of corticosteroids; if Grade 4 neurotoxicity, administer IV methylprednisolone 1 g/day.<br>- Raise the patient's bed height at the head to an angle of 30 degrees.<br>- Hyperventilation to cause the patient's partial pressure of carbon dioxide ($PaCO_2$) reach 28 - 30 mmHg, but it should be maintained for no more than 24 hours.<br>- Hypertonic therapy with mannitol 20 g/dL, or 3% or 23.4% hypertonic saline.<br>- If the patient is implanted with ventricular assist device (VAD), draw the cerebrospinal fluids (CSF) to maintain the CSF pressure < 20mmHg.<br>- For patients with EEG burst-suppression patterns, consider a neurosurgical procedure and IV anesthetics.<br>- Perform a metabolic test every 6 hours and head CT daily, and adjust the above drugs used for preventing recurrent brain edema, renal failure, electrolyte disturbance, hypovolemia and hypotension.

Non-convulsive Seizures	Convulsive Seizures
• Maintain airway patency and examine blood glucose concentration.	• Maintain airway patency and examine blood glucose concentration.
• To control status epilepticus detected on the electroencephalogram (EEG), administer lorazepam 0.5 mg as an IV infusion*; if needed, increase by 0.5 mg every 5 minutes, but do not exceed the maximum total dose of 2 mg.	• Transfer to the ICU.
• Levetiracetam 500 mg, IV bolus.	• Lorazepam 2 mg IV infusion* , without the total maximum dose exceeding 4 mg.
• If epilepsy persists, transfer to an ICC and administer IV phenobarbital at a starting dose of 60 mg.	• Levetiracetam 500 mg, IV bolus.
• Maintenance therapy after relief the symptoms of non-convulsive seizures: clorazepam 0.5 mg IV drip Q8h for 3 courses; levetiracetam 1000 mg IV drip Q12h; phenobarbital 30 mg IV drip Q12h.	• If epilepsy persists, administer IV phenobarbita at a starting dose of 15 mg/kg.
	• Maintenance therapy after relief of the

	symptoms of convulsive seizures: lorazepam 0.5 mg IV drip Q8h, for 3 courses; levetiracetam 1000 mg IV drip Q12h; phenobarbital 1-3 mg/kg IV drip Q12h.
	• If status epilepticus is refractory to medical treatment, continue with the EEG monitoring.

Figure 3: Management Principles for Neurotoxicity (Neelapu SS 2017)

7.4 Macrophage Activation Syndrome

Macrophage activation syndrome (MAS) is a serious condition that may be associated with uncontrolled activation and proliferation of CAR+ T cells and activation of macrophages. For subjects treated with JWCAR029, MAS monitoring should be performed and cytokine-directed therapy should be considered as clinically indicated. See IB for details.

7.5 Infusion-related Reactions

Administration of JWCAR029 may be associated with infusion reactions such as fever, rigors, rashes, urticaria, dyspnea, hypotension, and or nausea. To minimize the risk of infusion reactions, prophylactic acetaminophen and diphenhydramine should be administered to all subjects before the investigational treatment. Mild infusion reactions should be treated with antipyretics, antihistamines and antiemetics. Corticosteroids should be avoided not to compromise the efficacy of JWCAR029 cell therapy. Meperidine may be used to treat rigors.

The following guidelines should be followed when managing infusion reactions:

- Grade 1: symptomatic treatment; continue JWCAR029 at the same dose and rate
- Grade 2: symptomatic treatment; slow the rate of JWCAR029 administration
- Grade 3: Withhold JWCAR029, administer symptomatic treatment, and resume at a lower rate after relief of the symptoms. Discontinue JWCAR029 infusion if Grade 3 reaction recurs
- Grade 4: Discontinue JWCAR029, and as necessary, administer symptomatic treatment

7.6 Tumor Lysis Syndrome (TLS)

Both lympho-depleting chemotherapy and JWCAR029 infusion have the potential to cause TLS in subjects with high lymphoma loads. Subjects should be closely monitored for laboratory evidence of TLS (hyperuricemia, hyperkalemia, hyperphosphatemia, and hypocalcemia) and subjects at high risk should be given prophylactic treatment as per standard clinical practice.

7.7 B-cell Dysplasia

B-cell dysplasia is a non-antitumor toxicity caused by targeting non-malignant CD19+ B cells. The main risk of B-cell dysplasia is hypogammaglobulinemia that increases the risk of infection. In subjects with hypogammaglobulinemia (serum IgG < 500 mg/dL), intravenous immunoglobulin should be administered taking into account serum immunoglobulin level.

7.8 Graft-Versus-Host Disease (GVHD)

The likelihood of developing GVHD with CAR+ T cell therapy is low, but theoretically the risk remains. See JWCAR029 IB for details.

7.9 Uncontrolled T-cell Hyperplasia

CAR+ T cells, including JWCAR029, could theoretically proliferate out of control. In the event of uncontrolled JWCAR029 T-cell proliferation, subjects may be treated with high-dose steroids (e.g., methylprednisolone 2 mg/kg/day, followed by tapering over 2-3 weeks until discontinuation) or lympho-depleting doses of cyclophosphamide (1-3 g/m^2 IV). If suspected uncontrolled proliferation of JWCAR029 cells, the investigator should contact the Sponsor immediately.

7.10 Replication-competent Lentivirus, Clonality and Insertional Mutagenesis

Lentiviral vectors used for gene transduction are replication-defective. However, replication-competent lentiviruses (RCL) may still be generated during production. Modern vector generation systems have been improved to reduce the risk of RCL generation. To date, there have been no reports of RCL generated during lentiviral vector manufacturing, which may be, at least in part, due to the use of self-inactivating vectors like those used for generating JWCAR029 (Rothe 2013).

Concern about the potential integration of the vector into the host genome arose from the development of retroviral-mediated malignant transduction in the in vivo preclinical studies in mice (Li 2002, Modlich 2005) and monkeys (Donahue 1992), and from the development of leukemia reported in subjects with X-linked severe combined immunodeficiency (SCID) who received retrovirally modified CD34 + hematopoietic stem cells in a clinical study (Hacein-Bey-Abina 2003), and one of the subjects died (Couzin 2005). Notably, no examples of RCL, clonality or insertional mutagenesis have been reported in animals or subjects to date.

Recently reported data on integration sites of T cells-modifying retroviral and lentiviral vectors emerged from clinical trials (Wang 2009, Scholler 2012, McGarrity 2013), and no clonality of the integration sites was observed. Furthermore, no enrichment of integration sites occurred in the vicinity of genes involved in clonal expansion, indicating that the use of inactivated lentiviral vectors is safe and reliable.

7.11 Risks Associated with Lympho-depleting Chemotherapy

Subjects will receive fludarabine and cyclophosphamide before treatment with JWCAR029 to facilitate lymphodepletion and CAR+ T cell engraftment. For details about the risks associated with fludarabine and cyclophosphamide, please refer to corresponding package inserts.

8 STUDY ASSESSMENTS AND PROCEDURES

Procedures to be performed at all study visits are listed by visit in the schedule of study procedures. See Section 8.5 for details. AEs/SAEs must be reported according to the

procedures and timing of the study and as described in Section 9.3.3. Concomitant medications will be reported according to the timing and contents specified in Section 6.4.6.

Unless otherwise specified, all study procedures and clinical laboratory tests specified on the day of drug administration must be completed prior to drug administration (lympho-depleting chemotherapy or JWCAR029). The investigator must be aware of the subjects' results of clinical laboratory assessments prior to administration of lympho-depleting chemotherapy or JWCAR029. The investigator may perform unscheduled tests for clinical safety variables depending the subject's actual condition, e.g., cytokines, hematology, blood biochemistry, coagulation and inflammatory markers.

8.1 Study Procedures

The entire trial consists of three parts: pre-treatment, during-treatment and post-treatment (see the Schedule of Study Procedures).

Pre-treatment procedures include screening, leukapheresis and pre-treatment assessment, starting from eligibility assessment on the subjects. If a subject is considered eligible, leukapheresis will take place as soon as possible, followed by assessments prior to lympo-depleting chemotherapy and JWCAR029 administration. All subjects will receive lympho-depleting chemotherapy prior to the first dose of JWCAR029. All subjects will be assessed for response approximately 28 days after JWCAR029 administration.

Post-treatment follow-up will include follow-up for safety and disease progression at approximately 2, 3, 6, 9, 12, 18, and 24 months after JWCAR029 administration. The 24-month visit will be the EOS visit.

8.2 Screening

The screening process begins on the date of the subject signing the IRB/EC-approved Informed Consent Form (ICF) and continues until enrollment (or screen failure). If a subject received outpatient assessments similar to the screening procedure within 30 days prior to the informed consent process, the results of such assessments may be used for assessment of eligibility for the study after discussion with Mingju. The following assessments will be performed during the screening period prior to enrollment:

- Informed consent
- Inclusion and exclusion criteria: the subjects eligible for the study must meet all of the inclusion and exclusion criteria
- Medical history, including diagnosis and current disease, history of hematopoietic stem cell transplantation (HSCT), history of chemotherapy, radiotherapy and/or surgery, and history of prior gene therapy. This may also include history of previous treatment toxicity and allergy
- Physical examination

- ECOG performance status (PS)
- Neurological assessment
- Echocardiogram (ECHO) or multiple uptake gated acquisition (MUGA)
- Local laboratory tests:
    - Blood biochemistry, including glucose, blood urea (nitrogen), creatinine, uric acid, sodium, potassium, chloride, calcium, magnesium, phosphorus, total protein, albumin, total and direct bilirubin, alkaline phosphatase, creatine kinase and its isoenzymes, lactate dehydrogenase, ALT, AST, total cholesterol, and triglycerides
    - Hematology, including red blood cell count, white blood cell count, platelet count, neutrophil count, lymphocyte count, monocyte count and hemoglobin content
    - Testing for infectious disease, including HIV, hepatitis B virus (HBsAb, HBsAg and HBcAb), hepatitis C virus (HCVAb), and syphilis
    - Pregnancy test (serum β-HCG) for women of childbearing potential
- If the PET-CT scan after the last anticancer treatment cannot confirm the presence of PET-positive lymphoma, or if the PET-CT scan is beyond 30 days from the informed consent process, the investigator should perform a repeat PET-CT scan to confirm the presence of lymphoma
- Biopsy, if the subject has detectable lesions and is willing to take such as procedure at baseline. For patients who achieved SD after 3 cycles of first-line treatment, if a biopsy is not available at baseline, the investigator should confirm that the subject has archived prior tumor biopsy samples that are available for assessment.
- Record all AEs/SAEs and concomitant medications related to study procedures

8.3 Leukapheresis

Subjects considered eligible for the study should be immediately scheduled for leukapheresis. The following procedures should be completed:

- Complete blood count (CBC) with differential on the day of leukapheresis (or within 24 hours). CBC must include absolute lymphocyte count (ALC)
- Vital signs (before and after leukapheresis)
- Collection of cells by leukapheresis
- Record all AEs/SAEs and concomitant medications related to leukapheresis

8.4 Study Visits

8.4.1 Bridging Anticancer Therapy between Leukapheresis and JWCAR029 Infusion

During the period of JWCAR029 generation (i.e., after leukapheresis but before lympho-

depleting chemotherapy), bridging anticancer therapy, if required, is allowed to control the disease. Low-dose chemotherapy (e.g., ibrutinib, chlorambucil, idelalisib, venetoclax, cyclophosphamide ≤ 300 mg/m^2) is permitted. If there are other non-irradiated lesions, local radiotherapy is allowed for single lesions or some of the lesions. If bridging anticancer therapy is required during this period, pre-treatment PET and contrast-enhanced CT assessments along with diagnostic bone marrow aspiration and biopsy (BMA/BMB) must be performed after completion of anti-tumor therapy. Subjects must continue to meet the eligibility criteria for measurable disease, organ function, active infection, pregnancy, and washout from prior therapy before initiation of lympho-depleting chemotherapy. If a delay in lympo-depleting chemotherapy for more than 14 days from the original estimated start time, discussion with the Sponsor should be held and re-screening may be required.

8.4.2 Pre-treatment Assessment (after Enrollment and before Lympho-depleting Chemotherapy)

Pre-treatment assessments must be done within 7 days prior to lympho-depleting chemotherapy and as well as after completion of any anticancer intervention. Subjects must continue to meet the eligibility criteria for organ function, concomitant diseases, active infection and washout from prior therapy in order to remain in the study. Pre-treatment assessments will be performed according to the Schedule of Activities (SoA).

Pre-treatment assessments include the following tests:

- Fresh tumor biopsy (for subjects with detectable mass and as deemed feasible by the investigator).[1] Immunohistochemistry (including BCL-2, BCL-6, MYC, and CD19 at a minimum) and other tests; for patients who achieved SD after 3 cycles of first-line treatment and a biopsy is not available, the archived tumor samples, if any available, may be used for testing
- Bone marrow aspiration and biopsy (BMA/BMB) (all Subjects) [1] for morphological assessment, as well as immunohistochemistry (including CD19) if presence of any tumor cells
- IPI score (see Appendix E)
- MMSE
- 12-lead electrocardiogram (ECG) [1]
- PET-CT, as well as enhanced CT scans of the neck, chest, abdomen and pelvis (it is recommended that the scan be completed within 7 days before the start of lympho-depleting

1For subjects who received anticancer therapy for the control of the disease during the period of JWCAR029 preparation, the following assessments must be completed after completion of anticancer intervention and as close as possible to the start of lympho-depleting chemotherapy (within 7 days up to 14 days before the start of chemotherapy, as recommended): fresh tumor biopsy (applicable for subjects with palpable masses and as deemed feasible by the investigator), bone marrow aspiration/biopsy (BMA/BMB), and ECG.

chemotherapy, but not more than 6 weeks before lympho-depleting chemotherapy; if the subject received no bridging anticancer therapy and previously underwent PET-CT and enhanced CT scans at the study hospital, these scans are not required; CT scan is not limited to the neck, chest, abdomen and pelvis, and enhanced CT or MRI scan can be used depending on the site of disease)

- Collection of study samples (as baseline control; please refer to the central laboratory manual for details) for:
  - Immunogenicity
  - PK/PD
  - Transgene copy number of JWCAR029 in peripheral blood by qPCR
  - Cytokines

The following assessments will be completed within 7 days prior to lympho-depleting chemotherapy:

- Subject eligibility for enrollment into the study (see Section 5.1)
- Physical examination
- Height/weight
- Vital signs
- ECOG PS score
- Neurological assessment
- Mini-Mental State Examination (MMSE, see Appendix D)
- Hospital laboratory tests:
  - Blood biochemistry, including glucose, blood urea (nitrogen), creatinine, uric acid, sodium, potassium, chloride, calcium, magnesium, phosphorus, total protein, albumin, total and direct bilirubin, alkaline phosphatase, creatine kinase and its isoenzymes, lactate dehydrogenase, ALT, AST, total cholesterol, and triglycerides
  - Hematology, including red blood cell count, white blood cell count, platelet count, neutrophil count, lymphocyte count, monocyte count and hemoglobin content
  - Coagulation function tests (coagulation function, including prothrombin time [PT)], activated partial thromboplastin time [aPTT], international normalized ratio [INR], fibrinogen and D-dimer)
  - Inflammatory markers (including C-reactive protein and ferritin)
  - Immunoglobulins (including IgA, IgG, and IgM)
  - Pregnancy test (serum β-HCG) for women of childbearing potential (within 48 hours

prior to initiation of lympho-depletion chemotherapy)

- Record all AEs/SAEs and concomitant medications related to study procedures

8.4.3 Lympho-depleting Chemotherapy

8.4.3.1 Treatment Criteria

The subject eligibility must be confirmed within 7 days prior to initiation of lympho-depleting chemotherapy. The subjects must have recovered from any adverse events related to chemotherapy and meet the requirement for washout period as shown in Table 5.

Table 5: Washout Period Prior to lympho-depleting Chemotherapy

Drugs	Washout
Ibrutinib	May continue until Day -1
Chlorambucil	7 days
Idelalisib	7 days
Venetoclax	7 days
Cyclophosphamide	7 days
Bendamustine	7 days
Mechlorethamine	7 days
Fludarabine	7 days
Ofatumumab	7 days
Obinutuzumab	7 days
Rituximab	7 days
Lenalidomide	7 days
Methylprednisolone	3 days

Serum creatinine test should be performed within 48 hours prior to lympho-depleting chemotherapy. If serum creatinine is > 1.5 × upper limit of normal (ULN), or creatinine clearance (calculated by Cockcroft-Gault formula; see Appendix C) is ≤ 50 mL/min, the necessity of postponing the lympho-depleting chemotherapy should be determined after communication with the Sponsor. If a delay of more than 14 days in lympho-depleting chemotherapy, it should also be discussed with the medical monitor and re-screening may be required.

8.4.3.2 Lympho-depleting chemotherapy (must be completed within 2 days to 7 days prior to administration of JWCAR029)

Lymphodepleting chemotherapy should be initiated upon notification of JWCAR029 availability from the Sponsor to be completed within 2 to 7 days before JWCAR029 administration. Subjects will receive fludarabine (25 mg/m^2) and cyclophosphamide (250 mg/m^2) for 3 days. The recommended order and timing of drug administration is as follows:

1) 1 L of 0.9% NaCl in water IV at 500 mL/h starting 2 hours prior to cyclophosphamide administration

2) Fludarabine 25 mg/m^2 IV over 30 minutes

3) Cyclophosphamide 250 mg/m^2 IV over 60 minutes

4) Additional 1 L of 0.9% NaCl at 500 mL/h

The volumes and rates of fluids and other treatment measures (including medication) can be determined by the investigator as appropriate depending on the subject's age and condition.

According to the practice of the study site, antiemetics may be given before lympho-depleting chemotherapy and mesna may be administered to subjects with a history of hemorrhagic cystitis.

Following lympho-depleting chemotherapy and before formal infusion of JWCAR029, the following assessments can be performed as appropriate:

- Vital signs
- Laboratory assessments: blood biochemistry and hematology
- ECOG PS score
- Record all AEs and concomitant medications

8.4.4 Criteria for JWCAR029 Treatment

It is advised that the subject be admitted into the hospital to receive JWCAR029 treatment. Observation through at least Day 14 is recommended for the following subjects:

- Subjects who do not have appropriate caregiver support
- Subjects who live in places that require a travel time of more than 60 minutes to the study site during the treatment period.

If a change in the manufacturing process, the Sponsor may require the subject to receive treatment in an inpatient setting until the subject's safety is confirmed.

Subjects should not experience a significant deterioration in clinical status compared to the initial eligibility criteria, because this would result in an increased risk of adverse events related to JWCAR029 infusion. Administration of JWCAR029 should be postponed if the subject meets any one or more of the following criteria on the appointed day of JWCAR029 infusion:

- Suspected or active systemic infection
- Fever ≥ 38°C/100.4°F, not related to underlying disease
- Abnormalities on chest X-ray, or oxygen requirement to maintain oxygen saturation > 91%
- Arrhythmia uncontrolled by medical management
- Hypotension requiring vasopressor maintenance
- New or worsening other non-hematologic organ dysfunction ≥ Grade 3

- Use any of the prohibited medications described in Section 6.5

For subjects with an active infection, JWCAR029 infusion must be postponed until the active infection has resolved (subjects with suspected/active infection must be negative for 24-hour blood culture or rapid multiplex virus testing after appropriate antibiotic treatment). Subjects with organ toxicity may not receive JWCAR029 until the organ toxicity has resolved to ≤ Grade 2. If infusion is delayed, lympo-depleting chemotherapy may need to be repeated after discussion with the Sponsor.

The subjects should be given acetaminophen 450-650 mg orally and diphenhydramine hydrochloride 20-50 mg (PO or IV) 30 to 60 minutes prior to administration of JWCAR029. These drugs may be administered prophylactically or replaced with other drugs of the same class before infusion of the investigational product at the investigator's discretion on a case-by-case basis. Based on the investigator's assessment of the symptoms, these drugs may be administered repeatedly as needed. Pre-intervention with steroids should be avoided.

8.4.5 JWCAR029 Treatment

<u>Day 1</u>

- Physical examination
- Weight measurement
- Vital signs (measured 15 minutes before, during, as well as 15, 30, 45 and 60 minutes after dosing; measurement will be performed hourly from the first hour until 4 hours after dosing, with a window of ± 10%. If the subject is found of unstable vital signs within 4 hours after dosing, vital signs should be monitored according to the clinical routine practice until stabilization)
- ECOG PS score
- Neurological assessment
- MMSE (see Appendix D)
- Local laboratory tests:
    - Blood biochemistry
    - Hematology
    - Coagulation function
    - Inflammatory markers
- Collection of study samples (refer to the central laboratory manual for details) for:
    - PK testing: To detect the number of JWCAR029 (EGFRt+) cells and T cell subsets (CD3, CD4, CD8, CD14, CD16, CD45) in peripheral blood specimens by flow cytometry, and to detect the transgene copy number of JWCAR029 in peripheral

blood by qPCR

- PD testing: To detect the number of CD3, CD19, CD14, CD16 and CD45 lymphocytes in the peripheral blood by flow cytometry.
- Cytokines (IL-6, IL-8, TGF-β 1, TNF-α, IL-2, IFN-γ, IL15 and MCP1 levels in serum by ELISA or MSD)
- qPCR to detect the transgene copy number of JWCAR029 in peripheral blood

- Record all AEs and concomitant medications
- JWCAR029 treatment (started 2-7 days after lympho-depleting chemotherapy)

Days 2, 3 and 4 (+1 day)

- Vital signs
- Neurological assessment
- MMSE (see Appendix D)
- Local laboratory tests:
  - Blood biochemistry
  - Hematology
  - Coagulation function
  - Inflammatory markers
- Collection of study samples (refer to the central laboratory manual for details) for:
  - PK/PD testing
  - qPCR to detect the transgene copy number of JWCAR029 in peripheral blood
  - Cytokine testing
- Record all AEs and concomitant medications

Day 8 (± 1 day)

The procedures are the same as for Days 2, 3 and 4, except for the addition of the following two assessments:

- Physical examination
- ECOG PS score

Day 11 (± 1 day)

The procedures are the same as for Day 8, except for the addition of the following assessment:

- Tumor biopsy (for subjects with detectable mass and as deemed feasible by the investigator): the specimens may be collected between Day 8 and Day 14 for

immunohistochemistry (including, at a minimum, BCL-2, BCL-6, MYC and CD19)

Days 15, 22, and 29 (± 2 days)

- Physical examination
- Vital signs
- ECOG PS score
- Neurological assessment
- MMSE (on Days 15 and 29 only, see Appendix D)
- Local laboratory tests:
  - Blood biochemistry
  - Hematology
  - Coagulation function
  - Inflammatory markers (required on Day 15; but also on Days 22 and 29 if clinically indicated)
  - Immunoglobulins
- Collection of study samples (refer to the central laboratory manual for details) for:
  - Immunogenicity (not on Day 22)
  - PK/PD testing
  - qPCR to detect the transgene copy number of JWCAR029 in peripheral blood
  - Cytokines
- PET-CT, as well as contrast-enhanced CT scans of the chest, neck, abdomen and pelvis to assess response (CT or MRI scan may be used depending on the site of disease) (to be completed during the visit from Day 22 to Day 29)
- Record all AEs and concomitant medications

8.4.6 Post-treatment Follow-up Visits

For all subjects, including those who withdrew from treatment early and those who progressed, a post-treatment follow-up visit should be completed at approximately 60, 90, 180, 270, 365, 545, and 730 days (EOS) after JWCAR029 infusion to assess their disease and survival status. The following assessments will need to be completed for subjects who do not receive subsequent anticancer therapy:

- Physical examination (not required on Day 545)
- Neurological assessment (on Day 90 only)

- MMSE (on Day 90 only, see Appendix D)
- Local laboratory tests (follow the PET-CT/CT scan visit window)
  - Biochemistry
  - Hematology
  - Coagulation function
  - Inflammatory markers (including but not limited to CRP and ferritin)
- PET-CT scan (not required on Day 60; not required if the subject has achieved CR, is assessed as PD, or has started subsequent anticancer therapy; if PD, PET scan is required for confirmation)
- Enhanced CT scan (not required on Day 60; CT scan is not required if the subject is assessed as PD or has started subsequent anti-tumor therapy)
- For patients with a new CR on radiological imaging, diagnostic bone marrow aspiration/biopsy (BMA/BMB) for morphological assessment is required if presence of bone marrow involvement at baseline; immunohistochemistry is required if presence of tumor cells, including CD19
- Immunoglobulins (not required if B-cell recovery and no IVIG treatment recently)
- Collection of study samples (refer to the central laboratory manual for details) for:
  - Immunogenicity
  - PK/PD testing (not required on Days 545 and 730)
  - qPCR to detect the transgene copy number of JWCAR029 in peripheral blood (not required on Days 545 and 730 for subjects without vector sequence identified in previous assay)
  - Cytokines
- Record all AEs/SAEs (as described in Section 9.3) and concomitant medications (as described in Section 6.4.6)

For subjects who have received subsequent anticancer therapy, the following procedures should be completed:

- Collection of information about anticancer therapy, starting immediately after the end of JWCAR029 infusion
- Physical examination on Day 365 and Day 730
- Immunoglobulins (not required if B-cell recovery and no IVIG treatment recently)
- Collection of study samples where possible (please refer to the central laboratory manual for details)

    - qPCR to detect the transgene copy number of JWCAR029 in peripheral blood (not required on Day 545 and Day 730 for subjects without vector sequence identified in previous assay)
- Record all AEs and concomitant medications related to JWCAR029 infusion and/or lympho-depleting chemotherapy

8.5 Unscheduled Visits

Unscheduled assessments may be performed for subjects when assessments are deemed necessary at other visits than those specified in the protocol. The following procedures may be performed as clinically indicated:

- Physical examination
- Vital signs
- ECOG PS score
- Clinical laboratory tests
- PET-CT/enhanced CT scan
- Diagnostic tumor biopsy
- Diagnostic bone marrow aspiration/biopsy (BMA/BMB)
- Spinal puncture for CSF examination
- Collection of study samples (refer to the central laboratory manual for details) for:
    - Immunogenicity
    - PK/PD testing (if the subject has received other lymphotoxic anticancer therapy, radiotherapy, or surgery after disease progression, PK samples should be collected until CAR+ T cells cannot be detected)
    - Transgene copy number of JWCAR029 in peripheral blood by qPCR
    - Cytokines

8.6 Assessments after Disease Progression/Relapse

The following procedures should be performed as soon as possible after disease progression/relapse:

- Diagnostic tumor biopsy as clinically indicated
- Diagnostic BMA/BMB as clinically indicated
- Collection of study samples (refer to the central laboratory manual for details) for:
    - Immunogenicity

- PK/PD testing
- Transgene copy number of JWCAR029 in peripheral blood by qPCR

8.7 Early Withdrawal

Subjects withdrawing from the study early will be scheduled for a visit as soon as to complete all assessments required for the EOS visit. The reason for early withdrawal will be recorded in the CRF.

For subjects who received leukapheresis only, or leukapheresis and lympho-depleting chemotherapy without JWCAR029 treatment, adverse events related to study procedures that occurred within 30 days after leukapheresis or lymphodepleting chemotherapy and before start of new anticancer therapy will be collected; in addition, a telephone follow-up will be conducted every 2 to 3 months to collect information about any new anticancer therapy started and survival status, until the subject has withdrawn from the study, or is lost to follow-up or dead, or the study ends.

8.8 Long-term Follow-up

As this clinical trial involves gene therapy, long-term follow-up will continue through into 24 months after JWCAR029 infusion, regardless of the subject's disease condition.

8.9 Study Assessments

All study assessments should be performed at the timing specified in the Schedule of Activities (SoA) as provided in Appendix A.

8.9.1 Safety Evaluation

Safety evaluation will include physical examination, clinical laboratory tests, neurological examination, as well as AEs/SAEs collected at each visit.

8.9.2 Physical Examination

Physical examination should include assessment of the following body parts/systems: abdomen, extremities, heart, as well as respiratory and nervous systems. In addition, the examination should be carried out depending on the symptoms.

8.9.3 MMSE

The MMSE can be administered by appropriately trained healthcare professionals (i.e., physician, nurse) and does not require a neurologist. Where possible, the MMSE for all subjects should be administered by the same healthcare professional to ensure consistency of assessments.

8.9.4 Vital Signs

Vital signs include body temperature, respiratory rate (RR), heart rate (HR), blood pressure (BP), and oxygen saturation.

8.9.5 Neurological Assessment and Examination

Neurological assessment mainly includes alerted state of consciousness, alerted mental status, speech disorder, ataxia and gait disturbance, motor muscle strength abnormalities, cranial nerve abnormalities, epilepsy, tremors, muscle spasms, etc. If clinically indicated (e.g., new CNS symptoms or presence of suspected CNS lymphoma), CSF examination and CNS imaging (including but not limited to CT, MRI) should be performed both before and after administration of JWCAR029. CSF examination will include cell count with differential. CSF culture (bacterial, fungal, viral) should be performed for subjects with suspected infection.

8.9.6 Response Assessment

Treatment response will be evaluated through enhanced CT scans (chest, neck, abdomen and pelvis) and PET scans of the tumor at specified time points of the study. PET-CT and CT scans and assessments will be performed at each study site. For subjects with known allergy to the contrast medium of enhanced CT scan, non-enhanced CT (or MRI) scan may be used after discussion with the Sponsor. Treatment decisions will be based on the investigator's response assessment.

If a subject has bone marrow involvement confirmed at baseline, or is suspected of bone marrow involvement based on abnormal peripheral blood cell count or blood smears, confirmation of CR during response assessment should be performed in combination with morphology and immunohistochemistry through diagnostic BMA/BMB. PET-CT scan is not required once a subject has achieved CR, unless a possible PD as shown by the follow-up CT scan. Radiological tumor response assessment is no longer required once PD is documented or additional anti-cancer therapy is established.

Response assessment will be performed as per the Lugano criteria (Cheson 2014), and the duration of response will be determined according to the procedures specified in the Schedule of Activities (SoA) for the clinical trial.

8.9.7 Clinical Laboratory Tests

Requirements for screening and clinical laboratory tests are listed in the SoA and Table 6.

For each subject, the investigator will assess whether the results of various tests at each visit are out of the normal reference ranges and make a judgment as to whether they are clinically significant. This judgment should be based on the characteristics of the laboratory test and the degree of abnormality. Due attention should be given to abnormalities that are considered clinically significant and/or related to JWCAR029 treatment. The investigator may choose to repeat the test on any abnormal results to rule out laboratory or sample collection errors.

Table 6: Analytes for Clinical Laboratory Assessments

Laboratory Tests	Analyte
**Blood biochemistry**	Including glucose, blood urea (nitrogen), creatinine, uric acid, sodium, potassium, chloride, calcium, magnesium, phosphorus, total protein, albumin, total and direct bilirubin, alkaline phosphatase, creatine kinase and its isoenzymes, lactate dehydrogenase, ALT, AST, total cholesterol, and triglycerides
**Hematology**	CBC with differential
**Coagulation function**	PT/aPTT, INR, fibrinogen, and D-dimer
**Testing for infectious disease**	HIV
	Hepatitis B Virus (HBsAb, HBsAg and HBcAb)<br>Hepatitis C virus (HCVAb)<br>Syphilis
**Inflammatory markers**	CRP, ferritin
**Immunoglobulins**	IgG、IgM、IgA
**CSF**	Protein, cell count

8.9.8 ECOG Performance Status

ECOG performance status (see Table 7: ECOG Performance Status) is used for assessing the subject eligibility at screening, and will be assessed at the time points specified in the SoA during the study.

Table 7: ECOG Performance Status

Grade	Description
0	Fully active and able to carry out all pre-disease performance without restriction
1	Restricted in strenuous activity but ambulatory and able to carry out work of a light or sedentary nature, e.g., light house work, office work
2	Ambulatory and capable of all self-care but unable to carry out any work activities. Up and about more than 50% of waking hours
3	Capable of only limited self-care; confined to bed or chair > 50% of waking hours.
4	Completely disabled; cannot carry on any self-care; totally confined to bed or chair

Reference: Oken 1982

8.9.9 MUGA/Echocardiogram

Echocardiogram or MUGA is used to measure left ventricular ejection fraction (LVEF) to determine the subject's cardiac function and possibility to participate in the study.

8.9.10 ECG

Standard 12-lead ECG will be performed. Each ECG tracing image should be signed and dated by the investigator and retained in the source documents at the study site.

8.9.11 Study Samples

In principle, the testing and analysis of samples will be conducted in accordance with the study procedures and schedule of the trial. If a limited number of samples collected, the sample

allocation may also be modified. However, the total number and type of samples collected will not exceed the range specified in the study protocol.

PK/PD Parameters

The PK parameters of JWCAR029 will be assessed by means of qPCR for the transgene copy number of JWCAR029 in peripheral blood, and flow cytometry for the number of JWCAR029 cells (EGFRt+) and T-cell subsets (CD3, CD4, CD8) in subject's whole blood. The PD parameters will be assessed through flow cytometry for B-cell subsets (CD19) in the subject's whole blood. Peripheral blood samples will be collected at the time points specified in the study protocol.

Immunogenicity Testing

The humoral immune response to JWCAR029 will be tested in this study. The humoral immune response measures the binding of anti-drug antibodies (ADA) to the extracellular domain of JWCAR029 in plasma. Peripheral blood samples will be collected at the time points specified in the study protocol.

Details regarding the collection, processing and shipping of samples for immunogenicity assessment are provided in the central laboratory manual.

Cytokines

Serum cytokines will be used as the markers of immune activation by JWCAR029 to analyze the potential correlations between cytokine production and response as well as CRS severity. Serum levels of IL-2, IL-6, IL-8, TGF-β 1, IL-15, TNF-α, IFN-γ and MCP1 can be measured by ELISA.

Serum samples will be collected at the time points specified in the study protocol.

9 SAFETY OVERSIGHT AND REPORTING

9.1 Definitions

9.1.1 Adverse Event

An adverse event (AE) is any untoward medical occurrence in a clinical investigation subject administered a pharmaceutical product and which does not necessarily have a causal relationship with this treatment (i.e., JWCAR029).

9.1.2 Serious Adverse Event

A serious adverse event (SAE): An adverse event is considered serious when it meets any one or more of the following criteria:

- Results in death:

  Based on the study protocol, death is an efficacy endpoint. Deaths occurring during the protocol-specified AE reporting period that are judged by the investigator to be solely due to B-NHL progression should only be recorded on the EOS/Early Termination eCRF. All

deaths occurring during other study procedures, regardless of relationship to the investigational product, must be recorded on the Adverse Event eCRF and reported immediately to the sponsor

- Life-threatening:

  An AE or adverse reaction is considered "life-threatening" if the subject is considered at risk of death at the time of the event by the investigator or the Sponsor. It does not refer to an event which hypothetically might have caused death if it were more severe.

- Requires inpatient hospitalization or prolongation of existing hospitalization (see "Notes" below)
- Results in disability/incapacity; or significantly interferes with the subject's ability to carry out activities of daily living
- Is a congenital anomaly/birth defect of the newborn resulting from fetal exposure to the investigational product before or during pregnancy;
- Is considered an important medical event by the investigator:

  That may jeopardize the subject or require medical or surgical intervention to prevent one of the other outcomes listed in the definition above. Examples of such events are intensive treatment in an emergency roomor at home for allergic bronchospasm; blood dyscrasias or convulsionsthat do not result in hospitalization.

In addition, based on the guidance for industry "Gene Therapy Clinical Trials -Observing Subjects for Delayed AEs", any of the following clinical symptoms occurring through lympho-depleting chemotherapy to the end of study should be reported as an SAE unless the event can be clearly attributed to another cause.

- New/secondary malignancy
- New incidence or exacerbation of a pre-existing neurologic disorder
- New incidence or exacerbation of a prior rheumatologic or other autoimmune disorder
- New incidence of a hematologic disorder
- Rare and unforeseen disorders of unknown etiology (e.g., Guillian-Barre syndrome, Stevens-Johnson syndrome)

Notes:

The following types of hospitalization will not be considered an SAE in this study:

- Admission to the hospital based on the requirements of the protocol and signed informed consent
- Admission for social or situational reasons (e.g., no accommodation place, living far away from the hospital)

- Hospitalization at the discretion of the investigator for procedures required by the protocol (e.g., mononuclear cell collection, lympho-depleting chemotherapy, JWCAR029 administration) or to facilitate clinical monitoring
- If a subject was discharged early after an observation period of less than 14 days following infusion of the investigational product upon assessment by the investigator due to shortness of beds in the department and then hospitalized for inpatient treatment and clinical monitoring due to fever or other CRS or neurological symptoms but did not experience ≥ Grade 3 CRS or neurotoxicity during the period of inpatient treatment and clinical monitoring, such an AE will be documented in the summary report of AESI and will not be reported as an SAE.
- Elective or pre-planned hospitalization for a pre-existing condition that is unrelated to the disease studied and has not worsened since the signing of the ICF
- Admission to the hospital for routine tests or treatments related to the disease studied (e.g., bone marrow aspiration, platelet transfusion, bridging chemotherapy. However, complications and/or pronged hospitalization due to routine treatment should be reported as an SAE)

9.2 Clinical Laboratory Test Abnormalities and Other Assessment Abnormalities

Clinical laboratory test abnormalities (e.g., clinical biochemistry or hematology) and other examination abnormalities (e.g., ECG or vital signs) meeting the following criteria should be recorded as AE or SAE:

- Clinically significant as confirmed by the investigator
- Requiring medical or surgical intervention
- Resulting in discontinuation, delay or interruption of product administration
- Related to clinical signs and/or symptoms

Laboratory abnormalities that are not clinically significant should not be recorded as AE or SAE.

Whenever possible, the investigator should report a clinical diagnosis rather than laboratory results (e.g., anemia vs. decreased hematocrit) at all times.

Clinically significant abnormal laboratory findings occurring during the study will be followed up until the results of repeat test return to normal, become stable, or are no longer clinically significant.

9.3 Evaluation of AEs and SAEs

Each AE and SAE will be evaluated for duration (onset date and end date), severity (including change in the grade per the CRF completion guidelines), outcome, seriousness and causal relationship with JWCAR029 infusion, lympho-depleting chemotherapy, and/or study-

specified procedures.

9.3.1 Grading and Severity of AEs

The severity of AEs and SAEs (except for CRS) will be graded according to the NCI CTCAE version 5.0. The severity of CRS will be graded according to Lee criteria 2014 (see Section 7.1).

9.3.2 Relationship to Investigational Product

Evaluation of relationship (related or not related) between an AE and JWCAR029, lympho-depleting chemotherapy or study-specified treatments is a clinical judgment based on all available information and the following considerations:

- Certain: There is a temporal plausibility between JWCAR029 infusion, fludarabine or cyclophosphamide administration, and/or study-specified procedures and the AE; the response follows the patterns of known adverse drug reactions (ADRs) (reported in similar literature); disappearance, rapid mitigation or improvement of AE after treatment discontinuation; AE appears again after re-challenge and may worse significantly (positive for re-challenge); the underlying disease and other confounding factors can be ruled out.
- Probable: there is not a history of repeated medication, and the remaining criteria are the same as "Certain"; or despite of concomitant medications, the possibility of AE caused by concomitant medications could be basically ruled out.
- Possible: there is a close temporal relationship between the occurrence of an AE and JWCAR029 infusion, administration of fludarabine or cyclophosphamide, and/or a study-specified procedure; the AE follows the patterns of know ADRs, but could be caused by more than one drugs; the underlying disease or other treatment factors could not be ruled out.
- Unlikely: There is not a close temporal relationship between the AE and the investigational product and/or study-specified procedure; the AE follows the patterns of known ADRs; the AE can be more attributed to the underlying disease or other treatment factors.
- Unrelated: There is no temporal relationship between the AE and the investigational product and/or study-specified procedure; The AE may be caused by other factors.

9.4 Collection and Reporting of AEs

AEs/SAEs will be recorded on the CRF according to reporting criteria defined for different time periods in Section 9.5. Each AE should be assessed for:

- Duration (onset date and resolution date)
- Severity (including a change in the grade as per CRF completion guidelines: see Section 9.3.1)
- Outcome

- Seriousness (see Section 9.1.2)
- Causal relationship with lympho-depleting chemotherapy or JWCAR029 infusion

Reporting of AEs and SAEs:

AEs/SAEs will be recorded according to the periods of time described in Table 8.

Table 8: Timeline for Reporting of AEs

Time Periods	Events to Be Recorded
From the signing of the ICD to 90 days after the last JWCAR029 IV infusion or to the EOS visit, whichever occurs earlier	All AEs/SAEs (including complications due to any procedure, whether or not the procedure is considered protocol-mandated) will be collected
For subjects who start subsequent non-chemotherapeutic anti-cancer therapies (e.g., checkpoint inhibitors, immunomodulators) before 90 days following JWCAR029 administration	All AEs/SAEs will be collected after start of treatment, 90 days after JWCAR029 infusion or 30 days after subsequent treatment, whichever occurs later
For subjects who start subsequent chemotherapy-containing anti-cancer therapy before 90 days following JWCAR029 infusion	Only JWCAR029-related AEs/SAEs will be collected after start of treatment
From 91 days after JWCAR029 infusion until the end of study	Only AEs/SAEs related to JWCAR029 will be collected

Any of the following clinical manifestations occurring from lympho-depleting chemotherapy must be reported as an SAE, unless there is an alternative identifiable cause:

- New/secondary malignancy
- New incidence or exacerbation of a pre-existing neurologic disorder
- New incidence or exacerbation of a prior rheumatologic or other autoimmune disorder
- New incidence of a hematologic disorder
- Rare and unforeseen disorders of unknown etiology (e.g., Guillian-Barre syndrome, Stevens-Johnson syndrome)

When reporting an SAE, the investigator should complete and send an SAE Report Form (paper or electronic) to the Sponsor in no later than 24 hours after awareness of the event. The site staff should also document the SAE information on the AE page in the electronic data capture (EDC) system. Detailed instructions can be found in the Case Report Form (CRF) Completion Guidelines. If a paper SAE Report Form is used, the site staff should, in no later than 24 hours after awareness of the event, send the SAE Report Form and/or supporting documentation to the Sponsor's Drug Safety Department or its agent via e-mail according to the instructions in the SAE Report Form. If the EDC system is used but cannot be accessed temporarily, the SAE information must be entered into the EDC system within 24 hours after access to the EDC system becomes available.

The subjects are allowed to receive emergency treatment at any medical institution. The investigator should collect and submit details and reproduced copies of emergency room/hospital records about such emergency treatment events.

In this case, the investigator must continue to conduct follow-up of the subject until the SAE is resolved, or until the condition becomes chronic, stable (in the case of persistent impairment), or the subject is dead.

The investigator must submit the updated SAE report and/or supporting documentation within 24 hours upon receipt of follow-up information according to the above procedures. In addition, the SAE report must be retained at the study site and archived in the Study File as per the requirements of the facility.

Pursuant to applicable laws and regulations, Shanghai Mingju Biotechnology Co., Ltd should report SAEs and suspected unexpected serious adverse reactions (SUSARs) to relevant authorities within the specified time limit.

9.4.1 Documentation of Diagnosis and Signs & Symptoms

Whenever possible, the investigator should report a unifying diagnosis rather than list individual symptoms. However, the symptoms can be included in the diagnosis only when each sign or symptom is a medical component of the diagnosis (based on the current standard medical textbooks). If the signs or symptoms do not fit a classic diagnosis in any aspect, the individual symptom should be reported as a separate AE.

An exception is CRS, for which the diagnosis should be reported rather than symptoms. If a subject experiences an event of CRS, the individual signs and symptoms of CRS with any change in the grade should be reported as AE separately. The diagnosis of CRS should be entered on a separate CRF page as a CRS event.

When neurotoxicity occurs concurrently with CRS or alone, these manifestations of the neurotoxicity should be reported as separate AEs (e.g., encephalopathy, aphasia, or epileptic seizure). Once neurotoxicity is confirmed, the diagnosis of neurotoxicity should be entered on a separate CRF page as a neurotoxicity event.

9.4.2 Clinical Laboratory Abnormalities and Other Abnormal Assessment Findings

Any laboratory abnormalities (e.g., clinical biochemistry or hematology) or other abnormal assessment findings (e.g., ECG or vital signs) that meets any of the following criteria should be recorded as an AE or SAE:

- Requiring medical or surgical intervention
- Resulting in discontinuation, delay or interruption of the product administration
- Related to clinical signs and/or symptoms
- Clinically significant as confirmed by the investigator

Whenever possible, the investigator should report a clinical diagnosis rather than laboratory results (e.g., anemia vs. decreased hematocrit) at all times.

Clinically significant abnormal laboratory findings occurring during the study will be followed

up until the results of repeat test return to normal, become stable or are no longer clinically significant.

9.4.3 Documentation of Serious Adverse Events

Documentation of SAE should consider the following:

- Death is the outcome of an event. An event that resulted in death should be documented on the CRF and reported in the SAE Report Form (see Section 9.4.4).
- For hospitalization, or any surgical or diagnostic procedure, the condition that led to such surgical or diagnostic procedure should be recorded as an SAE rather than the procedure itself. The procedure should be documented in the "Narrative Comments" as part of the action taken for the condition.
- When progressive disease (PD) meets the criteria for "serious", the specific manifestations of the PD (e.g., "malignant pleural effusion", "lymphadenopathy due to underlying non-Hodgkin lymphoma") should be reported as individual SAEs rather than the general term "Progressive disease". Unless it resulted in death (see Section 9.4.4).

9.4.4 Reporting of Deaths

Given the nature of the disease treated, death is an expected outcome during the study. All deaths must be reported on the Death CRF. Deaths due to disease progression will not be reported as SAE unless they are considered related to the investigational product. Any AE leading to death that occurred during the period from provision of the informed consent by the subject to 90 days after infusion of the investigational product should be reported as SAE.

Deaths occurring > 90 days after JWCAR029 infusion will be recorded on the Death CRF, and will be reported as SAE only when considered related to the study procedures or JWCAR029.

9.4.5 Pregnancy

During the period from the signing of the informed consent document to the end of study, the investigator must report to the Sponsor each pregnancy occurring in a female subject or the female partner of a male subject within 24 hours after awareness. Pregnancy, if detected after start of lympho-depleting chemotherapy, should be followed up until the outcome.

Pregnancy should be recorded on the Pregnancy Report Form and reported to the Sponsor by the Investigator. Pregnancy follow-up information should be recorded on the same form and submitted to the Sponsor within 24 hours after awareness. Any SAE occurring during pregnancy must be reported on the SAE Report Form. Abortions (whether accidental, therapeutic, or spontaneous) should be reported as SAE (except for non-therapeutic induced abortions). A congenital anomaly or congenital birth defect in a newborn, as defined in Section 9.1.2 "Criteria for Serious Adverse Event", should be reported as an SAE.

All pregnancies occurring throughout the study should be monitored, and all perinatal and neonatal outcomes should be reported.

9.5 Reporting and Follow-up of AEs and SAEs

All SAEs (initial or follow-up information) must be reported within 24 hours of awareness. The SAE information should be documented on the SAE Report Form and submitted to the Sponsor's Drug Safety Department.

For initial reporting of SAE, the following minimum information must be reported on the SAE Form:

- Subject number
- Onset date of event
- Event description
- Study treatment
- Judgment on relatedness to study treatment

The completed SAE Report Form and supporting documentation should be sent as instructed in the SAE Report Form.

Each SAE will be followed up until the event has resolved or recovered to baseline, or is considered stable or not clinically significant any more by the investigator, or the subject is dead or withdraws the informed consent, or the study ends. Certain non-serious AEs, as required by the Sponsor, may be followed up until resolution, recovery to baseline or the end of study (EOS).

Relevant follow-up information, once available, should be submitted to the Sponsor immediately.

10 STATISTICAL METHODS

10.1 Comprehensive Consideration

As this study is a single-arm open-label clinical trial, rigorous hypothesis testing will not be performed.

The safety, efficacy and PK/PD data of the study will be analyzed descriptively. Details regarding statistical analysis are provided in the Statistical Analysis Plan (SAP), which will be finalized submitted for review before database lock. Any changes to the pre-defined methods of statistical analysis will be described in the final Clinical Study Report (CSR).

10.2 Data Analysis Sets

10.2.1 Safety Set

The Safety Set (SS) includes all subjects who received any dose of JWCAR029 treatment. This analysis set will be used for analyses of safety endpoints.

10.2.2 Modified Intent-to-Treat (mITT) Set

The mITT set includes all subjects who received any dose of JWCAR029 treatment, but

excludes those subjects who received non-standard investigational product. If bridging therapies received by the subjects prior to JWCAR029 infusion have an impact on the baseline for efficacy evaluation, exclusion of this analysis set will be considered on the basis of medical opinions. This analysis set is intended for efficacy analysis.

10.2.3 Other Analysis Sets

Other analysis sets, such as the PK/PD analysis set, will be defined in the Statistical Analysis Plan (SAP).

10.3 Data Handling Conventions

Unless otherwise specified, all analyses will be performed by "Dose group" and "Combined".

Subject listings will be provided. Summary tables for continuous variables will include the following statistics: N (number of subjects in the analysis set), n (number of non-missing observations), mean, standard deviation (SD), median, minimum and maximum. Summary tables for categorical variables will include: N, n, and percentages. Unless otherwise specified, the 95% confidence intervals for percentages will be calculated using the binomial distribution (exact method) and will be two-sided.

The baseline of primary safety analysis in the statistical analysis is defined as the last non-missing value before JWCAR029 treatment. The last non-missing value prior to lympho-depleting chemotherapy may be used as the baseline for secondary safety analyses. Data from all study sites will be pooled for all analyses. Specific analysis methods and rules for the handling of missing data and data reporting are detailed in the SAP.

10.4 Planned Analyses

10.4.1 Subject Disposition and Baseline Characteristics

For all enrolled subjects, the number and percentage of subjects who entered and completed each period of the study, as well as the number and percentage of subjects who withdrew early in each period, will be statistically described. In addition, the number of subjects included in each analysis set will be summarized.

Demographics and baseline characteristics will be analyzed using descriptive statistics.

10.4.2 Safety Analysis

Safety analysis will be based on the safety set (SS).

10.4.2.1 Adverse events

All AEs will be presented in tabular form. Summary of AEs will focus on treatment-emergent adverse events (TEAEs) and adverse events of special interest (AESI). A TEAE is defined as any AE occurring or worsening within 90 days after JWCAR029 infusion. Any AE occurring after initiation of another anticancer therapy will not be considered a TEAE. Adverse events of special interest (AESI) will also be summarized.

AEs will be reported based on the Medical Dictionary for Regulatory Activities (MedDRA) and graded as per NCI CTCAE version 5.0 (see Section 9.2). TEAEs will be summarized by system organ class (SOC), preferred term (PT), and seriousness. Subjects reported with multiple TEAEs within the same SOC and PT will be counted only once according to the maximum severity grade. Listings and summaries of TEAEs will be prepared according to the following types:

- SAEs
- Grade 3 or above AEs
- Treatment-related AE (lympho-depleting chemotherapy, protocol-mandated procedures, JWCAR029)
- AEs leading to death

AESI, including ≥ Grade 2 CRS and neurotoxicity among other TEAEs. Details regarding analyses will be provided in the SAP.

10.4.2.2 Laboratory data

All laboratory data will be tabulated. Summaries of laboratory data (including hematology, blood biochemistry, coagulation function, inflammatory markers, immunoglobulins, etc.) will focus on treatment-emergent laboratory abnormalities and changes from baseline.

A treatment-emergent laboratory abnormality is defined as a clinical laboratory value that increases at least one toxicity grade from baseline at any time point up to 90 days after administration of the investigational product. However, if a patient received other anticancer therapy within 90 days after administration of the investigational product and the laboratory test results were thus affected, the patient will be included in analysis of laboratory abnormalities detected before initiation of other anti-tumor therapy.

If baseline data are missing, abnormalities of any grade (i.e., abnormalities ≥ Grade 1 in severity) will be considered treatment-emergent. If applicable, laboratory test results will be graded according to CTCAE v5.0; Grade 0 includes all non-missing values as long as these outliers are less than Grade 1; Grade 5 will not be used. In the CTCAE, some laboratory parameters have corresponding grading criteria in two directions (i.e., high and low), in which case the analysis results in each direction will be provided.

Selected analyses will be summarized as follows:

- Raw values and changes from baseline will be summarized by visit and laboratory test, and will be expressed in universal units of measurement
- Number of subjects graded as per CTCAE criteria and corresponding percentage at each visit
- Cross-tabulation will be used to summarize changes in CTCAE severity grade from baseline to post-baseline maximum severity grade: for laboratory tests without CTACAE

grades, the cross-tabulation will present low/normal/high changes from baseline and judgment on clinical significance, if appropriate.

10.4.2.3 Immunogenicity data

Humoral immunogenicity assessments will include morbidity of immunogenicity (subjects with pre-existing antibodies that bind to JWCAR029), incidence of immunogenicity (subjects with treatment-induced or treatment-boosted antibodies that bind to JWCAR029), and antibody titers. Descriptive statistics will be used to determine the proportion of subjects with shifts versus sustained antibody response.

10.4.2.4 Other safety data

Vital signs will be summarized using descriptive statistics and tabulation. Changes from baseline in total MMSE scores at each time point will be summarized. All other safety data will be tabulated.

10.4.2.5 Concomitant medications

Prior and concomitant medications will be coded and tabulated using the World Health Organization Drug Dictionary (WHO-DD). All concomitant medications and blood product transfusions following JWCAR029 infusion will be summarized.

Specific treatments for CRS (i.e., corticosteroids, tocilizumab, etc.) and the mitigation and duration of CRS will be summarized.

10.4.3 Efficacy Analysis of Study Treatment

10.4.3.1 Efficacy analysis

The Clopper-Pearson method will be used to estimate the confidence intervals for investigator-assessed one-month complete response rate (CRR) and overall response rate (ORR). The percentage of subjects with CRR/ORR at the assessment time points of 3 months, 6 months and 12 months (achieving CR or PR at these time points) will be summarized together with the 95% confidence intervals.

Meanwhile, best overall response (BOR) during each period will also be summarized. BOR is defined as the most favorable response recorded from start of JWCAR029 treatment to the time point required for observation, or to the last PD/relapse, or to the start of another anti-cancer therapy. BOR will be assigned in the following order: CR, PR, SD, PD and unknown. Subjects with unknown or unmeasured response will be included in the denominator for the calculation of response rate.

Kaplan-Meier method will be used to describe time-to-event endpoints (including DoR, PFS and OS) in efficacy analysis, presenting the median survival and corresponding confidence intervals. If no event was observed for a subject, it will be treated as censored data, as detailed in the SAP.

Time to first response will be summarized using descriptive statistics, providing the mean

(standard deviation), median, inter-quartile range and range of the time to first response for subjects achieving response.

10.4.4 Pharmacokinetic Analysis

Assays for analysis of various cellular PK parameters include qPCR assay for to detect JWCAR029 cells (transgene copies/μg DNA) in peripheral blood and flow cytometry to detect JWCAR029 cells in peripheral blood. Details regarding the procedures of sample collection for the above assays are provided in the central laboratory manual.

The PK values of JWCAR029 cells will be assessed by flow cytometry by measuring the absolute number of JWCAR029 cells in per μL of whole blood, as well as the absolute number and relative percentage of T-cell subsets (CD4 and CD8) expressing JWCAR029 transgene protein.

For all subjects in the PK analysis set, the PK profile of JWCAR029 cells in peripheral blood will be described using C_{max}, T_{max}, AUC and other relevant PK parameters. The maximum expansion ($C_{max}/C_{Day\ 2\ post\text{-}dose}$) of JWCAR029 in blood and the persistence of JWCAR029 in blood will be measured by means of qPCR (above the lower limit of quantitation, LLOQ) and flow cytometry (exceeding the threshold level of JWCAR029).

Where possible, the following PK parameters will be displayed graphically: concentration of JWCAR029 in peripheral blood versus time by qPCR: concentration of JWCAR029 in peripheral blood versus time by flow cytometry.

The following PK parameters and other relevant PK parameters will be estimated from the individual concentration-time profiles using non-compartmental analysis: AUC, C_{max}, T_{max} and time above the LLOQ. All concentrations below the limit of detection or quantitation (LOD or LOQ), or missing data, will be labeled as such in the concentration data listings. Concentrations below the LOD will be considered as zero in the summary statistics.

Descriptive statistics for PK parameters will be categorized by efficacy and safety outcome measures, and will include mean, SD, coefficient of variation (CV), minimum and maximum. The ranges of values may be presented for the selected variables. The median value and range will be used for T_{max}, which is usually assessed by a non-parametric method.

Finally, the relationship between use of steroids and other relevant co-variables and cellular PK will be explored.

10.4.5 Quality Attributes and Process Performance Attributes of JWCAR029 Product

The product attributes of JWCAR029 (e.g., T-cell subsets, transduction efficiency, in vivo expansion potential and transgene copy number) will be summarized descriptively by selected clinical and safety endpoints.

10.5 Sample Size Considerations

The sample size is primarily determined based on the need of safety assessment rather than estimated based on statistical inference. This study is planned to enroll approximately 12 subjects. Considering the possibility that the subjects may receive non-standard therapies and be replaced during the course of the study, the number of subjects actually enrolled may exceed 12.

10.6 Timing of Analysis

The primary analysis is planned to take place after all subjects have been followed up for at least 1 month after JWCAR029 infusion, or until death, disease progression or withdrawal from study. The final analysis will be performed after all subjects have completed the study or discontinued the study for any reason.

11 DATA MANAGEMENT

11.1 Data Collection System

Shanghai Mingju Biotechnology Co., Ltd will provide the EDC system for data collection. Access to the EDC system is based on role privileges and will be granted to corresponding roles only after completion of specified training.

11.2 Data Quality

The staff of the study site will enter the data into the CRF in the EDC system. The clinical research associate (CRA) or designee of Shanghai Mingju Biotechnology Co., Ltd will verify the data recorded in the CRF against the source documents.

In order to ensure the completeness and accuracy of the data, the automated data verification checks programmed in the EDC system will flag missing or inconsistent data during the data entry process. The entries flagged by automated data verification checks and the project team will appear as electronic queries in corresponding CRF in the EDC system so that they will be resolved by corresponding function-specific users. All data entered and subsequent data changes will be recorded in the audit trail in the EDC system.

The Principal Investigator will be responsible for ensuring that data are entered accurately and completely into the CRFs, and will electronically sign the CRF of each subject before database lock.

After database lock, reproduced copies of the final subject casebooks will be archived at the study site.

12 STUDY ADMINISTRATION

12.1 Regulatory and Ethical Considerations

12.1.1 Regulatory Audit

This study will be conducted in compliance with Good Clinical Practice (GCP) and any other

applicable regulatory requirements.

12.1.2 Approval of Institutional Review Board (IRB)/ Ethics Committee (EC)

The investigator is responsible for ensuring that the protocol is reviewed and approved by the appropriate IRB or EC prior to initiation of the study. The Informed Consent Form (ICF) and other written information provided to the subjects, as well as other materials to be possibly received by the subjects must be reviewed and approved by the IRB/EC.

If the protocol, the Investigator's Brochure (IB), or the ICF is revised during the course of the study, as per regulatory requirements, it is the responsibility of the investigator to ensure that such revised documents are promptly reviewed and approved by the IRB/EC. In other words, all revised documents, including ICF revisions, must be approved by the IRB/EC before they are put into use.

12.1.3 Subject Informed Consent

The investigator or his/her designated qualified person will be responsible for explaining the nature, purpose, benefits, and risks of the study to each subject, the subject's legally acceptable representative, or an impartial witness prior to the start of enrollment in the study. Written informed consent must be obtained from each subject before entry into the study (prior to the start of any study-related procedures). The subjects should be given sufficient time to ask questions and discuss any questions they have. The investigator or the designee should document this process in the study records. The Investigator must use the current IRB/EC-approved Informed Consent Form for documenting the written informed consent. Each ICF should be signed and dated by the subject, the subject's legally acceptable representative, and the person implementing the informed consent process, and (if required by the IRB or EC, or local regulations) an impartial witness. The entire process of informed consent should be implemented in compliance with the requirements of the International Conference on Harmonization (ICH) (ICH E6 Article 4.8), as well as with the requirements of China's applicable laws and regulations, including GCP issued by NMPA.

If the ICF is revised during the course of the study, the study site must provide the newly enrolled subjects with the IRB/EC-approved revised ICF for signing, and repeat the informed consent process for all subjects under study with respect to the revised ICF.

12.2 Obligations of the Investigator

12.2.1 Responsibilities of the Investigator

It is the responsibility of the investigator to ensure that all staff of the study site, including sub-investigator and other study staff, will conduct the study in compliance with Declaration of Helsinki and ICH E6 GCP Guidelines, including the archiving of core documents.

If revision to the study protocol or ICF is necessarily required, the investigator will be responsible for ensuring that the revised document is reviewed and approved by the IRB/EC and that the revised or updated documents are provided to the subjects promptly.

Before initiation of the study, the investigator should provide Mingju with valid business licenses, recent GCP training certificates and curriculum vitaes, and sign the signature pages of the final study protocol and any revised version. All of the above forms must be updated as necessary at any time throughout the study.

12.2.2 Reporting Responsibilities of the Investigator

Pursuant to laws and regulations, the investigator is obliged to independently report the progress of the study to the IRB/EC and notify the IRB/EC of study termination. The investigator must provide Shanghai Mingju Biotechnology Co., Ltd with copies of all IRB/EC letters approving the conduct, update or change of the study. The investigator must also forward the updated documents of the IRB/EC to Shanghai Mingju Biotechnology Co., Ltd.

12.3 Access to Information Monitoring

Pursuant to applicable laws, regulations and guidelines, the designated CRA of Shanghai Mingju Biotechnology Co., Ltd must have the right to access the original medical records of the subjects directly to verify the accuracy of the data recorded in the CRF.

The CRA is responsible for routine review of the CRFs at regular intervals during the course of the study to verify compliance with the study protocol and the completeness, consistency and accuracy of the entered data. The CRA should have access to any required subject records to verify the data entered in the CRF. The investigator should agree to cooperate with the CRA to ensure that all issues identified in any way (remote or site visit) are resolved.

12.4 On-site Audits and Regulatory Inspections of Study Site

The clinical trial may be subjected to on-site audits or inspections by regulatory authorities and the representatives of Shanghai Mingju Biotechnology Co., Ltd or the IRB/EC. The investigator agrees that he or she will notify Shanghai Mingju Biotechnology Co., Ltd immediately upon the receipt of a notification of regulatory inspection. The investigator agrees to provide the representatives of the regulatory authorities and Mingju with access to the trial records, equipment and personnel to facilitate effective audits or inspections.

12.5 Protocol Deviations

Protocol deviations must be reported to the institution's IRB in accordance with its policy. The investigator should understand and observe the requirements of the institution's IRB.

12.6 Quality Assurance and Quality Control

Shanghai Mingju Biotechnology Co., Ltd or its designee will conduct quality control (QC) and quality assurance (QA) inspections on all its sponsored clinical studies. Before enrollment of any subjects into the study, the staff of Shanghai Mingju Biotechnology Co., Ltd will provide the investigator, sub-investigator and the site staff with training on the following: protocol, IB, CRF and study procedures, the informed consent process, and the procedures for SAE reporting. The CRA or designee of Shanghai Mingju Biotechnology Co., Ltd will conduct regular sites

visits throughout the study. During these visits, the information recorded in the RCFs will be verified against source documents and if appropriate, clarification or correction may be required. The CRA will review the CRFs for safety information and the completeness, accuracy and logical consistency of data. Computer programs checking inconsistent data will also help in the clinical study. All required clarifications or corrections will be sent to the investigator in the form of data queries.

12.7 Publication on Study Progress

According to the recommendations of the International Committee of Medical Journal Editors (ICMJE) on the publication of study results, Shanghai Mingju Biotechnology Co. Ltd will be responsible for ensuring that the study will be registered on ClinicalTrials.gov website and information regarding the study design and progress released on the website will be updated appropriately during the course of the study in accordance with the requirements of U.S FDA and NMPA regulations.

12.8 Termination of Study

Once the study has reached the endpoints or is terminated early, the CRA or the investigator should complete the following activities where possible:

- Return all electronic and non-electronic data (such as copies of ECG tracings) to Shanghai Mingju Biotechnology Co., Ltd as required;
- Clarify the CRF data and/or answer queries;
- Check the quantity and reconciliation list of the investigational product, and dispose of the used or unused investigational product;
- Review the completeness of records retained at the study site.

In addition, Shanghai Mingju Biotechnology Co., Ltd reverses the right to temporarily or permanently terminate the study for any reason.

If the study is suspended or discontinued for safety reasons, Shanghai Mingju Biotechnology Co., Ltd will notify the investigators immediately, and will report the suspension or discontinuation of the study along with reasons to the regulatory authorities. In this case, the investigator will be responsible for notifying the IRB/EC immediately and explaining the reasons for suspension or discontinuation of study.

12.9 Termination of Study Site

Shanghai Mingju Biotechnology Co., Ltd reserves the right to terminate a study site at any time for any reasons. The termination and follow-up of the study will be conducted in accordance with the requirements of China's applicable laws and regulations.

12.10 Document Retention

The investigator must retain adequate and accurate records to enable a complete record of the

study progress and verification of the study data. The subjects' records and original medical records, the monitoring visit records, the investigational product inventory, legal documents and letters of Mingju regarding the study must be retained in appropriate form of study documents at each study site. Original medical records include records of all activities, observations and clinical study-related assessments completed for the subjects at hospital visits, as well as all reports and records (including all electronic records). These records will be retained in a secured repository for a period of time until at least 5 years after marketing of the investigational product according to the provisions of the study facility or study site. Prior to the transfer or destruction of these records, Shanghai Mingju Biotechnology Co., Ltd must be notified in writing and given the opportunity to continue to store these documents.

12.11 Confidential Information

Individual subjects and their study data will be identified by a unique study identification code. The names of the subjects will be kept confidential and will not be included in the database. This confidentiality also applies to biological samples and genetic testing, in addition to clinical information related to the subjects. Therefore, the study protocol, documents, data and all other information will be kept strictly confidential. Relevant study information or data may not be disclosed to any unauthorized third party without written permission of the Sponsor. All study results will be stored in the electronic database. The Investigator will maintain the individual subject identification information sheet (subject number and treatment number corresponding to the subject's name) to identify relevant records.

For tracking purposes and chain of custody, the subjects' name and dates of birth will be sent to the Sponsor's personnel responsible for scheduling and production. Such information will also be labeled on the leukapheresis bags and other containers throughout the production of JWCAR029. The above information will be maintained in a separate database with privileged access control and will not be used together with any other clinical information. The database can be accessed only by authorized employees who need to use the above information.

12.12 Publication Plan

Interim data from this study may be presented at academic meetings. Shanghai Mingju Biotechnology Co., Ltd is responsible for preparing the final Clinical Study Report (CSR) as per ICH guidelines and NMPA requirements. The prepared final CSR will include all subjects who signed the ICF (regardless of whether they completed the study or withdrew early)

13 CONTACT INFORMATION

13.1 Emergency Medical Contact

Contact Information of Medical Monitor

Shanghai Mingju Biotechnology Co., Ltd.

JWCAR029Medical@jwtherapeutics.com

13.2 Pharmacovigilance

Pharmacovigilance Contact Information

dMed Biopharmaceutical Co., Ltd

Drugsafety-jw@dmedglobal.com

14 REFERENCES

Adusumilli PS, Cherkassky L, Villena-Vargas J, et al. Regional delivery of mesothelin-targeted CAR T cell therapy generates potent and long-lasting CD4-dependent tumor immunity. Sci Transl Med 2014;6(261):261ra151.

Alexandre V, jordan Gauthier, Kevin A,et,al Factors impacting progression-free survival after CD19-specific CAR-T cell therapy for relapsed/refractory B-cell Non-kodgkin Lymphoma. ASH, 2018;San Diego

Bos R and Sherman LA. CD4+ T-cell help in the tumor milieu is required for recruitment and cytolytic function of CD8+ T lymphocytes. Cancer Res 2010;70(21):8368-8377.

Brentjens RJ, Rivière I, Park JH, et al. Safety and persistence of adoptively transferred autologous CD19-targeted T cells in patients with relapsed or chemotherapy refractory B-cell leukemias. Blood 2011;118(18):4817-4828.

Cheson BD, Fisher RI, Barrington SF, et al. Recommendations for initial evaluation, staging, and response assessment of Hodgkin and non-Hodgkin lymphoma: the Lugano classification. J Clin Oncol 2014;32(27):3059-3068.

Couzin J and Kaiser J. Gene therapy. As Gelsinger case ends, gene therapy suffers another blow. Science 2005;307(5712):1028.

Crump M, Neelapu SS, Farooq U, et al. Outcomes in Refractory Aggressive Diffuse Large B-Cell Lymphoma (DLBCL): Results from the International SCHOLAR-1 Study. ASCO. 2016; Chicago, IL.

Czuczman MS, Davies A, Linton KM, et al. A Phase 2/3 Multicenter, Randomized Study Comparing the Efficacy and Safety of Lenalidomide Versus Investigator's Choice in Relapsed/Refractory DLBCL. Blood 2014;124:628.

Davila ML, Brentjens R, Wang X, et al. How do CARs work? Early insights from recent clinical studies targeting CD19. Oncoimmunology 2012;1(9):1577-1583.

Davila ML, Riviere I, Wang X, et al. Efficacy and toxicity management of 19-28z CAR T cell therapy in B cell acute lymphoblastic leukemia. Sci Transl Med 2014;6(224):224ra225.

Donahue RE, Kessler SW, Bodine D, et al. Helper virus induced T cell lymphoma in nonhuman primates after retroviral mediated gene transfer. J Exp Med 1992;176(4):1125-1135.

Ferlay J, Soerjomataram I, Dikshit R, et al. Cancer incidence and mortality worldwide: Sources, methods and major patterns in GLOBOCAN 2012. Int. J. Cancer 2015; 136, E359–E386.

FDA. Supplemental Guidance on Testing for Replication Competent Retrovirus in Retroviral Vector Based Gene Therapy Products and During Follow-up of Patients in Clinical Trials Using Retroviral Vectors. 2000.

Gardner RA, Finney O, Smithers H, et al. Prolonged functional persistence of CD19 CAR T

cell products of defined CD4:CD8 composition and transgene expression determines durability of MRD-negative ALL remission. ASCO. 2016; Chicago, IL.

Gardner RA, Park JR, KellySpratt KS, et al. T Cell Products of Defined CD4:CD8 Composition and Prescribed Levels of CD19-CAR/EGFRt Transgene Expression Mediate Regression of Acute Lymphoblastic Leukemia in the Setting of Post-Allo-HSCT Relapse. ASH. 2014; San Francisco, CA.

Gardner RA, Finney O, Smithers H, et al. Prolonged functional persistence of CD19 CAR T cell products of defined CD4:CD8 composition and transgene expression determines durability of MRD-negative ALL remission. ASCO. 2016; Chicago, IL

Guo W, Wang SJ, Yang S, et al. A Bayesian interval dose-finding design addressingOckham;s razor: mTPI-2. Contemp Clin Trials 2017;58:23-33.

Grupp SA, Maude SL, Shaw PA, et al. T Cells Engineered with a Chimeric Antigen Receptor (CAR) Targeting CD19 (CTL019) Have Long Term Persistence and Induce Durable Remissions in Children with Relapsed, Refractory ALL. ASH. 2014; San Francisco, CA.

Hacein-Bey-Abina S, Von Kalle C, Schmidt M, et al. LMO2-associated clonal T cell proliferation in two patients after gene therapy for SCID-X1. Science 2003;302(5644):415-419.

Hay,Turtle,JC,et,al Long term follow-up of adult patients with B-cell ALL treated on a phase 1/2 study of CD19 CAR-T cells ASH 2018;San Diego

Jacobsen ED, Sharman JP, Oki Y, et al. Brentuximab vedotin demonstrates objective responses in a phase 2 study of relapsed/refractory DLBCL with variable CD30 expression. Blood 2015;125(9):1394-1402.

Ji Y and Wang SJ. Modified toxicity probability interval design: a safer and more reliable method than the 3+3 design for practical phase I trials. J Clin Oncol 2013;31(14):1785-1791.

Kochenderfer JN, Dudley ME, Kassim S, et al. Chemotherapy-Refractory Diffuse Large B-Cell Lymphoma and Indolent B-Cell Malignancies Can Be Effectively Treated With Autologous T Cells Expressing an Anti-CD19 Chimeric Antigen Receptor. J Clin Oncol 2015;33(6):540-549.

Kochenderfer JN, Kassim SH, Somerville R, et al. Treatment of Chemotherapy-Refractory B-Cell Malignancies with Anti-CD19 Chimeric Antigen Receptor T Cells. ASGCT. 2014a; New Orleans, LA.

Kochenderfer JN, Somerville R, Lu L, et al. Anti-CD19 CAR T Cells Administered after Low-Dose Chemotherapy Can Induce Remissions of Chemotherapy-Refractory Diffuse Large B-Cell Lymphoma. ASH. 2014b; San Francisco, CA.

Lee DW, Gardner R, Porter DL, et al. Current concepts in the diagnosis and management of cytokine release syndrome. Blood 2014;124(2):188-195.

Lee DW, Kochenderfer JN, Stetler-Stevenson M, et al. T cells expressing CD19 chimeric antigen receptors for acute lymphoblastic leukaemia in children and young adults: a phase 1 dose-escalation trial. Lancet 2015;385(9967):517-528.

Li YS, Hayakawa K and Hardy RR. The regulated expression of B lineage associated genes during B cell differentiation in bone marrow and fetal liver. J Exp Med 1993;178(3):951-960.

Li YS, Wasserman R, Hayakawa K, et al. Identification of the earliest B lineage stage in mouse bone marrow. Immunity 1996;5(6):527-535.

Li Z, Dullmann J, Schiedlmeier B, et al. Murine leukemia induced by retroviral gene marking. Science 2002;296(5567):497.

Maude SL, Frey N, Shaw PA, et al. Chimeric antigen receptor T cells for sustained remissions in leukemia. N Eng J Med 2014;371(16):1507-1517.

McGarrity GJ, Hoyah G, Winemiller A, et al. Patient monitoring and follow-up in lentiviral clinical trials. J Gene Med 2013;15:78-82.

Modlich U, Kustikova OS, Schmidt M, et al. Leukemias following retroviral transfer of multidrug resistance 1 (MDR1) are driven by combinatorial insertional mutagenesis. Blood 2005;105(11):4235-4246.

Mounier N, El Gnaoui T, Tilly H, et al. Rituximab plus gemcitabine and oxaliplatin in patients with refractory/relapsed diffuse large B-cell lymphoma who are not candidates for high-dose therapy. A phase II Lymphoma Study Association trial. Haematologica 2013;98(11):1726-1731.

Nagle SJ, Woo K, Schuster SJ, et al. Outcomes of patients with relapsed/refractory diffuse large B-cell lymphoma with progression of lymphoma after autologous stem cell transplantation in the rituximab era. Am J Hematol 2013;88(10):890-894.

NCCN. NCCN Guidelines: Non-Hodgkin's Lymphomas. NCCN Clinical Practice Guidelines in Oncology 2014;1.2014.

Neelapu, et al. Ongoing complete remissions in the Phase 1 of ZUMA-1: A Phase 1-2 multicenter study evaluating the safety and efficacy of KTE-C19 (Anti-CD19 CAR T Cells) in subjects with refractory aggressive B-cell Non-Hodgkin Lymphoma. ASCO 2016 (abstr 7559).

Neelapu, et al. Chimeric antigen receptor T-cell therapy--assessment and management of toxicities. Nat Rev Clin Oncol 2017;148

Oken MM, Creech RH, Tormey DC, et al. Toxicity And Response Criteria Of The Eastern Cooperative Oncology Group. Am J Clin Oncol 1982;5(6):649-655.

Pettengell R, Coiffier B, Narayanan G, et al. Pixantrone dimaleate versus other

chemotherapeutic agents as a single-agent salvage treatment in patients with relapsed or refractory aggressive non-Hodgkin lymphoma: a phase 3, multicentre, open-label, randomised trial. Lancet Oncol 2012;13(7):696-706.

Porter DL, Levine BL, Kalos M, et al. Chimeric antigen receptor-modified T cells in chronic lymphoid leukemia. N Eng J Med 2011;365(8):725-733.

Rigacci L, Puccini B, Cortelazzo S, et al. Bendamustine with or without rituximab for the treatment of heavily pretreated non-Hodgkin's lymphoma patients : A multicenter retrospective study on behalf of the Italian Lymphoma Foundation (FIL). Ann Hematol 2012;91(7):1013-1022.

Rothe M, Modlich U and Schambach A. Biosafety challenges for use of lentiviral vectors in gene therapy. Curr Gene Ther 2013;13(6):453-468.

Sadelain M, Brentjens R and Riviere I. The basic principles of chimeric antigen receptor design. Cancer Discov 2013;3(4):388-398.

Scholler J, Brady TL, Binder-Scholl G, et al. Decade-long safety and function of retroviral-modified chimeric antigen receptor T cells. Sci Transl Med 2012;4(132):132ra153.

Schuster SJ, Svoboda J, Nasta SD, et al. Phase IIa Trial of Chimeric Antigen Receptor Modified T Cells Directed Against CD19 (CTL019) in Patients with Relapsed or Refractory CD19+ Lymphomas. ASH. 2014; San Francisco, CA.

Sehn LH and Gascoyne RD. Diffuse large B-cell lymphoma: optimizing outcome in the context of clinical and biologic heterogeneity. Blood 2015;125(1):22-32.

Shanghai World Lymphoma Awareness Day. (http://baike.baidu.com/view/2102969.html).

Siegel RL, Miller KD and Jemal A. Cancer Statistics 2015. CA Cancer J Clin 2015;65(1):5-29.

Stamenkovic I and Seed B. CD19, the earliest differentiation antigen of the B cell lineage, bears three extracellular immunoglobulin-like domains and an Epstein-Barr virus-related cytoplasmic tail. J Exp Med 1988;168(3):1205-1210.

Sun J, Yang QP, Lu ZH, He MX, el al. Distribution of lymphoid neoplasms in China: Analysis of 4,638 cases according to the World Health Organization classification. Am J Clin Pathol 2012;138:429-434.

Coiffier B, Sarkozy C. Diffuse large B-cell lymphoma: R-CHOP failure-what to do?. Hematology Am Soc Hematol Educ Program 2016(1);366-378.

Telio D, Fernandes K, Ma C, et al. Salvage chemotherapy and autologous stem cell transplant in primary refractory diffuse large B-cell lymphoma: outcomes and prognostic factors. Leuk Lymphoma 2012;53(5):836-841.

Hitz F, Connors JM, Gascoyne RD, et al. Outcome of patients with primary refractory diffuse large B cell lymphoma after R-CHOP treatment. Ann Hematol 2015;94(11):1839-1843.

Crump M, Neelapu SS, Farooq U, et al. Outcomes in refractory diffuse large B-cell lymphoma: results from the international SCHOLAR-1 study. Blood 2017;130(16):1800-1808.

Swerdlow SH, Campo E, Pileri SA, et al. The 2016 revision of the World Health Organization classification of lymphoid neoplasms. Blood 2016;127(20):2375-2390.

Toes RE, Ossendorp F, Offringa R, et al. CD4 T cells and their role in antitumor immune responses. J Exp Med 1999;189(5):753-756.

Turtle, et al. Rate of durable complete response in ALL, NHL, and CLL after immunotherapy with optimized lymphodepletion and defined composition CD19 CAR-T cells. ASCO 2016 (abstr102).

Turtle C, Hanafi L, Berger C, et al. High rates of durable complete response in ALL, NHL and CLL after immunotherapy with optimized lymphodepletion and defined composition CD19 CAR-T cells (JCAR014). ASCO. 2016a; Chicago, IL.

Turtle CJ and Maloney DG. Clinical trials of CD19-targeted CAR-modified T cell therapy; a complex and varied landscape. Expert Rev Hematol 2016b:1-3.

Turtle CJ, Riddell SR and Maloney DG. CD19-targeted chimeric antigen receptor-modified T cell immunotherapy for B cell malignancies. Clin Pharmacol Ther 2016c.

Turtle CJ, Sommermeyer D, Berger C, et al. Therapy of B Cell Malignancies with CD19-Specific Chimeric Antigen Receptor Modified T Cells of Defined Subset Composition. ASH. 2014; San Francisco, CA.

Van Den Neste E, Schmitz N, Mounier N, et al. Outcome of patients with relapsed diffuse large B-cell lymphoma who fail second-line salvage regimens in the International CORAL study. Bone Marrow Transplant 2016;51(1):51-57.

Wang GP, Levine BL, Binder GK, et al. Analysis of lentiviral vector integration in HIV+ study subjects receiving autologous infusions of gene modified CD4+ T cells. Mol Ther 2009;17(5):844-850.

Wang M, Fowler N, Wagner-Bartak N, et al. Oral lenalidomide with rituximab in relapsed or refractory diffuse large cell, follicular and transformed lymphoma: a phase II clinical trial. Leukemia 2013;27(9):1902-1909.

Wang YC, Wei LJ, Liu JT, Li SX, Wang QS. Comparison of cancer incidence between China and the USA 2012; Cancer Biol Med 2012; 9: 128-132.

Wilson W. Treatment strategies for aggressive lymphomas: what works? Am Soc Hematol Educ Program 2013:584-590.

Yuan YN, Yang L, Sun TT, Li HC, Zhu J, Song YQ, Wang N. The incidence of malignant lymphoma in Beijing residence during 1998-2010. Chinese Journal of Prevention Medicine 2014; 48(8): 669-674.

Yuan Ji, Yisheng Li, B.Nebiyou Bekele. Dose-finding in phase I clinical trials based on toxicity probability intervals. Clinical Trials 2007; 4:235-244.

Yuan Ji, Ping Liu, Yisheng Li, B.Nebiyou Bekele. A modified toxicity probability interval method for dose-finding trials. Clinical Trials 2010;7:653-663.

Yuan Ji, Sue-Jane Wang. Modified Toxicity Probability Interval Design: A safer and more reliable method than the 3+3 design for practical phase I trials. Journal of clinical oncology 2013;31(14):1785-1791.

Wentian Guo, Sue-Jane Wang, Shengjie Yang, Suiheng Lin, Yuan Ji. A bayesian interval dose-finding design addressing ockham's razor: mTPI-2. Contemp Clin Trials 2017; 58:23-33.

APPENDIX A SCHEDULE OF ACTIVITIES (SOA)

Observation/Procedure	Before Treatment		Treatment Period										After Treatment						
	Screening	Evaluation	Product Infusion										Follow-up Observation, EOS [15]						
Period of Study	-28		-7 to -2 [1, 13]	1d	2	3	4	8	11	15	22	29	60	90	180	270	365	545	730
Visit Window							+1	±1	±1	±2	±2	±2	±14	±14	±14	±14	±14	±14	±14
Procedures																			
Informed consent	×																		
Inclusion/Exclusion Criteria	×																		
Medical History [22]	×																		
12-lead ECG		×[19]																	
MUGA/ECHO [23]	×																		
Testing for Infectious Disease [24]	×																		
Serum Pregnancy Test (Women of Childbearing Potential)	×	×[14]																	
IPI Score		×																	
Leukapheresis		×																	
Lympho-depleting Chemotherapy [25]			×[3]																
JWCAR029 Infusion				×[2]															
AEs/SAEs	AEs/SAEs related to study procedures		All AEs/SAEs (either due to lympho-depleting chemotherapy or JWCAR029) will be collected through start of lympho-depleting chemotherapy to 90 days after JWCAR029 infusion [12]												AEs/SAEs related to JWCAR029 [4,12]				
Concomitant Medications	Concomitant medications for the treatment of AEs/SAEs related to the study procedures		Concomitant medications used during the period from start of lympho-depleting chemotherapy to 90 days after JWCAR029 infusion will be collected												Record and report:<br>• Corticosteroids<br>• Drugs used to treat GVHD<br>• Anticancer therapy<br>• Drugs used to treat AE/SAE related to JWCAR029				
Height and Weight		×[14]		×															
Physical Examination	×	×[14]		×				×	×	×	×	×	×[5]	×[5]	×[5]	×[5]	×		×

Observation/Procedure	Before Treatment		Treatment Period										After Treatment						
	Screening	Evaluation	Product Infusion										Follow-up Observation, EOS [15]						
Period of Study	-28		-7 to -2 [1, 13]	1d	2	3	4	8	11	15	22	29	60	90	180	270	365	545	730
Visit Window							+1	±1	±1	±2	±2	±2	±14	±14	±14	±14	±14	±14	±14
Procedures																			
Vital Signs [26]		×[14]	×	×	×	×	×	×	×	×	×	×							
ECOG	×	×[14]	×	×				×	×	×	×	×							
Neurological Assessments [27]	×	×[14]		×	×	×	×	×	×	×	×	×		×[5]					
MMSE		×[14]		×	×	×	×	×	×	×		×		×[5]					
CSF and CNS Imaging [27]	×																		
PET-CT/Enhanced CT Scan	×[6]	×[19, 20]										×[7]		×[8]	×[8]	×[8]	×[8]	×[8]	×[8]
Hematology and Blood Biochemistry [28]	×	×[14]	×	×	×	×	×	×	×	×	×	×	×[5]	×[5]	×[5]	×[5]	×[5]	×[5]	×[5]
Coagulation Function [29]		×[14]		×	×	×	×	×	×	×	×	×	×[5]	×[5]	×[5]	×[5]	×[5]	×[5]	×[5]
Inflammatory Markers [30]		×[14]		×	×	×	×	×	×	×	×[9]	×[9]	×[5]	×[5]	×[5]	×[5]	×[5]	×[5]	×[5]
Immunoglobulins [31]		×[14]								×	×	×	×[18]	×[18]	×[18]	×[18]	×[18]	×[18]	×[18]
Diagnostic Bone Marrow Aspiration/Biopsy		×[19]										×[21]							
Fresh Tumor Biopsy [11]		×[19]							×[10]										
Immunogenicity Testing [32, 35]		×								×		×	×[5]	×[5]	×[5]	×[5]	×[5]	×[5]	×[5]
PK/PD by Flow Cytometry [33]		×		×	×	×	×	×	×	×	×	×	×[5]	×[5]	×[5]	×[5]	×[5]		
qPCR to detect the transgene copy number of JWCAR029 [33, 35]		×		×	×	×	×	×	×	×	×	×	×	×	×	×	×[17]	×[16, 17]	×[16, 17]
Cytokine Testing [34, 35]		×		×	×	×	×	×	×	×	×	×	×[5]	×[5]	×[5]	×[5]	×[5]	×[5]	×[5]

Notes:

1) All assessments and laboratory tests must be performed prior to lympho-depleting chemotherapy or JWCAR029 infusion

2) There is a possibility that the subjects may receive infusion of non-standard JWCAR029, and any subjects receiving a non-standard investigational product will be included in the safety set for analysis. All subjects will be followed up as per the study protocol

3) Days of dosing are approximate values. Lympho-depleting chemotherapy should be completed for the subjects 2 to 7 days prior to JWCAR029 infusion

4) Through 90 days after JWCAR029 infusion until the EOS visit

5) Not applicable to subjects receiving subsequent alternative anticancer therapy

6) If no anticancer therapy, the results of prior PET-CT scan can be used for confirming the eligibility criterion of PET+ disease

7) PET-CT and contrast-enhanced CT scans may be performed on Day 22 to Day 29

8) If CR achieved, enhanced CT scan is required for confirmation PET-CT scans are no longer required for subjects who have progressed/relapsed or started subsequent anticancer therapy. PET scan should be performed to confirm PD

9) As clinically indicated

10) Specimens may be collected between Days 8 to 14

11) Applicable to subjects with palpable mass and as deemed feasible by the investigator

12) Any of the following clinical symptoms, if observed, should be reported as SAE, unless the event can be clearly attributed to another cause: new/secondary malignancy; new incidence or exacerbation of a pre-existing neurologic disorder; new incidence of a prior rheumatologic or other autoimmune disorder; new incidence of a hematologic disorder; rare and unexpected disorders with unknown etiology (e.g., Guillian-Barre syndrome, Stevens-Johnson syndrome). For subjects who start subsequent non-chemotherapeutic anticancer therapy (e.g., checkpoint inhibitors, immunomodulators) within 90 days after JWCAR029 infusion, all AEs/SAEs will be collected until 90 days after JWCAR029 infusion or 30 days after start of subsequent non-chemotherapeutic anticancer therapy, whichever is longer

13) Assessment will be performed daily during the period of lympho-depleting chemotherapy

14) Should be performed within 7 days prior to the start of lympho-depleting chemotherapy, except for serum pregnancy test, which should be performed within 48 hours prior to lympho-depleting chemotherapy. Assessment should be performed after start of any anticancer therapy for control of tumors

15) Performed at the EOS visit for subjects who have withdrawn for more than 2 months after Day 90 but before Day 180

16) Not required for subjects without a detected transgene copy number in previous test

17) If more than 1% of the cells in the test samples collected at Day 365 or subsequent visit are positive for transgene copy number, the pattern of vector integration sites will be analyzed. If a predominant integration site is detected, the subjects will be asked to provide blood samples 3 months later for follow-up testing

18) Not required if no recent IVIG treatment and documented B-cell recovery

19) For subjects who received anticancer therapy for disease control during the period of JWCAR029 preparation, these assessments must be performed after completion of anticancer therapy and as close as possible to the start of lympho-depleting chemotherapy (recommended within 7-14 days prior to the start of chemotherapy)

20) It is recommended that the assessment be performed within 7 days before the start of lympho-depleting chemotherapy, and it must be completed within 6 weeks before the start of lympho-depleting chemotherapy; not required if the subject received no anticancer therapy and underwent the scan at screening at the study site

21) For patients with a CR on radiological imaging, diagnostic bone marrow aspiration/biopsy (BMA/BMB) for morphological assessment is required if presence of bone marrow involvement at baseline; immunohistochemistry is required if presence of tumor cells, including CD19

22) Medical history, including diagnosis and current disease, with or without HSCT, history of chemotherapy, radiotherapy and/or surgery, and history of prior gene therapy. This may also include history of previous treatment toxicity and allergy

23) Echocardiogram (ECHO) or multiple uptake gated acquisition (MUGA)

24) Testing for infectious disease includes HIV, syphilis, HBV (HBsAg, HBsAb, HBcAb), and HCV (HCVAb)

25) The recommended chemotherapy regimen is as follows: first 1 L of 0.9% NaCl IV drip at a rate of 500 mL/h; fludarabine 25 mg/m^2 IV drip over at least 30 minutes; cyclophosphamide 250 mg/m^2 IV drip over at least 60 minutes; 1 L of 0.9% NaCl IV drip at a rate of 500 mL/h

26) Including blood pressure (BP), respiratory rate (RR), heart rate (HR), body temperature, and oxygen saturation by fingertip pulse oximeter. A measurement should be performed 15 minutes before, during and 15 minutes after JWCAR029 infusion. The vital signs are measured every 15 minutes in the first hour after infusion and then hourly in the second and third hours, until the vital signs have become stable

27) Neurological assessment mainly includes alerted state of consciousness, alerted mental status, speech disorder, ataxia and gait disturbance, motor muscle strength abnormalities, cranial nerve abnormalities, epilepsy, tremors, muscle spasms, etc. If clinically indicated (e.g., CNS symptoms or presence of suspected CNS lymphoma, or changes of relevant symptoms or vital signs), CSF examination and CNS imaging (including but not limited to CT, MRI) should be performed both before and after administration of JWCAR029. CSF examination includes cell count with differential; CSF culture (bacterial, fungal, viral) should be performed for subjects with suspected infection.

28) Hematology includes red blood cell count, white blood cell count, platelet count, neutrophil count, lymphocyte count, monocyte count and hemoglobin content, blood biochemistry includes blood glucose; blood biochemistry include glucose, blood urea (nitrogen), creatinine, uric acid, sodium, potassium, chloride, calcium, magnesium, phosphorus, total protein, albumin, total and direct bilirubin, alkaline phosphatase, creatine kinase and its isoenzymes, lactate dehydrogenase, ALT, AST, total cholesterol, and triglycerides

29) Coagulation function include prothrombin time (PT), activated partial thromboplastin time (aPTT), international normalized ratio (INR), fibrinogen and D-dimer

30) Inflammatory markers include C-reactive protein (CRP) and ferritin

31) Immunoglobulins include IgA, IgG, and IgM

32) Peripheral blood samples will be collected after JWCAR029 infusion for humoral immunogenicity assessment, including anti-drug antibodies (ADA) binding to the extracellular domain of JWCAR029 in plasma

33) Peripheral blood specimens may be used for flow cytometry to detect the number of JWCAR029 cells and immune cell subsets in the specimens, and for qPCR to detect the transgene copy number of JWCAR029 in the specimens.

34) Serum cytokines associated with CRS

35) Samples need to be collected before lympho-depleting chemotherapy as baseline control for pre-chemotherapy vs. post-chemotherapy and before CAR+ T cell therapy

APPENDIX B RESPONSE CRITERIA

Response Category	PET-CT-based Criteria
Complete Response (CR)	Score of 1, 2 or 3 [a], with or without a residual mass<br>No sign of FDG-avid lesion in the bone marrow
Partial Response (PR)	Score of 4 or 5 [a] with reduced uptake compared with baseline and residual mass(es) of any size<br>Residual uptake higher than uptake in normal marrow but reduced compared with baseline (diffuse uptake compatible with reactive changes from chemotherapy allowed). If there are persistent focal changes in the marrow in the context of a nodal response, consideration should be given to further evaluation with MRI or biopsy or an interval scan.
Stable Disease (SD)	Score of 4 or 5 [a], with no significant change in FDG uptake from baseline<br>No change in bone marrow from baseline
Progressive Disease (PD)	Score of 4 or 5 [a], with an increase in intensity of uptake from baseline<br>New FDG-avid foci consistent with lymphoma (biopsy or repeat scan may be required if the etiology of the foci is uncertain)

a Based on the Deauville 5-point scale

1: No uptake;

2: Uptake ≤ mediastinum;

3: Uptake > mediastinum but ≤ liver;

4: Moderately increased uptake > liver;

5: Markedly increased uptake > liver and new sites of lesions related to lymphoma;

X: New areas of uptake unlikely to be related to lymphoma.

APPENDIX C COCKCROFT-GAULT FORMULA FOR CALCULATION OF ESTIMATED CREATININE CLEARANCE

Units of Serum Creatinine	Sex	Estimated Creatinine Clearance (mL/min)
**mg/dL**	Male	$\frac{(140 - \text{Subject's age [years]}) \times \text{Weight (kg)}}{72 \times \text{Serum Creatinine (mg/dL)}}$
	Female	$\frac{(140 - \text{Subject's age [years]}) \times \text{Weight (kg)} \times 0.85}{72 \times \text{Serum Creatinine (mg/dL)}}$
**μmol/L**	Male	$\frac{(140 - \text{Subject's age [years]}) \times \text{Weight (kg)} \times 1.23}{\text{Serum Creatinine (μmol/L)}}$
	Female	$\frac{(140 - \text{Subject's age [years]}) \times \text{Weight (kg)} \times 1.04}{\text{Serum Creatinine (μmol/L)}}$

APPENDIX D MINI-MENTAL STATE EXAMINATION

Mini-Mental State Examination (MMSE)

Patient Number_____________		Visit Time_____________
**Orientation**	Score	Maximum
What is the day? the date? the months? the season? the year?	( )	5
Where are we: province? City? Hospital? Department? Floor?	( )	5
**Registration**	( )	3
Now I'm going to name three objects, and when I'm done, you will be asked to repeat them. Please remember all the three objects, because I will ask you several minutes later. (Say the names of three objects clearly and slowly, allowing approximately one second for each).		
"Ball", "National flag", "Tree"		
Please name each of the three objects (score based on the first response)		
**Attention and Calculation**	( )	5
I would like you to count backward from 100 by sevens and tell me the answer after each subtraction by seven, until I say "Stop". (If it is wrong but the next answer is correct, record one wrong answer only).		
93 86 79 72 65		
**Recall**	( )	3
Earlier I told you the names of three things. Can you tell me what those were? "Ball", "National flag" and "Tree"		
**Language**		
(Show the patient a wrist watch and ask the patient) What is this?	( )	1
(Show the patient a pen and ask the patient) What is this?	( )	1
Now I'm going to say a sentence, please follow me and repeat it clearly.		
"Forty-four stone lions"	( )	1
I will give you a piece of blank paper, please do as I'm telling you. Start now: "Take the paper in your right hand, fold it in half, and put it on your leg". (Do not repeat instructions, nor demonstrate how)	( )	3
Please read this sentence and do what it says. (See the reverse)	( )	1
Please write a sentence for me. (The sentence must contain a subject, a verb and an object)	( )	1
Please write down the complete sentence.________________________	( )	1
(See the reverse) This is a picture, please copy the figure exactly as it is on the paper		
(Correct: A figure of two pentagons with a quadrilateral at the intersection)		

Score between 27 and 30: Normal

Total ( )

Score < 27: cognitive impairment

Clinical impression:______________________

Hospital Name: **Date**: **Rated by Physician:**

"Close your eyes"

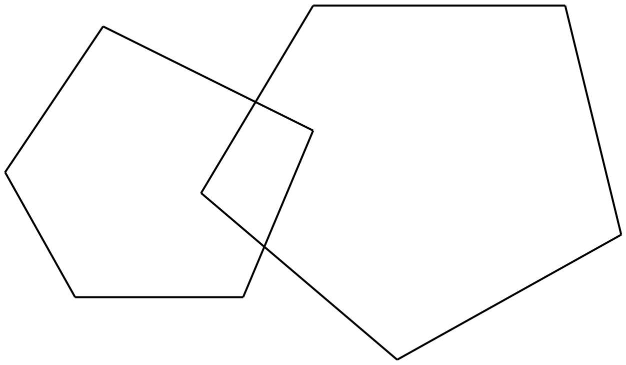

APPENDIX E IPI (INTERNATIONAL PROGNOSTIC INDEX) SCORE

**Variables**		**Score**
Age		
ECOG	☐ 0<br>☐ 1<br>☐ 2<br>☐ 3<br>☐ 4	
Ann arbor stage	☐ I<br>☐ II<br>☐ III<br>☐ IV	
LDH	☐ Normal<br>☐ Above normal	
Number of extranodal sites of involvement		
Total Score		

Appendix: International Prognostic Index (IPI) for NHL

Variables	0	1
Age	≤ 60 years	> 60 years
ECOG	0 or 1	2, 3, 4
Ann arbor stage	I or II	III or IV
LDH	Normal	Above normal
Number of extranodal sites of involvement	< 2	≥ 2

APPENDIX F FORMULA FOR CALCULATION OF BODY SURFACE AREA (BSA)

DuBois formula: $\text{BSA}\ (\text{m}^2) = 0.007184 \times \text{Weight (kg)}^{0.425} \times \text{Height (cm)}^{0.725}$
