# Supplementary material for: Cholesterol efflux from C1QB-expressing macrophages is associated with resistance to chimeric antigen receptor T cell therapy in primary refractory diffuse large B cell lymphoma
Source: Nat Commun. 2024 Jun 18;15:5183. doi: 10.1038/s41467-024-49495-4 (PMC11189439; doi:10.1038/s41467-024-49495-4)
Supplement: Supplementary file 3 — Reporting Summary [file 41467_2024_49495_MOESM3_ESM.pdf]

nature portfolio

Corresponding author(s): Weili Zhao

Last updated by author(s): 3/21/2024

Reporting Summary

Nature Portfolio wishes to improve the reproducibility of the work that we publish. This form provides structure for consistency and transparency in reporting. For further information on Nature Portfolio policies, see our Editorial Policies and the Editorial Policy Checklist.

Statistics

For all statistical analyses, confirm that the following items are present in the figure legend, table legend, main text, or Methods section.

n/a	Confirmed	
☐	☒	The exact sample size (*n*) for each experimental group/condition, given as a discrete number and unit of measurement
☒	☐	A statement on whether measurements were taken from distinct samples or whether the same sample was measured repeatedly
☐	☒	The statistical test(s) used AND whether they are one- or two-sided<br>*Only common tests should be described solely by name; describe more complex techniques in the Methods section.*
☐	☒	A description of all covariates tested
☐	☒	A description of any assumptions or corrections, such as tests of normality and adjustment for multiple comparisons
☐	☒	A full description of the statistical parameters including central tendency (e.g. means) or other basic estimates (e.g. regression coefficient) AND variation (e.g. standard deviation) or associated estimates of uncertainty (e.g. confidence intervals)
☐	☒	For null hypothesis testing, the test statistic (e.g. *F*, *t*, *r*) with confidence intervals, effect sizes, degrees of freedom and *P* value noted<br>*Give P values as exact values whenever suitable.*
☒	☐	For Bayesian analysis, information on the choice of priors and Markov chain Monte Carlo settings
☒	☐	For hierarchical and complex designs, identification of the appropriate level for tests and full reporting of outcomes
☒	☐	Estimates of effect sizes (e.g. Cohen's *d*, Pearson's *r*), indicating how they were calculated

Our web collection on statistics for biologists contains articles on many of the points above.

Software and code

Policy information about availability of computer code

Data collection	Images of immunofluorescence were captured with anSP8, Leica Microsystems or LSM780, Zeiss Microscopy.<br>Collection of flow cytometry cells were by BD LSRFortessa (BD Biosciences, Franklin Lake, NJ, USA)<br>The source code for data cleaning and analysis is accessible for scientific research purposes on GitHub (https://github.com/MikaQiao/scDLBCL) and Zenodo (https://zenodo.org/doi/10.5281/zenodo.10720059). The remaining data are available within the Article, Supplementary Information, or Source Data file.
Data analysis	Cell Ranger (version 6.0.1) https://support.10xgenomics.com/single-cell-gene-expression/software/pipelines/latest/what-is-cell-ranger<br>Seurat (version 4.0.4) https://satijalab.org/seurat/<br>DoubletFinder (version 2.0.3) https://github.com/chris-mcginnis-ucsf/DoubletFinder<br>harmony (version 1.0) https://github.com/immunogenomics/harmony<br>clustree (version 0.4.4) https://cran.r-project.org/web/packages/clustree/index.html<br>Monocle 2 (version 2.18.0) http://cole-trapnell-lab.github.io/monocle-release/<br>clusterProfiler (version 4.1.4) https://bioconductor.org/packages/release/bioc/html/clusterProfiler.html<br>Cellchat (version 1.1.3) https://bioconductor.org/packages/release/bioc/html/CellChat.html<br>ggplot2 (version 3.3.5) https://ggplot2.tidyverse.org/<br>ComplexHeatmap (version 2.6.2) https://bioconductor.org/packages/release/bioc/html/ComplexHeatmap.html<br>LAS X LS(https://www.leica-microsystems.com/products/microscope-software/p/leica-las-x-ls/) and ZEN Microscopy Software (https://www.zeiss.com/microscopy/en/products/software/) for immunofluorescence statistical analysis.<br>FlowJo software (V10) for Flow cytometry statistical analysis (https://www.flowjo.com/solutions/flowjo/)

For manuscripts utilizing custom algorithms or software that are central to the research but not yet described in published literature, software must be made available to editors and reviewers. We strongly encourage code deposition in a community repository (e.g. GitHub). See the Nature Portfolio guidelines for submitting code & software for further information.

Data

Policy information about availability of data

All manuscripts must include a data availability statement. This statement should provide the following information, where applicable:

- Accession codes, unique identifiers, or web links for publicly available datasets
- A description of any restrictions on data availability
- For clinical datasets or third party data, please ensure that the statement adheres to our policy

The raw sequencing data generated during this study have been deposited in the Genome Sequence Archive in National Genomics Data Center, China National Center for Bioinformation/Beijing Institute of Genomics, Chinese Academy of Science (https://ngdc.cncb.ac.cn/gsa-human), with accession number 'GSA-Human: HRA006798'. These data are under controlled access by human privacy regulations and are only available for research purposes. Access to the data can be granted following approval from the Data Access Committee of the GSA-human database, as detailed at https://ngdc.cncb.ac.cn/gsa-human/document/GSA-Human_Request_ Guide_for_Users_us.pdf.

Research involving human participants, their data, or biological material

Policy information about studies with human participants or human data. See also policy information about sex, gender (identity/presentation), and sexual orientation and race, ethnicity and racism.

Reporting on sex and gender

12 patients were in our study,of which 8(66.7%) were males and 4(33.3%) females.

Reporting on race, ethnicity, or other socially relevant groupings

no

Population characteristics

Between July 2020 and June 2021, 19 patients were screened, and 14 patients who fulfilled the eligibility criteria were enrolled and subjected to leukapheresis (Fig. 1a). Relma-cel was successfully administered to 12 patients with a single infusion of 100 × 106 CAR-T cells. Notably, one patient (P002) was excluded from efficacy analysis due to a secondary-onset tumor (Hodgkin's lymphoma) at 13 months.The median time from enrollment to infusion was 34.5 days (range, 27–61 days). Patients' characteristics are shown in Supplementary Table 1. Four patients (33.3%) had sum of perpendicular diameters (SPDs) of ≥5000 mm2 before CAR-T cell therapy, and five patients (41.7%) received bridging therapy with second-line chemotherapy: three patients received the ICE regimen (etoposide, ifosfamide, and carboplatin), one received R-ICE (rituximab, etoposide, ifosfamide, and carboplatin), and one received the combination of dexamethasone, cyclophosphamide, and vindesine.

Recruitment

Between July 2020 and June 2021, 19 patients were screened, and 14 patients who fulfilled the eligibility criteria were enrolled and subjected to leukapheresis (Fig. 1a). Relma-cel was successfully administered to 12 patients with a single infusion of 100 × 106 CAR-T cells. Notably, one patient (P002) was excluded from efficacy analysis due to a secondary-onset tumor (Hodgkin's lymphoma) at 13 months.The median time from enrollment to infusion was 34.5 days (range, 27–61 days). Patients' characteristics are shown in Supplementary Table 1. Four patients (33.3%) had sum of perpendicular diameters (SPDs) of ≥5000 mm2 before CAR-T cell therapy, and five patients (41.7%) received bridging therapy with second-line chemotherapy: three patients received the ICE regimen (etoposide, ifosfamide, and carboplatin), one received R-ICE (rituximab, etoposide, ifosfamide, and carboplatin), and one received the combination of dexamethasone, cyclophosphamide, and vindesine.

Ethics oversight

The use of samples for this study was approved by ethical permission from the National Research Ethics Committee (REC:2019-112). Appropriate approvals and informed writtern consent for study participation were obtained and the study was performed in accordance with the Declaration of Helsinki.

Note that full information on the approval of the study protocol must also be provided in the manuscript.

Field-specific reporting

Please select the one below that is the best fit for your research. If you are not sure, read the appropriate sections before making your selection.

[x] Life sciences [ ] Behavioural & social sciences [ ] Ecological, evolutionary & environmental sciences

For a reference copy of the document with all sections, see nature.com/documents/nr-reporting-summary-flat.pdf

Life sciences study design

All studies must disclose on these points even when the disclosure is negative.

Sample size	This clinical study reflects an exploratory phase I clinical trial with safety as the primary endpoint, and detecting significant safety issues associated with treatment is the primary focus. our sample size is 12, which is reasonable to ensure detection of significant safety issues associated with treatment.
Data exclusions	No participants were excluded in this clinical study reflects an exploratory phase I clinical trial with safety as the primary endpoint.
Replication	We confirmed that all attempts to replicate experiments were successful. All experiments were performed for at least 2-3 biological replicates, which were specified in the figure legends. To determine statistical significance, at least three biological replicates were used
Randomization	Non-randomized Controlled Trial.
Blinding	Not applicable.

Reporting for specific materials, systems and methods

We require information from authors about some types of materials, experimental systems and methods used in many studies. Here, indicate whether each material, system or method listed is relevant to your study. If you are not sure if a list item applies to your research, read the appropriate section before selecting a response.

Materials & experimental systems

n/a	Involved in the study	
☐	☒	Antibodies
☒	☐	Eukaryotic cell lines
☒	☐	Palaeontology and archaeology
☒	☐	Animals and other organisms
☐	☒	Clinical data
☒	☐	Dual use research of concern
☒	☐	Plants

Methods

n/a	Involved in the study	
☒	☐	ChIP-seq
☐	☒	Flow cytometry
☒	☐	MRI-based neuroimaging

Antibodies

Antibodies used

FITC anti-human CD4 (566320, BD Biosciences)
BV421 anti-human CD4 (565997, BD Biosiences),
BV421 anti-human PD1 (562516, BD Biosiences),
APC-R700 anti-human LAG3 (565774, BD Biosiences),
BV786 anti-human CD3 (563799, BD Biosiences),
PerCP-Cy5.5 anti-human CD8 (565310, BD Biosiences),
APC anti-human CD206 (550889, BD Biosiences),
PE-Cy7 anti-human CD68 (565595, BD Biosiences),
BV605 anti-human CD11b (562721, BD Biosiences)

Validation

FITC anti-human CD4 (566320, BD Biosiences), validation stated on supplier's website: https://www.bdbiosciences.com/zh-cn/products/reagents/flow-cytometry-reagents/research-reagents/single-color-antibodies-ruo/fitc-mouse-anti-human-cd4.566320;
BV421 anti-human CD4 (565997, BD Biosiences), validation stated on supplier's website: https://www.bdbiosciences.com/zh-cn/products/reagents/flow-cytometry-reagents/research-reagents/single-color-antibodies-ruo/bv421-mouse-anti-human-cd4.565997;
BV421 anti-human PD1 (562516, BD Biosiences), validation stated on supplier's website: https://www.bdbiosciences.com/zh-cn/products/reagents/flow-cytometry-reagents/research-reagents/single-color-antibodies-ruo/bv421-mouse-anti-human-cd279-pd-1.562516;
APC-R700 anti-human LAG3 (565774, BD Biosiences), validation stated on supplier's website: https://www.bdbiosciences.com/zh-cn/products/reagents/flow-cytometry-reagents/research-reagents/single-color-antibodies-ruo/apc-r700-mouse-anti-human-lag-3-cd223.565774;
BV786 anti-human CD3 (563799, BD Biosiences), validation stated on supplier's website: https://www.bdbiosciences.com/zh-cn/products/reagents/flow-cytometry-reagents/research-reagents/single-color-antibodies-ruo/bv786-mouse-anti-human-cd3.563799;
PerCP-Cy5.5 anti-human CD8 (565310, BD Biosiences), validation stated on supplier's website: https://www.bdbiosciences.com/zh-cn/products/reagents/flow-cytometry-reagents/research-reagents/single-color-antibodies-ruo/percp-cy-5-5-mouse-anti-human-cd8.565310;
APC anti-human CD206 (550889, BD Biosiences), validation stated on supplier's website: https://www.bdbiosciences.com/zh-cn/products/reagents/flow-cytometry-reagents/research-reagents/single-color-antibodies-ruo/apc-mouse-anti-human-cd206.550889;
PE-Cy7 anti-human CD68 (565595, BD Biosiences), validation stated on supplier's website: https://www.bdbiosciences.com/zh-cn/search-results?searchKey=PE-Cy7%20anti-human%20CD68;

BV605 anti-human CD11b (562721, BD Biosciences), validation stated on supplier's website: https://www.bdbiosciences.com/zh-cn/products/reagents/flow-cytometry-reagents/research-reagents/single-color-antibodies-ruo/bv605-mouse-anti-human-cd11b.562721.

Clinical data

Policy information about clinical studies

All manuscripts should comply with the ICMJEguidelines for publication of clinical research and a completedCONSORT checklist must be included with all submissions.

Clinical trial registration	The study was registered at www.chinadrugtrials.org.cn (CTR20200376).
Study protocol	The study protocol has been uploaded to Nature Communications as supplementary info in previous submission.
Data collection	Between July 2020 and June 2021, 19 patients were screened, and 14 patients who fulfilled the eligibility criteria were enrolled and subjected to leukapheresis. Relma-cel was successfully administered to 12 patients with a single infusion of 100 × 106 CAR-T cells. all data in Ruijin Hospital Affiliated to Shanghai Jiao Tong University School of Medicine, Shanghai,China.
Outcomes	This multicenter phase I trial enrolling only patients with primary refractory DLBCL met the primary endpoint of manageable safety and demonstrated encouraging improvements in PFS and OS.

Plants

Seed stocks	Not applicable.
Novel plant genotypes	Not applicable.
Authentication	Not applicable.

Flow Cytometry

Plots

Confirm that:

☒ The axis labels state the marker and fluorochrome used (e.g. CD4-FITC).

☒ The axis scales are clearly visible. Include numbers along axes only for bottom left plot of group (a 'group' is an analysis of identical markers).

☒ All plots are contour plots with outliers or pseudocolor plots.

☒ A numerical value for number of cells or percentage (with statistics) is provided.

Methodology

Sample preparation	Cells were prewashed with PBS and incubated with antibodies for 30 min on ice. After washing twice, resuspended in 500 µL cellular preservation fluids were run on BD LSRFortessa (BD Biosciences, Franklin Lake, NJ, USA) and analyzed using FlowJo software.
Instrument	BD LSRFortessa (BD Biosciences, Franklin Lake, NJ, USA)
Software	FlowJo software.
Cell population abundance	The purity was verified by flow cytometry.
Gating strategy	The initial cell population was gated on an SSC-A/FSC-A plot. Cell singlets were identified through FSC-A/FSC-H gating. Positive cell populations were determined using specific antibodies, and the cell population within the gate was further analyzed based on the expression of specific markers.

☒ Tick this box to confirm that a figure exemplifying the gating strategy is provided in the Supplementary Information.
